# Comparison of the accuracy of intraoral scanners, intraoral cameras, radiographs, and histological methods for the diagnosis of dental caries: a systematic review and meta-analysis

**DOI:** 10.1038/s41405-025-00345-5

**Published:** 2025-10-13

**Authors:** Farah Rashid, Taseef Hasan Farook, James Dudley

**Affiliations:** Adelaide Dental School, The University of Adelaide, Adelaide, SA 5000 Australia

**Keywords:** Caries risk assessment, Oral diagnosis

## Abstract

**Objective:**

This systematic review and meta-analysis compare the diagnostic accuracy of intraoral scanners (IOS) and intraoral cameras (IOC) against traditional radiographic and histological methods for caries detection. Due to variation in sensitivity (SE) and specificity (SP) among diagnostic tools, this study evaluated their performance based on lesion type, lesion location, and examiner-dependent factors.

**Methods:**

A comprehensive literature search was conducted using keyword-based search strings in Scopus, Web of Science, PubMed, Cochrane Library, and Dentistry & Oral Sciences Source, following PRISMA-DTA guidelines. Studies comparing IOS or IOC to radiographic or histological reference standards were included. Three independent reviewers assessed study quality using the Joanna Briggs Institute (JBI) Critical Appraisal Checklist, with disagreements resolved by discussion and Fleiss’s kappa (κ) analysis. Meta-analysis was performed using a bivariate random-effects model to estimate pooled SE and SP. Subgroup analyses examined the impact of lesion type, lesion location, and examiner-dependent variabilities, using the Python programming language.

**Results:**

Twenty-eight studies met the inclusion criteria, with 16 studies included in the meta-analysis. Pooled SE and SP of IOS and IOC were 50%, indicating moderate diagnostic accuracy. Radiographic methods had the highest SP, making them more effective at avoiding false positives. In contrast, IOSs and IOCs demonstrated higher SE for occlusal and dentin lesions, suggesting their utility in early caries detection. Lesion location significantly influenced diagnostic accuracy, with IOS and IOC showing superior SE for occlusal lesions but lower SE for supragingival lesions. Examiner-dependent differences in SE reached up to 32%, emphasizing the need for structured training and calibration protocols.

**Conclusions:**

IOSs and IOCs showed promise for early caries detection due to their higher SE for occlusal and dentin lesions. However, their lower SP compared to radiographic methods raises concerns about false positives. Standardized examiner training and improved diagnostic protocols are essential to enhance the reliability of these digital imaging techniques.

## Introduction

### Rationale

Dental caries is the most widespread noncommunicable disease globally, posing a significant burden on public health across the lifespan [1]. However, a universally reliable diagnostic method capable of detecting all types of carious lesions remains elusive [2]. Traditional approaches, such as radiographic and visual-tactile examinations are commonly used but are associated with intra- and inter-clinical variability, limiting their diagnostic accuracy [3]. While large-scale epidemiological studies continue to rely on visual methods [4], their reproducibility is limited by the inability to monitor lesion progression or distinguish between active and inactive lesions in a single visit.

To enhance diagnostic consistency, various standardized visual caries scoring systems have been developed [5], including the International Caries Detection and Assessment System (ICDAS) [6, 7], Universal Visual Scoring System (UniViSS) [8, 9], American Dental Association (ADA) [10], and modified numerical caries scoring systems [11–13]. However, inconsistencies in the criteria and implementation continue to hamper standardization and reliability [14]. Radiographic techniques, though widely used, involve ionizing radiation and often underestimate lesion depth, particularly in early-stage caries. Their low sensitivity (SE) in detecting incipient caries further limits their effectiveness in early intervention strategies [7, 9, 15].

Alternative imaging technologies - such as fiber-optic transillumination (FOTI), quantitative light-induced fluorescence (QLF), digital image fiber optic transillumination (DiFOTI), laser fluorescence (LF), and optical coherence tomography (OCT) – allow for microstructural detection of caries [16]. However, widespread clinical adoption remains limited due to procedural complexity, required training, and lack of clinical trials [16, 17]. Intraoral scanners (IOSs) and intraoral cameras (IOCs) have emerged as promising alternatives, offering high-resolution, real-time imaging with digital analysis capabilities [18–20]. Previous studies have independently evaluated the diagnostic performance of IOCs [21–23] and IOSs [9, 20], compared to traditional methods, but reported SE and SP values vary considerably. These discrepancies may be attributed to differences in image projection geometry (2D vs. 3D), lesion characteristics, and examiner-related variables such as training and experience. Since SE (true positive rate) and SP (true negative rate) directly influence treatment decisions, imbalances between the metrics may lead to diagnostic misclassification, resulting in over- or undertreatment [14].

A recent systematic review explored the diagnostic applications of IOSs across various dental contexts [24]. However, to date, no systematic review and meta-analysis has systematically gathered and analyzed studies that exclusively compare the diagnostic accuracy of IOSs and IOCs against traditional methods for caries detection. Therefore, the present study aims to address this gap by synthesizing current evidence on the diagnostic performance of IOSs and IOCs, with particular attention to lesion type, lesion location, and examiner-dependent variability.

### Objectives

To compare the diagnostic accuracy of the following methods for detecting dental caries: (1) IOSs Vs radiography, (2) IOSs Vs histological analysis, (3) IOCs Vs radiography, (4) IOCs Vs histological analysis.

### Research questions

What is the diagnostic accuracy of IOSs and IOCs in detecting dental caries, as measured against histological and radiographic reference standards?

Additionally, how do these modalities compare to one another in terms of SE and SP across lesion types, locations, and examiner-dependent factors such as training and experience?

## Materials and methods

### Protocols

This systematic review followed the Preferred Reporting Items for Systematic Reviews and Meta-Analysis for Diagnostic Test Accuracy (PRISMA-DTA) checklist. The study was registered in the International Prospective Register of Systematic Reviews (PROSPERO; Registration No CRD42024547117).

### Eligibility criteria

The inclusion and exclusion criteria below were applied to screen and retrieve relevant data. Articles that fulfilled all inclusion criteria were selected for review.

#### Inclusion criteria

1. Randomized or non-randomized clinical trials, or case-control studies using 3D and 2D intraoral images of caries lesions (cavitated or non-cavitated) in deciduous or permanent human teeth
2. Studies with a minimum of 10 participants or extracted human teeth
3. Use of at least one globally recognized caries scoring system such as ICDAS, ICDAS-II, UniViSS, ICCMS, ADA, BASCD, and a defined numerical caries scoring system
4. Comparative evaluation of IOCs or IOSs against radiographic or histological reference methods and reported at least SE and SP for diagnostic performance. Reporting additional metrics such as Area Under Cover (AUC), and Positive Predictor Values (PPV), and Negative Predictor Values (NPV) was preferred, but not mandatory.
5. Studies using visual methods as comparative tools were included only if histological or radiographic methods were also used as the reference standard

#### Exclusion criteria

1. Non-human or artificial teeth, including synthetic or 3D-printed teeth, teeth with artificially induced carious lesions, and studies using radiographs as their primary study sample or teeth embedded in a cadaver.
2. Studies involving patients with systemic diseases or conditions that could affect caries presentation or diagnosis (e.g., cancer therapy, diabetes, neurological disorders), or dental anomalies (e.g., gingivitis, periodontitis, pulpal diseases, developmental dental defects), or patients undergoing orthodontic treatment.
3. Studies using cone beam computed tomography (CBCT), micro-computed tomography (M-CT), magnetic resonance imaging (MRI), or laser devices such as DIAGNODent Pen
4. Mixed diagnostic studies that combined eligible devices (IOCs or IOSs) with excluded technologies mentioned in exclusion criterion no 3
5. Studies without radiographic or histological methods as the comparative standard
6. Articles in non-English languages without translation, non-peer-reviewed papers, forensic studies, or patent/technology development reports unrelated to diagnostic accuracy.

### Information sources

Data were gathered from Scopus, Web of Science, PubMed, Cochrane Library, and Dentistry & Oral Sciences Source. The search was conducted between 18th July to 26th July 2024, with no restriction on publication year. The filter ‘Dentistry’ was applied in all databases.

### Search strategy

Customized keyword-based search strings using Boolean Logic and wild-card characters were used to query the databases [25]. Article search was conducted independently by 2 reviewers (FR & THF), and discrepancies were resolved through discussion. An online systematic review screening tool (*Covidence*) was used. A summary of the database-specific search strategies and article yields is provided in Supplementary Table 1.

### Selection process

The selection process followed the PICO model

#### Population

Carious lesions at various stages ranging from early demineralized to cavitated lesions, in deciduous or permanent human teeth, in either in-vivo or in-vitro studies.

#### Intervention

Intraoral imaging using IOSs or IOCs.

#### Comparison

Radiographic or histological assessments as the reference standards

#### Outcome

Primary outcomes included SE, SP, AUC, PPV, and NPV. The secondary outcomes included factors affecting diagnostic accuracy, such as factors related to study conditions and methodological heterogeneity, lesion characteristics, examiner-dependent variabilities, and diagnostic reproducibility (examiner training, experience, and inter/intra-rater reliability) and technical and device-dependent variables.

### Data collection process

*Covidence* was used for duplicate removal and screening. Titles and abstracts were screened based on the predefined inclusion and exclusion criteria. Full-text articles were reviewed for eligibility and compliance with the PICO model.

### Extracted data items and descriptions

The following data were extracted from each included studies: author name and year of publication, study design (in-vivo/in-vitro), sample size and characteristics (deciduous/permanent, maxillary/mandibular), participant details, teeth surface, intra oral imaging devices (type/brand), caries scoring system, comparison method, reference standard, diagnostic matrics, clinical significance, clinician’s or observers calibration and training in carious lesion evaluation, blinding of observers, inter and intra-rater reliability (if reported), device variabilities, confounding factors and study limitations.

### Study risk of bias assessment

The methodological quality and risk of bias of the included studies were assessed using the Joanna Briggs Institute (JBI) Critical Appraisal Diagnostic Accuracy Test (DTA) Checklist. Articles were rated high risk of bias (<50% positive responses), moderate risk (50–69%), or low risk (≥70%) [26].

To ensure consistency in assessment, one reviewer (FR) conducted the JBI appraisal for all selected articles. To reduce subjectivity, a subset of articles was cross-checked by two additional reviewers (JD and THF). As this involved 3 raters assigning categorical scores, Fleiss’s kappa (*k*) [27] was used to assess inter-rater reliability. Reviewer agreement was also summarized using a confusion matrix.

### Synthesis of meta-analysis result

#### Meta-analysis scoring criteria

Eligible studies were selected using a modified Newcastle-Ottawa Scale (NOS). Studies scoring 5 out of 13 were classified as having a moderate risk of bias (threshold range: 4–6) [28–30] and were deemed eligible for inclusion in this meta-analysis. The detailed scoring criteria, adapted for diagnostic accuracy studies, are provided in Supplementary Table 2.

#### Data extraction for meta-analysis and statistical analysis

From the selected studies, the following data were extracted: study identifiers, number of examiners, sample size, devices used, comparable tools, reference standards, lesion type (e.g., enamel, dentin, cemento-enamel junction, supragingival), and location, SE, and SP. When not directly available, true-positive (TP), false-positive (FP), true-negative (TN), and false-negative (FN) values were calculated using the equations adapted from Mainkar et al. [31] : 1. Caries-positive teeth**:** TP = SE × number of caries-positive teeth**;** FN = number of caries-positive teeth—TP 2. Caries-negative teeth**:** TN = SP × number of caries-negative teeth**;** FP = number of caries-negative teeth—TN.A bivariate random-effects model was used to estimate pooled SE and SP. This model accounts for both within-study sampling error and between-study heterogeneity, as well as the correlation between SE and SP across studies. It assumes that variability of test accuracy may arise from clinical, methodological, or population-related differences [32]. To stabilize variance and satisfy the model’s assumptions, SE and SP values were logit-transformed before analysis. Final pooled estimates were back-transformed to the proportion scale for interpretability. Between-study variance was quantified using the Tau² statistic, and the presence of residual heterogeneity was evaluated visually through forest plots and numerically using the I² statistic when appropriate.

To compare the diagnostic performance across methods, lesion types, and locations, Welch’s ANOVA analysis was used, followed by the Games-Howell post hoc tests to identify pairwise differences. Subgroup meta-analyses were also conducted to examine the influence of lesion characteristics and examiner-dependent variability on diagnostic accuracy. All statistical analyses were performed using the Python programming language (Version 3.10), executed in Google Colaboratory (Google Colab, Google Research, California).

### Ethics approval and consent to participate

This study is a systematic review and meta-analysis based exclusively on data from previously published articles in the field of dentistry, hence, as no new human or animal participants were involved, ethical approval and consent was not required.

## Results

### Literature identification

**Fig. 1:**
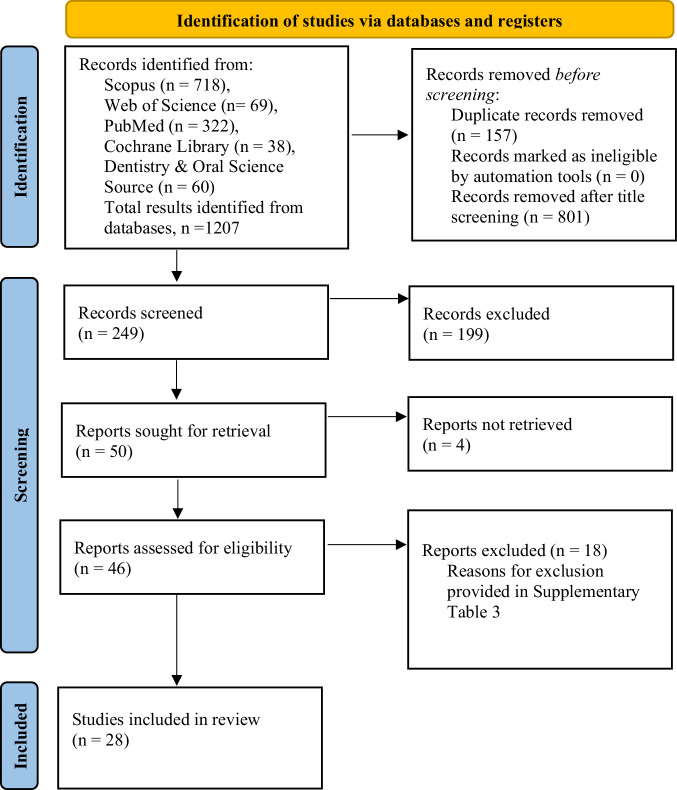
PRISMA flow diagram of the study selection process for systematic review.

One thousand two hundred and seven studies were identified across 5 databases: Scopus (*n* = 718), Web of Science (*n* = 69), PubMed (*n* = 322), Cochrane Library (*n* = 38), and Dentistry & Oral Sciences Source (*n* = 60). After abstract screening, 199 studies were excluded, leaving 50 articles for full review. Of these, 22 were excluded for reasons such as missing diagnostic metrics, non-imaging-based methods, lack of reference standards, or unavailable full texts. A detailed list of exclusions is provided in Supplementary Table 3. Finally, 28 studies met the inclusion criteria and were included in this systematic review. (Fig. 1 summarizes the identification, screening, eligibility assessment, and inclusion of studies for this systematic review).

### Study characteristics

The 28 included studies were published between 1998 and 2024. Of these, 6 were experimental studies, while 22 followed a comparative study design, which included one retrospective audit, 12 in-vivo investigations involving human participants, 13 in-vitro studies using only extracted teeth, and 2 studies employing both in-vivo and in-vitro methodologies.

Regarding the sample characteristics, 3 studies focused on primary teeth, while the remaining 25 examined permanent teeth. All studies evaluated posterior teeth: 17 assessed both molars and premolars, 6 focused exclusively on molars, and 3 on premolars. Nine studies specified that samples included teeth from both the upper and lower jaws, while 19 did not report jaw-specific details.

Fifteen studies involved human participants, with sample sizes ranging from 16 to 100 individuals and ages between 5 and 60 years. Nine studies reported male-to-female participant ratios, while the others provided only the total number of participants. Six studies specifically focused on pediatric participants, 3 included both children and adults, and the remaining studies examined only the adult population. Table 1 summarizes the included studies, highlighting designs, sample details, diagnostic thresholds, and key outcomes relevant to IOS and IOC performance, and Supplementary Table 4 details observer-related variability, device-specific issues, and methodological limitations that may have influenced diagnostic performance across studies.

**Table 1:** Summary of study characteristics, diagnostic thresholds, and performance metrics of intraoral scanners (IOSs) and intraoral cameras (IOCs) compared to reference methods (e.g., radiography, visual inspection).

Author (Y)	Study Characteristics (Design and Type)	Sample Characteristics	Intraoral Devices (Type and Brand)	Caries Scoring System	Reference Standard	Comparable tool	Diagnostic Thresholds	Summary of key findings	Examiner Calibration and Training	Clinical Significance
Patel et al. [43]	Comparative study (Retrospective Audit)	**Teeth Details** Primary teeth, *N* = 466 (129 maxillary first molar surfaces, 133 maxillary second molar surfaces, 121 mandibular first molar surfaces and 116 mandibular second molar surfaces); **Participant details** *N* = 44 (18 females and 26 males); age range = at least 7 years	**Intraoral camera** • Brand name DIAGNOcam 2170 U by KaVo **Digital radiography** • Brand name Heliodent Plus Intraoral X-ray unit by Sirona	International Caries Classification and Management System (ICCMS) and International Caries Detection and Assessment System (ICDAS-II)	Radiography	Visual and Radiography	The study used the following predefined thresholds for lesion scoring: 0 for sound surface, 1 for initial sign in enamel, 2 for established caries in enamel, and 3–5 for dentin involvement (outer, middle, and inner thirds)	• **ICDAS-II (Visual)** **Sound surface** SE = 95%, SP = 37%, Overall Accuracy = 74%, AUC = 0.69 **Enamel Lesions** SE = 2%, SP = 99%, Overall Accuracy = 78%, AUC = 0.69 **Dentin Lesions** SE = 62%, SP = 93%, Overall Accuracy = 88%, AUC = 0.69 • **Intraoral Camera (DIAGNOcam 2170)** **Sound surface** SE = 92%, SP = 57%, Overall Accuracy = 79%, AUC = 0.70 **Enamel Lesions** SE = 36%, SP = 88%, Overall Accuracy = 77%, AUC = 0.69 **Dentin Lesions** SE = 44%, SP = 98%, Overall Accuracy = 89%, AUC = 0.69	• Two examiners have performed the examination. • Both examiners have taken a workshop on DIAGNOcam, radiograph, and ICDAS-II scoring criteria. • Any disagreements were resolved by a third investigator.	Authors noted that DIAGNOcam showed high SP, especially for identifying sound and dentine surfaces, which makes it valuable for confirming the absence of caries and for non-invasive monitoring in children. However, due to its lower SE for detecting enamel lesions compared to the radiographic technique, DIAGNOcam is best used as an adjunctive tool rather than a replacement for radiography in proximal caries detection.
Cuenin et al. [6]	Comparative study (In-vivo)	**Teeth Details** *N* = 344 (289 permanent and 63 primary maxillary and mandibular molar teeth), **Participant details** *N* = 17, age range = 18 years or younger	**Intraoral Scanner** • Brand name iTero Element 5D scanner by Align Technology **Digital Radiography** • Brand name Not specified	International Caries Detection and Assessment System (ICDAS)	Radiography	Radiography	The study used the following thresholds, Scanner: Same grades as BW, focusing on light scattering patterns, Radiography (ICCMS): grade 0 (Sound), grade 1 (outer enamel radiolucency), grade 2 (radiolucency to the DEJ, grade 3 (outer dentin involvement) and grade 4 (middle dentin involvement)	• **Intraoral Scanner (iTero Element 5D)** SE = 23.08%, SP = 96.1%, PPV = 42.86%, NPV = 90.71%, Over all Accuracy = 87.79% • **Digital Radiograph (BWR)** SE = 24% (Early enamel lesion), 36% (larger lesions extending into dentin), and 60% (cavitated lesion), SP = 97%	• Five examiners participated in the study. All were general or pediatric dentists with 10 years of experience. • The examiners were calibrated using the clinical guide provided by Align Technology for the iTero Element 5D scanner. • Training involved reviewing example images and practicing lesion scoring based on the ICCMS (International Caries Classification and Management System) standards, followed by group discussions to address disagreements.	The study highlighted that the iTero Element 5D demonstrated high SP, making it highly reliable in identifying healthy tooth surfaces and reducing the risk of overtreatment. However, its low SE limits its effectiveness in detecting early carious lesions, which the authors emphasized as a critical consideration for clinical applications, especially for early-stage caries detection. Despite this limitation, the non-ionizing nature of the iTero Element 5D makes it ideal for frequent monitoring, particularly in pediatric populations where minimizing radiation exposure is essential. In contrast, BWR performed better in detecting larger, more advanced lesions extending into dentin, reinforcing their role as the standard diagnostic tool for interproximal caries detection.
Saffarpour et al. [7]	Comparative study (In-vitro)	**Teeth Details** Permanent teeth, *N* = 80 (all premolars) **Participant details** NA	**Intraoral camera** • Brand name VistaCam iX by Durr Dental **Digital Radiography** • Brand name Kodak 2200 Intraoral X-ray System by Kodak	International Caries Detection and Assessment System (ICDAS-II)	Histological	Visual and Radiography	The study used the following predefined fluorescence-based thresholds for VistaCam iX, 0–1 for healthy enamel, 1–1.5 for Initial caries, 1.5–2 for caries in the inner enamel layer, 2–2.5 for caries in the outer dentin layer and 2.5–3 for caries in the inner dentin layer.	• **ICDAS-II (Visual)** SE = 78.6%, SP = 100%, PPV = 100%, NPV = 93.9% • **Intraoral camera (VistaCam iX)** SE = 78.6%, SP = 42.4%, PPV = 28.4%, NPV = 86.7% • **Digital Radiography (BWR)** SE = 35.7%, SP = 95.7%, PPV = 71.4%, NPV = 83.0%	• A single specialized dentist conducted the visual inspections using direct vision, reflected light, and a three-in-one syringe. • Examiner training and calibration is unclear for visual and photographic methods.	ICDAS-II demonstrated the highest SP and PPV, effectively minimizing false positives and accurately diagnosing caries-free surfaces. The author suggested that ICDAS-II could be the preferred first-line method for occlusal caries detection in clinical settings due to its strong SE and SP. VistaCam showed high SE but low SP, which could lead to false positives, possibly misinterpreting enamel cracks as caries. This indicates that VistaCam may be better suited as an adjunct to visual inspection. For BWR, the authors acknowledged its utility but highlighted its lower SE, suggesting it may miss early carious lesions, thus making it less ideal as a sole diagnostic tool for initial caries detection.
Ntovas et al. [44]	Comparative study (In-vivo and In-vitro)	**Teeth Details** Permanent teeth, *N* = 53 (12 mandibular and 3 maxillary premolars, 18 mandibular and 25 maxillary molars), **Participant details** *N* = not specified, age range = 18–60 years	**Intraoral Scanner** • Brand name TRIOS 4 by 3Shape TRIOS A/S	International Caries Detection and Assessment System (ICDAS)	Histological	Visual	The study used the following predefined thresholds: ICDAS: 0 (sound) to 4 (shadow or cavitation indicating dentin involvement)	• **ICDAS (Visual)** **Inner half to outer half of Enamel** SE = 82%, SP = 59%, AUC = 0.76 and ACC = 0.79 **Outer third to inner third of Dentin** SE = 93%, SP = 75%, AUC = 0.90, ACC = 0.77 **Inner third of Dentin** SE = 100%, SP = 96% to 99%, AUC = 1.00 • **Intraoral Scanner (TRIOS 4)** **On screen tooth Color 3D model:** **Inner half to outer half of Enamel** SE = 75%, SP = 71%, AUC = 0.77, ACC = 0.74 **Outer third to inner third of Dentin** SE = 85%, SP = 83%, AUC = 0.90, ACC = 0.83 **Inner third of Dentin** SE = 100%, SP = 96% to 99%, AUC = 1.00 **On-Screen Color + Fluorescence 3D Models**: **Inner half to outer half of Enamel** SE = 74%, SP = 65%, AUC = 0.76, ACC = 0.72 **Outer third to inner third of Dentin** SE = 85%, SP = 80%, AUC = 0.91, ACC = 0.80 **Inner third of Dentin** SE = 100%, SP = 96% to 99%, AUC = 1.00	• A single examiner conducted both the visual and intraoral scanner examinations, while an independent examiner performed the histological analysis. • The examiner responsible for caries classification underwent training using ICDAS educational software. • Both examiners independently scored 10 teeth, followed by discussions to resolve any disagreements. • A second round of calibration was conducted using an additional 10 teeth to ensure consistency.	Both ICDAS and on-screen assessments using TRIOS 4 showed high SE for detecting extensive caries lesions, confirming their effectiveness in correctly identifying advanced caries. However, for early lesions, SE was slightly lower with TRIOS 4, likely due to its inability to replicate wet-dry transitions observed in clinical examinations, giving clinical visual methods a slight advantage. While both methods demonstrated high accuracy (up to 99%) for diagnosing deeper lesions, their accuracy SP for early lesions was moderate to good, indicating their utility but limited reliability for early-stage caries detection.
Kanar et al. [15]	Comparative study (In-vivo)	**Teeth Details** Permanent teeth, *N* = 639 (molars and premolars of maxillary or mandibular, no specific numbers is given) **Participant details** *N* = 22, age range = 14–45 years	**Intraoral Scanner** • Brand name iTero Element 5D scanner by Align Technology **Digital Radiography** • Brand name Panoramic Radiograph (PR) by Planmeca digital panoramic radiograph system and Bitewing Radiography (BWR) using Vistascan along with Phosphor plates by Dürr Dental	International Caries Detection and Assessment System (ICDAS)	Visual clinical examination and tactile confirmation	Visual and Radiography	The study used the following thresholds, Scanner: brightness patterns determined scores (0: sound, 1: early enamel lesion, 2: dentin involvement), Radiography (BWR/PR): Radiolucency patterns categorized lesions in line with the scanner scoring system.	• **ICDAS (Visual)** **Molar teeth** SE = 65.1%, SP = 63.6%, PPV = 87.5%, NPV = 31.8%, Overall Accuracy = 64.8% **Premolar teeth** SE = 65.1%, SP = 92.6%, PPV = 93.3%, NPV = 62.5%, Overall Accuracy = 75.7% • **Intraoral Scanner (iTero Element 5D)** **Molar teeth** SE = 88.4%, SP = 63.6%, PPV = 92.7%, NPV = 61.5%, Overall Accuracy = 85.2% **Premolar teeth** SE = 76.7%, SP = 59.3%, PPV = 75.0%, NPV = 61.5%, Overall Accuracy = 70.0% • **Digital Radiography** **BWR** **Molar teeth** SE = 90.7%, SP = 100%, PPV = 100%, NPV = 73.3%, Overall Accuracy = 92.6% **Premolar teeth** SE = 95.3%, SP = 96.3%, PPV = 97.6%, NPV = 92.9%, Overall Accuracy = 95.7% **PR** **Molar teeth** SE = 62.8%, SP = 100%, PPV = 100%, NPV = 40.7%, Overall Accuracy = 70.4% **Premolar teeth** SE = 46.5%, SP = 100%, PPV = 100%, NPV = 54.0%, Overall Accuracy = 67.1%	• Three examiners participated in the study; however, only the data from the primary examiner was used for analysis, with this examiner designated as the main observer. • The main examiner was calibrated using clinical guidelines provided by the manufacturer of the iTero Element 5D scanner. Sample cases were scored and reviewed in collaboration with senior faculty members to ensure consensus and standardization in interpretation.	The authors highlighted that while the intraoral scanner (iTero Element 5D) demonstrated acceptable SE and SP, its accuracy was relatively lower than BWR. The study reinforced BWR as the gold standard for detecting interproximal caries, particularly lesions involving the dentino-enamel junction, due to its superior SE, SP, and accuracy. Among all diagnostic methods compared, BWR achieved the highest validation match rates, confirming its reliability as the most effective tool for clinical use. In contrast, PR exhibited significantly lower SE for detecting early enamel lesions, especially in premolars, which the authors attributed to limitations such as superposition of structures and lower image resolution. Consequently, the study suggests that PR has a restricted role in the early detection of interproximal caries. The PPV and NPV for each method informed the reliability of diagnostic outcomes in clinical practice. BWR’s high PPV and NPV were cited as critical to its clinical utility, while intraoral scanner showed potential for adjunctive use, especially when radiation is a concern.
Edrees et al. [36]	Comparative study (In-vivo)	**Teeth Details** Permanent teeth, *N* = 102 (60 maxillary and 42 mandibular), **Participant details** *N* = 36 (17 males and 19 females), mean age 27.75 ± 6.16 years	**Intraoral Camera** • Brand name VistaCam iX Proxi HD by Durr Dental **Digital Radiography** • Brand name Heliodent DS intraoral X-ray machine with phosphor storage plates by Sirona	Caries Detection and Assessment System (ICDAS-II) and International Caries Classification and Management System (ICCMS)	Visual and Radiography	Visual and Radiography	The study used the following thresholds, ICDAS-II: 0 (sound) to 4 (deep dentinal shadow). Radiography (ICCMS): R0 (no radiolucency) to R4 (radiolucency in the middle third of dentin). VistaCam: NIR0 (no visible changes), NIR1 (bright structures in enamel) and NIR2 (bright structures reaching or crossing the DEJ)	• **VistaCam iX Proxi HD Vs. ICDAS (Visual reference)** **Enamel Lesions** SE = 97.9%, SP = 50%, AUC = 0.74, PPV = 98%, NPV = 50%, Overall Accuracy = 96.1% **Dentin Lesions** SE = 84.3%, SP = 100%, AUC = 0.96, PPV = 100%, NPV = 20%, Overall Accuracy = 83% • **VistaCam iX Proxi HD Vs. Digital Radiography (BWR) (Radiographic reference)** **Enamel Lesions** SE = 100%, SP = 40%, AUC = 0.70, PPV = 93.9%, NPV = 100%, Overall Accuracy = 88.2% **Dentin Lesions** SE = 84.3%, SP = 100%, AUC = 0.95, PPV = 100%, NPV = 42.9%, Overall Accuracy = 80%	• Two experienced examiners conducted the assessments independently. • Examiner calibration sessions were held 2 weeks before the study, during which the examiners examined 60 teeth using the three diagnostic methods.	VistaCam iX Proxi HD demonstrated very high SE in detecting enamel lesions, making it clinically significant for identifying early-stage, non-cavitated caries. However, its low SP for enamel lesions, attributed to factors such as enamel thickness, surface curvature, or artifacts like stains, may lead to overestimation of caries presence. For dentin lesions, VistaCam iX Proxi HD exhibited both high SE and SP, particularly when compared to BWR, underscoring its reliability for diagnosing advanced caries that require restorative intervention. The AUC values, while slightly lower for enamel lesions, were very high for dentin lesions, emphasizing the reliability of VistaCam iX Proxi HD for detecting deeper lesions. Clinically, this highlights its potential as a non-invasive tool for diagnosing early and advanced caries.
Salama et al. [35]	Comparative study (In-vivo)	**Teeth Details** Permanent teeth, *N* = 94 (45 maxillary molars and 49 mandibular molars); **Participant Details** *N* = 34 (15 females and 19 males); age range = at least 18 years	**Intraoral camera** • Brand name Vista Proof iX HD Smart	International Caries Detection and Assessment System (ICDAS-II)	Visual and Histological	Visual and Histological	The study used manufacturer-defined thresholds for VistaProof camera, <2 for enamel caries, 2–2.5 for dentin caries at the dentinoenamel junction, and >2.5 for dentin caries penetrating deeper layers.	• **Intraoral camera** 1. **Based on ICDAS-II as Reference** **Enamel Lesions** SE = 48%, SP = 100%, PPV = 100%, NPV = 53%, Overall Accuracy = 67%, AUC = 0.11 **Dentin Lesions** SE = 100%, SP = 48%, PPV = 53%, NPV = 100%, Overall Accuracy = 67%, AUC = 0.88 2. **Based on Fissurotomy as Reference** **Dentin Lesions** SE = 95%, SP = 0%, Overall Accuracy = 95%, AUC = 0.81	• Two examiners conducted both the ICDAS-II and VistaProof HD assessments • 15 days before the main assessments, both examiners practiced on 60 extracted teeth for calibration. • Which method they have practiced is unclear.	The authors noted that Vista Proof iX HD Smart showed a high SE and strong agreement with histological validation, supporting its potential for early and accurate detection of deeper lesions. However, the device’s lower SP for enamel lesions suggests caution, as it may lead to false positives at this stage. This suggests that Vista Proof may be more effective as a complementary tool than a standalone diagnostic method.
Wang et al. [21]	Comparative study (In-vivo)	**Teeth Details** Permanent teeth, *N* = 118 (all premolars); **Participant details** *N* = 70 (33 females and 37 males); age range = 12–44	**Intraoral camera** • Brand name DIAGNOcam by KaVo **Digital radiography** • Brand name Planmeca X-ray unit and Kwik-Bite Senso aiming device	International Caries Detection and Assessment System (ICDAS-II)	Histological	Visual and Radiography	The study applied the following thresholds for DIAGNOcam, 0 for now shadow, 1 for shadow in the outer half of enamel, 2 for shadow in the inner half of the enamel, 3 shadow at the EDJ, 4 for shadow in dentine	• **ICDAS-II (Visual)** **Outer Enamel** SE = 33%, SP = 94%, AUC = 0.63 **Inner Enamel** SE = 46%, SP = 98%, AUC = 0.72 **Dentine** SE = 52%, SP = 100%, AUC = 0.76 • **Intraoral Camera (DIAGNOcam)** **Outer Enamel** SE = 68%, SP = 94%, AUC = 0.81 **Inner Enamel** SE = 75%, SP = 97%, AUC = 0.86 **Dentine** SE = 84%, SP = 98%, AUC = 0.91 • **Digital Radiography (BWR)** **Outer Enamel** SE = 47%, SP = 94%, AUC = 0.70 **Inner Enamel** SE = 54%, SP = 98%, AUC = 0.77 **Dentine** SE = 88%, SP = 99%, AUC = 0.94	• A single examiner performed all three methods • The examiner underwent ICDAS-II training on the official ICDAS training platform, performed radiographs under the supervision of an experienced dentist, and learned the proper use of the DIAGNOcam through guidance based on clinical experience and the recommendations from a relevant article.	The authors highlighted that DIAGNOcam demonstrated higher SE than both ICDAS-II and BWR) for detecting early enamel lesions, suggesting that DIAGNOcam may be more effective for early diagnosis of proximal caries. However, the authors noted that for detecting deeper dentine lesions, radiography showed the highest SE and AUC, making it more reliable for advanced lesions.
Valizadeh et al. [37]	Comparative study (In-vitro)	**Teeth details** Permanent teeth, *N* = 40 (17 first molars and 23 premolars) **Participant details** NA	**Intraoral camera** • Brand name VistaCam IX Proxi by Durr Dental **Digital radiography** • Brand name GENDEX intraoral radiography unit with photostimulable phosphor plates by GENDEX	A numerical caries scoring system was used. The exact name is not specified	Histological	Radiography	The study used the following predefined thresholds for VistaCam IX Proxi to determine the lesions severity, 0 for sound surface, 1 for caries in enamel, 2 for caries in the external half of dentin and 3 for caries in the internal half of dentin.	• **Intraoral Camera (VistaCam IX Proxi)** **Enamel** SE = 100%, SP = 71.4%, AUC = 0.72, PPV = 71.4% **External half-dentine caries** SE = 72.7%, SP = 71.4%, AUC = 0.72, PPV = 66.6% **Internal half-dentine caries** SE = 82.3%, SP = 71.4%, AUC = 0.72, PPV = 93.3% • **Digital Radiography** **Enamel** SE = 40%, SP = 87.6%, AUC = 0.45, PPV = 40% **External half-dentine caries** SE = 54.5%, SP = 87.6%, AUC = 0.45, PPV = 50% **Internal half-dentine caries** SE = 58.8%, SP = 87.6%, AUC = 0.45, PPV = 83.3	• Two oral and maxillofacial radiologists independently evaluated the images. • Examiners were briefed on the correct interpretation of the images, enhancement techniques, and scoring criteria before the evaluations. • The same images were evaluated after 2 weeks to ensure consistency.	VistaCam IX Proxi exhibited higher SE, particularly for detecting enamel and early dentin caries, making it more effective for identifying reversible incipient caries with preventive measures. The non-invasive and radiation-free nature of VistaCam IX Proxi makes it especially suitable for pediatric patients, high-risk individuals, and cases where radiography is contraindicated. Digital radiography demonstrated higher SP, reducing the likelihood of false positives, which is beneficial for avoiding unnecessary treatments. However, its lower SE, especially for enamel caries, limits its effectiveness in detecting early caries.
Mokhtar et al. [39]	Comparative study (In-vivo)	**Teeth Details** Permanent teeth, *N* = 52 (44 molars and 8 premolars), **Participant details**, *N* = 16 (8 males and 8 females), age range = 7 to 15 years	**Intraoral camera** • Brand name DIAGNOcam by Kavo **Digital radiography** • Brand name CMOS sensor by EzSensor with a Kwik-bite holder by Kerr United Kingdom, coupled with the Satelec X-Mind AC/DC machine by Satelec ACTEON Tuusula	International Caries Detection and Assessment System (ICDAS)	Visual (Clinical validation after examining the lesion post-operatively to confirm the presence and depth of caries)	Radiography	The study used the following thresholds, 0 (sound surface) to 4 (underlying dentin shadow) for ICDAS and 0 (normal shadow) to 4 (broad shadow indicating groove-fossa involvement and dentin perforation for DIAGNOcam	• **ICDAS (Visual)** **Enamel** SE = 88%, SP = 89%, AUC = 0.90 **Dentine** SE = 89%, SP = 88%, AUC = 0.90 • **Intraoral Camera (DIAGNOcam)** **Enamel** SE = 83%, SP = 93%, AUC = 0.93 **Dentine** SE = 93%, SP = 83%, AUC = 0.93 • **Digital Radiography** **Enamel** SE = 83%, SP = 61%, AUC = 0.73 **Dentine** SE = 61%, SP = 83%, AUC = 0.73 • **Combined ICDAS and DIAGNOcam** **Enamel** SE = 92%, SP = 93% **Dentine** SE = 93%, SP = 92%, AUC = 0.98 (Combined model)	• Two experienced examiners conducted the procedures. • Examiners were calibrated using a training set of 30 extracted teeth, where they practiced scoring using ICDAS, radiographs, and intraoral camera imaging.	The study emphasized the clinical significance of combining Visual Inspection (ICDAS) and DIAGNOcam, as this approach demonstrated the highest SE and SP for detecting enamel and dentine caries. The combined diagnostic model significantly reduced the likelihood of missed lesions or false positives, making it ideal for early caries detection, preventive care, and monitoring lesion progression over time.
Michou et al. [41]	Comparative study (In-vitro)	**Teeth Details** Permanent teeth, *N* = 95 (66 molars and 29 premolars) **Participant details** NA	**Intraoral Scanner** • Brand name TRIOS 4 by 3Shape TRIOS A/S **Intraoral Camera** • Brand name DIAGNOcam by Kavo **Digital Radiography** • Brand name SOREDEX MINRAY X-ray device by Durr Dental	International Caries Detection and Assessment System (ICDAS II)	Histological	Visual, Intraoral camera, and Radiography	The study used the following thresholds, ICDAS: E0 (sound) to 6 (Sever cavitation), Scanner: NIR0 (sound) to NIR4 (dentin involvement), Histology: E0 (sound) to D2/D3 (deep dentin lesions),	• **ICDAS-II (Visual)** **Enamel Lesion (E1–E2)** SE = 89%, SP = 75%, AUC = 0.92 **Dentine Lesions (D1–D3)**: SE = 93%, SP = 89%, AUC: 0.95 • **Intraoral Scanner (TRIOS 4)** **Enamel Lesions (E1–E2)** SE = 86%, SP = 78%, AUC = 0.90 **Dentine Lesions (D1–D3)** SE = 88%, SP = 91%, AUC = 0.94 • **Intraoral Camera (DIAGNOcam)** **Enamel Lesions (E1–E2)** SE = 81%, SP = 72%, AUC = 0.86 **Dentine Lesions (D1–D3)** SE = 85%, SP = 87%, AUC = 0.91 • **Digital Radiography** **Enamel Lesions (E1–E2)** SE = 67%, SP = 84%, AUC = 0.78 **Dentine Lesions (D1–D3)** SE = 79%, SP = 92%, AUC = 0.88	• One examiner conducted the visual examination, another carried out the radiographic examination, and a third handled the assessments using the DIAGNOcam and TRIOS 4 intraoral scanner. • Examiners were trained and calibrated before the study. The training involved using the ICDAS system for visual and radiographic examinations and scoring a set of 20 teeth independently.	Prototype TRIOS 4, the high SE and AUC values demonstrate its effectiveness in detecting early enamel and advanced dentin caries lesions. This makes it a promising non-invasive diagnostic tool, especially in cases where minimizing radiographic exposure is necessary, such as in children or pregnant patients. The DIAGNOcam showed moderate SE and SP, with AUC values. It performed well for detecting advanced caries but had lower SP for early enamel lesions, indicating its strength lies in diagnosing deeper lesions rather than early-stage caries. With the highest SE and AUC values among the methods, ICDAS-II remains a clinically valuable tool, particularly when conducted by trained practitioners. However, the slightly lower SP for early lesions highlights a potential risk for over-diagnosis. Radiographic examination demonstrated lower SE for detecting enamel lesions, indicating it may not be reliable for early caries detection. However, it showed good SP and AUC for dentin lesions, highlighting its effectiveness in confirming advanced caries. As a result, radiographs are best utilized as a complementary diagnostic tool alongside methods like intraoral scanners or visual inspection to provide a more comprehensive assessment.
Alrayyes et al. [22]	Comparative study (In-vivo)	**Teeth Details** Primary teeth, *N* = 90 (all molars from both maxillary and mandibular), **Participant details**, *N* = 22 (10 males and 12 females), mean age range 6–11 years	**Intraoral camera** • Brand name CariVu by DEXIS **Digital Radiography** • Brand name Kavo Focus with Platinum Sensor by DEXIS or Gendex GXS-700 Sensor by Gendex Dental Systems, Kavo	International Caries Detection and Assessment System (ICDAS)	Visual (Clinical validation through visual-tactile examination after temporary tooth separation)	Radiography	The study used the following thresholds: no caries for no visible demineralization, incipient caries for lesions restricted to enamel, not reaching the DEJ and dentinal caries for lesions extending beyond the DEJ into dentin. No specific numerical values are provided	• **Intraoral Camera (DIAGNOcam)** **Any caries (Incipient + Dentinal)** SE = 72%, SP = 54% **Incipient caries** SE = 60%, SP = 53% **Dentinal caries** SE = 82%, SP = 53% • **Digital Radiography** **Enamel** **Any caries (Incipient + Dentinal)** SE = 82%, SP = 87% **Incipient caries** SE = 98%, SP = 86% **Dentinal caries** SE = 99%, SP = 87%	• The study involved 24 raters, including 9 first-year pediatric dentistry residents, 8 second-year pediatric dentistry residents, and 7 pediatric dentistry faculty members • All raters received prior theoretical and practical training on using the CariVu device from a commercial representative. • Two trained examiners independently performed the clinical validation by conducting direct visual-tactile examinations after temporary tooth separation • Both examiners were trained on the proper use of the devices.	While CariVu showed lower SE and SP for detecting incipient lesions, its performance for dentinal caries detection was comparable, suggesting its utility in identifying more advanced lesions. The authors emphasized the potential of CariVu as an adjunctive diagnostic tool, particularly in cases where radiography may not be feasible or preferred, noting its ability to enhance patient comfort and improve compliance in pediatric and anxious populations.
Michou et al. [41]	Comparative study (In-vivo and In-vitro)	**Teeth Details** Permanent teeth, *N* = 53 (molars and premolars, no specific numbers), **Participant details**, *N* = exact number not provided, age range = 18 to 60 years	**Intraoral Scanner** • Brand name TRIOS 4 by 3Shape TRIOS A/S	International Caries Detection and Assessment System (ICDAS)	Histological	Visual	The study used predefined thresholds for the 3D IOS system, ALG1/ALG2 for enamel (≥E1) and dentin lesions (≥D1) and ALG3/ALG4 for enamel (≥E1), dentin (≥D1), and deep dentin (≥D2)	• **ICDAS-II (visual)** SE = 90%, SP = 82%, AUC = 0.93 • **Intraoral Scanner (TRIOS 4)** **Algorithm 1** SE = 81%, SP = 79%, AUC = 0.88 **Algorithm 2** SE = 85%, SP = 83%, AUC = 0.92 **Algorithm 3** SE = 78, SP = 84%, AUC = 0.89 **Algorithm 4** SE = 87%, SP = 81%, AUC = 0.91	• A postgraduate student in restorative dentistry with at least 5 years of experience (Examiner 1) conducted the ICDAS examination, selected examination sites, and operated the 3D intraoral scanner for both in-vivo and in-vitro assessments. • The visual examiner was trained using ICDAS educational software and independently scored 20 teeth in 2-week intervals. • A Ph.D. student with 4 years of research experience in Cariology (Examiner 2) conducted the histological analysis and extracted the caries classification scores from the four automated algorithms	The automated caries detection algorithms showed high SE and SP with AUC values comparable to visual examination (ICDAS-II). The high SE of the algorithms makes them suitable for detecting caries lesions in early stages, supporting preventive care and reducing the need for more invasive treatments.
Metzger et al. [10]	Comparative study (In-vivo)	**Teeth Details** Permanent teeth, *N* = 1765 (molars and premolars from both maxillary and mandibular arches, no specific numbers are provided), **Participant Details**, *N* = 100 (46 males and 54 females), age range = 14 and older for Canadian cohort and 18 and older years for German cohort	**Intraoral Scanner** • Brand name iTero Element 5D scanner by Align Technology **Digital Radiography** • Brand name Not specified	American Dental Association (ADA) Caries Classification System	Clinical observations during caries excavation in 59 cases	Radiograph	The study used the following thresholds, Scanner: Early enamel lesions (triangular shape, no DEJ involvement) and DEJ involvement (trapezoid shape), Radiography (ADA Guidelines): Lesions categorized as early enamel or extending into dentin (DEJ involvement)	• **Intraoral Scanner (iTero Element 5D)** **Early Enamel Lesions** SE = 73.0%, SP = 96.8%, Overall Accuracy = 96% (Expert Team Results) **Dentin-Enamel Junction (DEJ) Lesions** SE = 88.5%, SP = 99.6%, Overall Accuracy = 99% (Expert Team Results) **Clinical Observations** **Lesions Limited to Enamel** SE = 97% **Lesions Reaching Dentin** SE = 96% • **Digital Radiograph** **Early Enamel Lesions** SE = 51.6%, SP = 90.4%, Overall Accuracy = 88% (Dentist-Reported Results) **Dentin-Enamel Junction (DEJ) Lesions** SE = 84.8%, SP = 97.1%, Overall Accuracy = 96% (Dentist-Reported Results) **Clinical Observations** **Lesions Limited to Enamel** SE = 14% **Lesions Reaching Dentin** SE = 54%	• Five dentists collected data using the intraoral scanner and radiographs in their respective clinical settings. • The dentists received online training on the use and interpretation of results from the iTero Element 5D scanner. • An expert team of five dentists, each with 2 years of experience, reviewed the intraoral scanner images and radiographs after receiving training and calibration from the sponsor, Align Technology.	The study highlighted that the iTero Element 5D demonstrated significantly higher SE (97% for enamel lesions) compared to digital radiograph (14%). This suggests that iTero Element 5D is more effective in detecting early-stage caries, which can contribute to better preventive care and early intervention of caries detection. Intraoral scanners and digital radiography showed comparable SE for detecting lesions at the dentino-enamel junction (DEJ), indicating that scanner is not inferior to radiography for diagnosing more advanced carious lesion. iTero Element 5D maintained superior SP for early enamel lesions compared to radiography. This indicates that the scanner is effective at minimizing false positives, making it a reliable diagnostic tool for confirming caries-free surfaces.
Stratigaki et al. [8]	Comparative study (In-vivo)	**Teeth Details** Permanent teeth, *N* = 116(14 first premolars, 52 s premolars, 40 first molars, 9 s molars, and 1 third molar); **Participant details**, *N* = 73 (43 females and 30 males); age range = 15–37 years	**Intraoral camera** • Brand name DIAGNOcam by Kavo **Digital Radiography** • Brand name Heliodent DS and HDX by Dental EZ	International Caries Detection and Assessment System (ICDAS-II) and Universal Visual Scoring System (UniViSS)	Composite reference standard	Radiograph	The study used the composite reference standard based on radiographic (D0-D3) and visual inspection (ICDAS/UniViSS) categories, with no specific numerical values	• **Intraoral camera (DIAGNOcam)** **Enamel** SE = 92%, SP = 39%, AUC = 0.65 **Inner Enamel** SE = 100%, SP = 42%, AUC = 0.78 **Dentin** SE = 100%, SP = 99%, AUC = 0.99 • **Digital Radiography (BWR)** **Enamel** SE = 81%, SP = 65%, AUC = 0.73 **Inner Enamel** SE = 74%, SP = 88%, AUC = 0.81 **Dentin** SE = 100%, SP = 100%, AUC = 1.00	• Three trained examiners (dentists) conducted the assessments. • The examiners attended a 2-day training session on DIAGNOcam which included theoretical and practical segments where they practiced on patients. • Examiners carried out the procedure twice.	DIAGNOcam demonstrated high SE, particularly for detecting inner enamel and dentine lesions, which could support in identifying early-stage caries that might be missed by visual inspection alone. However, DIAGNOcam’s lower SP compared to BWR, indicating that it could result in more false positives, potentially leading to unnecessary follow-up or overtreatment if used alone for treatment decisions
Michou et al. [40]	Experimental Study (In-vitro)	**Teeth Details** Permanent teeth, *N* = 70 (63 molars and 7 premolars) **Participant details** NA	**Intraoral Scanner** • Brand name TRIOS 3 by 3Shape TRIOS A/S **Digital Radiography** • Brand name Planmeca ProX radiographic system by Planmeca	International Caries Detection and Assessment System (ICDAS)	Histological	Visual and Radiography	The study used the following thresholds, Histology: E0 (sound) to D3 (inner third of dentin), ICDAS: 0 (sound) to 6 (extensive dentin lesions), and scanner functions (f1–f4, mathematical functions quantified fluorescence signal changes to define thresholds for each caries stage)	• **ICDAS (Visual)** **Enamel** SE = 93%, SP = 88%, AUC = 0.93 (Outer half of enamel, E1), SE = 78%, SP = 95%, AUC = 0.95 (Inner half of the enamel, E2) **Dentin** SE = 59%, SP = 98%, AUC = 0.97 (Outer third of dentin D1), SE = 60%, SP = 93%, AUC = 0.79 (Middle third of dentin D2), SE = 64%, SP = 73%, AUC = 0.73 (Inner third of dentin D3) • **Intraoral Scanner (TRIOS 3)** **Function f1** **Enamel** SE = 71%, SP = 100%, AUC = 0.87 (E1), SE = 66%, SP = 64%, AUC = 0.63 (E2) **Dentin** SE = 26%, SP = 86%, AUC = 0.46 (D1), SE = 84%, SP = 100%, AUC = 0.348 (D2), SE = 73%, SP = 80%, AUC = 0.39 (D3) **Function f2** **Enamel** SE = 89%, SP = 84%, AUC = 0.93 (E1), SE = 73%, SP = 80%, AUC = 0.83 (E2) **Dentin** SE = 84%, SP = 85%, AUC = (D1), SE = 84%, SP = 100%, AUC = 0.89 (D2), SE = 73%, SP = 80%, AUC = 0.82 (D3) **Function f3:** **Enamel** SE = 100%, SP = 84%, AUC = 0.94 (E1), SE = 86%, SP = 72%, AUC = 0.84 (E2) **Dentin** SE = 82%, SP = 85%, AUC = 0.88 (D1), SE = 84%, SP = 100%, AUC = 0.86 (D2), SE = 78%, SP = 82%, AUC = 0.80 (D3) **Function f4** **Enamel** SE = 91%, SP = 77%, AUC = 0.93 (E1), SE = 81%, SP = 93%, AUC = 0.85 (E2) **Dentin** SE = 91%, SP = 85%, AUC = 0.91 (D1), SE = 84%, SP = 100%, AUC = 0.93 (D2), SE = 78%, SP = 82%, AUC = 0.85 (D3) • **Digital Radiography** • **Enamel** SE = 84%, SP = 94%, AUC = 0.95 (E1), SE = 48%, SP = 100%, AUC = 0.94 (E2) **Dentin** SE = 58%, SP = 100%, AUC = 0.95 (D1), SE = 58%, SP = 100%, AUC = 0.94 (D2), SE = 64%, SP = 73%, AUC = 0.73 (D3)	• One main examiner conducted the assessments using the intraoral scanner and performed the histological analysis. • Two other independent examiners with 10 and 30 years of experience conducted the visual and radiographic assessments. • The main investigator was trained and calibrated for the histological assessment by an experienced researcher and for using the intraoral scanner by another expert.	TRIOS 3’s fluorescence-based functions showed high SE and good AUC values for detecting enamel lesions. This indicates the potential of the scanner for early caries detection, which is critical for preventive care. SP values for dentin lesions were generally high (up to 100% for f4 at some levels), reflecting the scanner’s ability to minimize false positives in detecting more advanced lesions. This improves diagnostic confidence when intervention decisions are required. The high AUC values across different caries stages suggest the system’s reliability in discriminating caries severity compared to the visual-tactile and radiographic methods.
Alamoudi et al. [23]	Experimental Study (In-vivo)	**Teeth Details** Primary teeth, *N* = 236 (all molars); **Participant details**, *N* = 66; age range = 5–8 years	**Intraoral camera** • Brand name DIAGNOcam 2170 by Kavo **Digital radiography** • Brand name FireCR PSP system with imaging plates by FireCR, 3DISC, a PSP X-ray holder, and an XCP centering device by DENTSPLY RINN. The X-ray source was Orix 70 by ARDET Dental and Medical Devices	International Caries Detection and Assessment System (ICDAS-II)	Visual	Radiography	The study used the predefined thresholds for DIAGNOcam, 0 for sound, 1 for first visible enamel lesion, 2 for established enamel lesion, 3 for lesion at the EDJ, 4 for dentin lesion and 5 for deep dentin lesion.	• **Intraoral camera (DIAGNOcam)** SE = 85.2%, SP = 56.9%, AUC = 72.2% • **Digital Radiography (BWR)** SE = 51.9%, SP = 57.9%, AUC = 56.1%	• Three trained and calibrated examiners performed the tests • All of them independently scored a set of 20 proximal carious lesions by using all three methods. • The same lesions were evaluated after 1 week to ensure consistent scoring.	The authors highlighted that the DIAGNOcam, with its higher SE and diagnostic accuracy, is a superior, non-invasive, and radiation-free alternative to BWR for detecting cavitated proximal carious lesions in primary molars. The author recommended using the DIAGNOcam for treatment decisions involving dentin lesions while advising that BWR alone are insufficient and should be complemented by visual examination with tooth separation when the DIAGNOcam is unavailable.
Tonkaboni et al. [19]	Experimental study (In-vitro)	**Teeth Details** Permanent teeth, *N* = 108 (molars and premolars) **Participant details** NA	**Intraoral camera** • Brand name VistaCam iX by Durr Dental **Digital Radiography** • Brand name Kodak 2200 Intraoral X-ray System by Kodak	International Caries Detection and Assessment System (ICDAS-II)	Histological	Visual and Radiography	The study applied manufacturer-defined thresholds for VistaCam Proxi: IR0 - No enamel changes, IR1 - Lesion extends to the dentinoenamel junction, IR2 - Lesion passes the dentinoenamel junction.	• **ICDAS-II (Visual)** SE = 63%, SP = 88%, AUC = 0.76 • **Intraoral camera (VistaCam iX)** SE = 74%, SP = 82%, AUC = 0.78 • **Digital Radiography (BWR)** SE = 68%, SP = 87%, AUC = 0.79	• Two examiners conducted all 3 methods. • The examiners were trained by assessing a set of teeth for visual inspection.	VistaCam iX demonstrated higher SE compared to BWR and visual inspection, which could make it valuable for early detection of proximal caries. However, while VistaCam iX had improved SE, BWR remained more specific, making it valuable for confirming diagnoses and avoiding over-treatment. This balance of SE and SP among methods suggests that combining these diagnostic tools could enhance clinical decision-making.
Iranzo-Cortes et al. [38]	Comparative study (in-vitro)	**Teeth Details** Permanent teeth, *N* = 65 (Not mentioned) **Participant details** NA	**Intraoral camera** • Brand name Vista Proof by Durr Dental	International Caries Detection and Assessment System (ICDAS-II)	Histological	Visual	The study used manufacturer-defined thresholds, 0–0.99 for sound enamel, 1–1.49 for initial caries, 1.5–1.99 for caries in inner enamel, 2–2.49 for caries in outer dentin, and 2.5 or higher for caries in inner dentin.	• **ICDAS-II (Visual)** SE = 79.6% (Examiner 1) and 86.4% (Examiner 2), SP = 81.0% (Examiner 1) and 95.2% (Examiner 2), PPV = 89.7% (Examiner 1) and 97.4% (Examiner 2), NPV = 65.4% (Examiner 1) and 76.9% (Examiner 2), AUC = 80.2% (Examiner 1) and 90.8% (Examiner 2) • **Intraoral Camera (VistaProof)** SE = 70.5% (Examiner 1) and 81.8% (Examiner 2), SP = 81.0% (Examiner 1) and 61.9% (Examiner 2), PPV = 88.6% (Examiner 1) and 81.8% (Examiner 2), NPV = 56.7% (Examiner 1) and 61.9% (Examiner 2), AUC = 75.7% (Examiner 1) and 71.9% (Examiner 2) • **Combined (ICDAS-II + VistaProof)** SE = 84.1% (Examiner 1) and 95.5% (Examiner 2), SP = 71.4% (Examiner 1) and 61.9% (Examiner 2), PPV = 86.1% (Examiner 1) and 84.0% (Examiner 2), NPV = 68.2% (Examiner 1) and 86.7% (Examiner 2), AUC = 77.8% (Examiner 1) and 78.7% (Examiner 2)	• Two examiners conducted the assessments, one was a 5th-year dental student, and the other was an experienced clinician trained in ICDAS-II • Both observers assessed a of 35 teeth (not included in the main sample) to establish consistent scoring for ICDAS-II. • Additionally, the examiners followed the manufacturer’s instructions during training for the VistaProof camera with the same set of 35 teeth.	The authors suggested that combining the two methods (ICDAS-II + VistaProof) enhances SE, making it easier to detect incipient lesions early. Although combining the methods slightly reduced SP (leading to some false positives), this was clinically acceptable since non-cavitated, early lesions can be managed conservatively without invasive treatment.
Elhennawy et al. [11]	Comparative study (in-vitro)	**Teeth Details** Permanent teeth, *N* = 200 (94 molars and 106 premolars) **Participant details** NA	**Intraoral Camera** • Brand name DIAGNOcam by Kavo **Digital Radiography** • Brand name Heliodent Plus by Sirona with a digital sensor by XIOS XG, Sirona	Two-stage modified caries scoring system was used	Visual and Radiography	Visual and Radiography	The study used the following thresholds, Radiography: 0 (no lesion), 1 (lesion confined to enamel), and 2 (lesion extending into dentin)	• **Visual** SE = 56.4%, SP = 90.3%, AUC = 0.73, PPV = 84.6%, • **Intraoral Camera (DIAGNOcam)** SE = 88.4%, SP = 82.5%, AUC = 0.86, PPV = 83.3%, NPV = 87.8% • **Digital Radiography (BWR)** SE = 73.5%, SP = 89.5%, AUC = 0.83, PPV = 88%, NPV = 76.5%	• Two experienced dentists participated in the study • Before the study, the examiners underwent calibration sessions where they assessed a set of test samples. Any disagreements were resolved through discussions	The authors emphasized the high sensitivity of DIAGNOcam for detecting both enamel and dentin lesions, highlighting its value in early caries detection. The high AUC values further validated its reliability and accuracy across different lesion stages, establishing it as a non-invasive, radiation-free alternative to traditional diagnostic methods. BWR on the other hand, achieved a strong balance between SE and SP, particularly for detecting advanced dentin lesions. This confirms its reliability for identifying lesions requiring restorative intervention, although its reliance on ionizing radiation remains a clinical limitation. The visual method demonstrated high SP but lacked SE, limiting its effectiveness for detecting early carious lesions. The authors highlighted that visual alone is insufficient for identifying early enamel lesions, emphasizing the importance of integrating adjunctive imaging techniques to enhance diagnostic accuracy.
Baltacioglu et al. [12]	Comparative study (In-vivo)	**Participant details** Permanent teeth, *N* = 52 (molars and premolars), **Participant Details**, *N* = 26 (9 males and 17 females), mean age range 30.25 years	**Intraoral camera** • Brand name DIAGNOcam by Kavo **Digital Radiography** • Brand name PSP-Bitewing radiography with Digora Optime system and XPP-DS digital sensor holder	Used five-point scale for caries detection	Visual (Clinical validation through direct visual-tactile examination after tooth separation)	Radiography	The study used the following predefined 5-point scoring system, 1 for definitely caries, 2 for probably caries, 3: for uncertain, 4 for probably no caries, and 5 for definitely no caries.	• **Intraoral Camera (DIAGNOcam)** SE = 79%, SP = 88%, AUC = 0.83 • **Digital Radiography (BWR)** SE = 74%, SP = 85%, AUC = 0.80	• Two trained examiners (one oral and maxillofacial radiologist and a restorative dentistry consultant) independently evaluated the diagnostic tools (DIAGNOcam and radiography) • Examiners underwent calibration sessions using a set of guidelines and caries classification criteria.	DIAGNOcam showed comparable diagnostic performance to PSP-BWR, with a slightly higher AUC due to its ability to visualize early carious lesions without radiation. The authors highlighted DIAGNOcam’s potential as an alternative diagnostic tool, complementing radiographs for early detection and patient education, and improving patient comfort during the diagnostic process.
Markowitz et al. [47]	Experimental study (In-vitro)	**Teeth Details** Permanent teeth, *N* = 90 (all molars) **Participant details** NA	**Intraoral Camera** • Brand name Spectra Caries Detection Aid by Air Techniques	A numerical scoring system provided by the Spectra Caries Detection Aid	Histological	Histological	The study utilized both the manufacturer’s recommended cutoff of 2.0 and an alternative cutoff of 1.8 for diagnosing dentin caries.	• **Manufacturer’s Recommended Cutoff (2.0)** SE = 68%, SP = 78%, AUC = 0.82, False Positive Rate = 21.62%, False Negative Rate = 32.08% • **Alternative Cutoff (1.8)** SE = 87%, SP = 70%, False Positive Rate = 29.73%, False Negative Rate = 13.21%	• The number of examiners is not mentioned • The principal investigators trained the examiners, who then evaluated 10 teeth using the Spectra Caries Detection Aid. • The calibration process aimed to ensure that their measurements were within ±0.2 of each other.	The authors recommended that the manufacturer’s cutoff value of 2.0 as a balanced threshold for diagnosing dentin caries. They noted that this cutoff minimizes false positives (21.62%) compared to a lower cutoff, such as 1.8, thus reducing the likelihood of overtreating early lesions. This might benefit more from preventive care, such as sealants or remineralization.
Ko et al. [5]	Experimental study (In-vitro)	**Teeth Details** Permanent teeth, *N* = 95 (molars and premolars) **Participant details** NA	**Intraoral camera** • Brand name Quantitative Light-Induced Fluorescence-Digital (QLF-D). Specific brand name is unclear. **Digital Radiography** • Brand name Kodak 2200 Intraoral X-ray System by Kodak	International Caries Detection and Assessment System II (ICDAS-II)	Histological	Visual and Radiography	Optimal thresholds for the QLF-D device were determined via ROC analysis: DF > −13.8 for enamel caries and DF > −28.3 for dentine caries.	• **ICDAS-II (Visual)** **Enamel** SE = 80%, SP = 68%, AUC = 0.74 **Dentin** SE = 64%, SP = 68%, AUC = 0.66 • **Intraoral camera (QLF-D)** **Enamel** SE = 75%, SP = 84%, AUC = 0.80 **Dentin** SE = 64%, SP = 88%, AUC = 0.76 • **Digital Radiography** **Enamel** SE = 71%, SP = 89%, AUC = 0.80 **Dentin** SE = 50%, SP = 94%, AUC = 0.72	• One experienced dentist was used as the sole examiner. • The examiner received 90 min of training with the ICDAS-II system through an e-learning program • The examiner discussed the radiographic scoring criteria with a radiology specialist.	QLF-D intraoral camera showed comparable diagnostic performance to both ICDAS-II and digital radiography, especially for detecting dentine caries. The authors suggested a cutoff of -13.8 for enamel and -28.3 for dentine lesions to accurately identify the extent of caries lesions while using the QLF-D devices.
Jablonski-Momeni et al. [18]	Comparative study (In-vivo)	**Teeth Details** Permanent teeth, *N* = 306 (154 premolars and 152 molars), **Participant details**, *N* = 26 (10 male and 16 female), age range = 21.4–43 years	**Intraoral camera** • Brand name VistaProof by Durr Dental	International Caries Detection and Assessment System (ICDAS)	Radiography and Histological	Visual and Radiography	The study used the following manufacturer-specified fluorescence thresholds for VistaProof, 0.0–0.9 for sound enamel, 1.0–1.4 for early enamel caries, 1.5–1.9 for advanced enamel caries, 2.0–2.4 for dentine caries and >2.4 for deep dentine caries.	• **Intraoral Camera (Vista Proof)** **Enamel Lesions** SE = 92.3%, SP = 41.1%, AUC = 0.82, PPV = 13.9%, NPV = 98.1%, Overall Accuracy = 46.0% **Dentin Lesions** SE = 25.9%, SP = 97.9%, AUC = 0.85, PPV = 53.8%, NPV = 93.2% Overall Accuracy = 91.0%,	Two experienced examiners carried out the ICDAS classification procedure. Among them, only one observer took the images using VistaProof.	The VistaProof camera exhibited high SE for enamel lesions and SP for dentine lesions, making it effective for early caries detection and reducing false positives for severe stages. Combining VistaProof with ICDAS visual scoring enhanced SE for dentine caries, highlighting the value of integrated diagnostic methods. Its high negative predictive value makes it particularly reliable for ruling out caries in low-caries populations, minimizing unnecessary treatments.
Boye et al. [4]	Comparative study (In-vitro)	**Teeth Details** Permanent teeth, *N* = 50 (32 molars, 18 premolars) **Participant details** NA	**Intra-oral Camera** • Brand name Sopro 717 by The Acteon Group Eaton Socon	British Association for the Study of Community Dentistry (BASCD)	Histological	Visual analysis using Photographs	Based on BASCD diagnostic scoring the study selected the threshold from Caries into Dentine	• **BASCD (Visual)** SE = 65.5%, SP = 82.4% • **Intraoral Camera** SE = 81.3%, SP = 82.4%	• Nine examiners were included in this study. Among them, 3 were the United Kingdom National Epidemiological Survey members. • Training was provided based on BASCD protocol.	The photographic method demonstrated higher SE while maintaining similar SP against visual examination. This makes it a viable option for caries detection in epidemiological studies.
Jablonski-Momeni et al. [49]	Experimental study (In-vitro)	**Teeth details** Permanent teeth, *N* = 101 (65 molars, 36 premolars) **Participant details** NA	**Intraoral Camera** • Brand name VistaCam iX and VistaProof by Durr Dental	International Caries Detection and Assessment System (ICDAS-II)	Histological	Visual	Based on fluorescence intensity values the study selected optimal cutoff 1.2/1.3 for enamel and dentine lesions and 1.4/1.5 for dentine-only lesions.	• **ICDAS-II (Visual)** SE = 97.9%, SP = 80%, AUC = 0.96 (Combined examiner) • **Intraoral Camera** 1. **VistaCam iX (FC1)** **Enamel and Dentin** SE = 76.6% (Examiner A), 80.5% (Examiner B) SP = 86.7% (both examiners) AUC = 0.87 (Examiner A), 0.92 (Examiner B) **Dentin Only** SE = 86.0% (Examiner A), 88.4% (Examiner B) SP = 67.3% (both examiners) AUC = 0.87 (both examiners) 2. **VistaProof (FC2)** **Enamel and Dentin** SE = 81.8% (Examiner A), 87.0% (Examiner B) SP = 3.3% (Examiner A), 100% (Examiner B), AUC = 0.93 (Examiner A), 0.96 (Examiner B) **Dentin Only** SE = 90.7% (Examiner A), 95.3% (Examiner B) SP = 77.6% (Examiner A), 71.4% (Examiner B), AUC = 0.93 (Examiner A), 0.91 (Examiner B)	• Two examiners (A and B) participated in the study. • Examiner A had 12 years of device experience; Examiner B was a dental student without specific training. • Detailed training information is not specified • Both independently assessed teeth twice with VistaCam iX and VistaProof for reproducibility.	Both devices demonstrated high reproducibility and strong correlation with histology, indicating that VistaCam iX (new technology) can reliably replace VistaProof without diagnostic loss. The authors also noted that these intraoral cameras can provide objective, consistent caries detection, especially useful where visual assessments are limited.
Jablonski-Momeni et al. [48]	Comparative study (In-vitro)	**Teeth Details** Permanent teeth, *N* = 53 (47 molars, 6 premolars) **Participant details** NA	**Intraoral camera** • Brand name Vista Proof by Durr Dental	International Caries Detection and Assessment System (ICDAS-II) and Downer histological classification system	Histological	Visual	The study used manufacturer-defined thresholds for the VistaProof camera, 0.0–0.9 for sound enamel, >0.9–1.5 for initial caries (enamel lesions), and >1.5–2.0 for enamel caries to the dentinoenamel junction and >2.0 for dentin caries.	• **Intraoral camera (Vista Proof)** **Enamel and Dentin** SE = 71% to 86%, SP = 32% to 76%, AUC = 0.77 (Examiner A), 0.75 (Examiner B), Optimal Cutoff = 1.2/1.3 for highest SE and specificity. **Dentin only** SE = 4% to 91%, SP = 56% to 99%, AUC = 0.81 (Examiner A), 0.77 (Examiner B), Optimal Cutoff = 1.3/1.4 for highest SE and specificity.	• Two examiners were included – one was an experienced dentist, and the one was a final-year dental student • The experienced dentist had prior training in using the ICDAS-II system. The dental student received specific training in both theoretical and practical aspects of the system. • Device-related training is unclear	The authors observed that the SE and SP varied with different cutoff values while using Vista Proof, emphasizing that appropriate cutoff points are critical for optimizing diagnostic accuracy. For instance, for enamel and dentin lesions, the optimal cutoff provided a balanced SE and SP that could help in identifying early carious lesions without overdiagnosing sound tissue.
Ferreira Zandona et al. [13]	Comparative study (In-vitro)	**Teeth Details** Permanent teeth, *N* = 150 (all premolars) **Participant details** NA	**Intraoral camera** • Brand name Dye-Enhanced Laser Fluorescence (DELF, no specific brand name was provided)	The authors used a binary scoring system along with color ratings	Histological	Visual	The study used ROC analysis to define an optimal cutoff of maximum lesion depth >50 µm for DELF.	• **Binary scoring (Visual)** SE = 0.03, SP = 1.00 • **Scoring with color ratings (Visual)** SE = 0.47, SP = 0.70 • **Intraoral Camera** 1. Laser Fluorescence (LF) SE = 49%, SP = 67% 2. Dye-Enhanced Laser Fluorescence (DELF) SE = 72%, SP = 60%	• Two examiners independently conducted the assessment. • Both examiners participated in two training sessions, in which they examined a set of 20 additional teeth using all 3 methods.	DELF showed the highest SE with a moderate SP among the methods, which makes the device clinically valuable for identifying initial demineralization in occlusal pits and fissures compared to traditional visual examination.

Details include sample types, examiner training, caries scoring criteria, and calibration methods.

*SE* Sensitivity, *SP* Specificity, *PPV* Positive Predictive Value, *AUC* Area Under Cover, *NPV* Negative Predictive Value, *BWR* Bite Wing Radiograph, *PR* Panoramic Radiograph, *ROC* Receiver Operating Characteristic Curve, *EDJ* Enamel-Dentin Junction, *ICDAS* International Caries Detection and Assessment System, *ICCMS* International Caries Classification and Management System.

### Risk of bias assessment (JBI)

This assessment aligns with the broader evaluation of 28 selected studies using the JBI DTA checklist, which demonstrated a consistently high level of methodological rigor. All studies were classified as low risk of bias (Scored > 70%). Among them, 3 studies (10.7%) achieved the maximum score of 10 ‘Positive’ responses, indicating strong adherence to all evaluated criteria, including appropriate sample selection and blinded interpretation of test results. The most common methodological shortcomings were the absence of pre-specified threshold values, lack of independence between index and reference tests, and inconsistent application of reference standards. Specifically, 4 studies (14.3%) had a single methodological limitation, such as unclear reporting of observer blinding or patient sampling criteria. The majority (39.3%) had 2 methodological concerns, while 35.7% exhibited 3, placing them at the lower threshold of the low-risk category. Full scoring details are available in Supplementary Table 5.

**Fig. 2:**
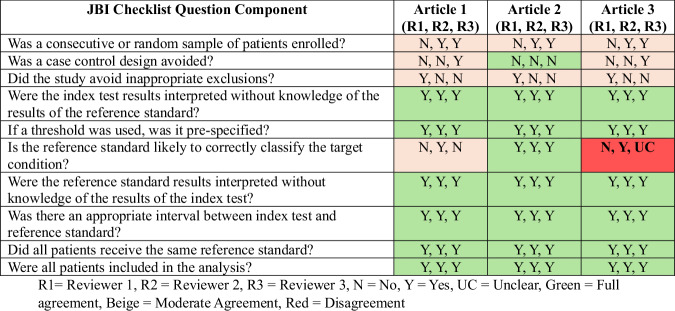
Confusion matrix of the reviewer’s responses on the JBI appraisal tool.

To assess inter-reviewer reliability, 3 randomly selected articles (see Supplementary Table 5) were independently evaluated by 3 reviewers (FR, JD, and THF), who were blinded to one another’s assessments. The Fleiss’ kappa value was *k* = 0.17, indicating slight agreement among the reviewers. As illustrated in the confusion matrix (Fig. 2), the greatest discrepancies occurred in items related to reference standard independence, sample selection, and the avoidance of inappropriate exclusions. These components of the JBI checklist are known to be subject to greater variability in interpretation, particularly in studies with limited or ambiguous reporting [33, 34], which likely contributed to the slight inter-reviewer disagreement observed in this study. All disagreements were resolved through structured discussions, with reviewers referring to the JBI manual for clarification where needed. No inclusion or exclusion decisions were affected by inter-reviewer variability.

## Results of meta-analysis

Sixteen articles met the threshold score of 5 on the modified NOS and were included in this meta-analysis. Table 2 summarizes diagnostic accuracy metrics (SE, SP, TP, FP, FN, TN) for intraoral scanners and cameras, grouped by examiner, lesion type, lesion location, and comparator tool. A Python code script for data processing and analysis is added in Supplementary Table 6.

**Table 2:** Summary of data extracted for meta-analysis across included studies.

Author (Year)	Number of Examiners	Sample Count	Intraoral device (Brand name)	Comparable tool 1	Comparable tool 2	Comparable tool 3	Reference Standard	Lesion type	Lesion Location	SE, SP, TP, FP, FN, TN of intraoral devices	SE, SP, TP, FP, FN, TN of comparable tool 1	SE, SP, TP, FP, FN, TN of a comparable tool 2	SE, SP, TP, FP, FN, TN of comparable tool 3
Patel et al. [43]	Examiner 1	466	Intraoral Camera	Visual	NA	NA	Radiography	Enamel	Proximal	SE = 92%, SP = 57%, TP = 214, FP = 100, FN = 19, TN = 133	SE = 95%, SP = 37%, TP = 221, FP = 147, FN = 12, TN = 86	NA	NA
										SE = 36%, SP = 88%, TP = 84, FP = 28, FN = 149, TN = 205	SE = 2%, SP = 99%, TP = 5, FP = 2, FN = 228, TN = 231		
								Dentin		SE = 44%, SP = 98%, TP = 103, FP = 5, FN = 130, TN = 228	SE = 62%, SP = 93%, TP = 144, FP = 16, FN = 89, TN = 217		
Cuenin et al. [6]	Examiner 1	344	Intraoral Scanner (iTero Element 5D)	NA	Radiography	NA	Radiography	Supragingival	Proximal	SE = 23%, SP = 96%, TP = 9, FP = 30, FN = 12, TN = 293	NA	SE = 24%, SP = 97%, TP = 9, FP = 30, FN = 9, TN = 296	NA
Ntovas et al. [44]	Examiner 1	112	Intraoral Scanner (TRIOS 4, ALG1)	Visual	Intraoral Scanner (TRIOS 4)	NA	Histological	Enamel	Occlusal	SE = 75%, SP = 71%, TP = 37, FP = 11, FN = 13, TN = 34	SE = 82%, SP = 50%, TP = 41, FP = 19, FN = 0, TN = 28	SE = 74%, SP = 65%, TP = 36, FP = 14, FN = 14, TN = 31	NA
								Dentin		SE = 100%, SP = 96%, TP = 53, FP = 2, FN = 0, TN = 56	SE = 100%, SP = 99%, TP = 53, FP = 1, FN = 0, TN = 58	SE = 100%, SP = 96%, TP = 53, FP = 2, FN = 0, TN = 56	NA
Saffarpour et al. [7]	Examiner 1	80	Intraoral camera (VC iX)	Visual	Radiography	NA	Histological	Enamel	Occlusal	SE = 92%, SP = 50%, TP = 36, FP = 20, FN = 3, TN = 20	SE = 85%, SP = 95%, TP = 36, FP = 2, FN = 6, TN = 38	SE = 45%, SP = 94%, TP = 19, FP = 4, FN = 23, TN = 30	NA
								Dentin		SE = 81%, SP = 40%, TP = 18, FP = 12, FN = 4, TN = 18	SE = 73%, SP = 100%, TP = 22, FP = 0, FN = 8, TN = 40	SE = 35%, SP = 98%, TP = 8, FP = 1, FN = 15, TN = 39	
Wang et al. [21]	Examiner 1	118	Intraoral camera (DC)	Visual	Radiography	NA	Histological	Enamel	Proximal	SE = 68%, SP = 94%, TP = 40, FP = 2, FN = 19, TN = 31	SE = 33%, SP = 94%, TP = 21, FP = 2, FN = 43, TN = 31	SE = 47%, SP = 94%, TP = 24, FP = 2, FN = 27, TN = 31	NA
										SE = 75%, SP = 97%, TP = 24, FP = 1, FN = 8, TN = 32	SE = 46%, SP = 98%, TP = 15, FP = 1, FN = 18, TN = 32	SE = 54%, SP = 98%, TP = 17, FP = 1, FN = 14, TN = 32	
								Dentin		SE = 84%, SP = 98%, TP = 21, FP = 0, FN = 4, TN = 29	SE = 52%, SP = 100%, TP = 13, FP = 0, FN = 12, TN = 29	SE = 88%, SP = 99%, TP = 22, FP = 1, FN = 3, TN = 29	
Valizadeh et al. [37]	Examiner 1	40	Intraoral camera (VC iX Proxi)	NA	Radiography	NA	Histological	Enamel	Proximal	SE = 100%, SP = 71%, TP = 5, FP = 2, FN = 0, TN = 5	NA	SE = 40%, SP = 87.6%, TP = 2, FP = 1, FN = 3, TN = 6	NA
								Dentin		SE = 73%, SP = 71%, TP = 8, FP = 2, FN = 3, TN = 5		SE = 55%, SP = 86%, TP = 6, FP = 1, FN = 5, TN = 6	
										SE = 82%, SP = 71%, TP = 14, FP = 2, FN = 3, TN = 5		SE = 59%, SP = 86%, TP = 10, FP = 1, FN = 7, TN = 6	
Michou et al. [41]	Examiner 1	118	Intraoral Scanner (TRIOS 4, ALG1, ALG2, ALG3, ALG4)	Visual	NA	NA	Histological	Enamel	Occlusal	ALG1 SE = 74%, SP = 53%, TP = 44, FP = 15, FN = 28, TN = 31	SE = 82%, SP = 59%, TP = 48, FP = 11, FN = 24, TN = 35	NA	NA
										ALG2 SE = 70%, SP = 59%, TP = 41, FP = 18, FN = 24, TN = 35			
										ALG3 SE = 56%, SP = 100%, TP = 33, FP = 26, FN = 0, TN = 59			
										ALG4 SE = 71%, SP = 59%, TP = 42, FP = 17, FN = 24, TN = 35			
										ALG1 SE = 85%, SP = 53%, TP = 50, FP = 9, FN = 28, TN = 31			
										ALG2 SE = 81%, SP = 59%, TP = 48, FP = 11, FN = 24, TN = 35			
										ALG3 SE = 59%, SP = 71%, TP = 35, FP = 24, FN = 17, TN = 42			
										ALG4 SE = 80%, SP = 59%, TP = 47, FP = 12, FN = 24, TN = 35			
								Dentin		ALG1 SE = 89%, SP = 55%, TP = 53, FP = 6, FN = 27, TN = 33			
										ALG2 SE = 71%, SP = 62%, TP = 42, FP = 17, FN = 22, TN = 37			
										ALG3 SE = 67%, SP = 91%, TP = 40, FP = 19, FN = 5, TN = 54			
										ALG4 SE = 78%, SP = 88%, TP = 46, FP = 13, FN = 7, TN = 52			
										ALG3 SE = 73%, SP = 88%, TP = 43, FP = 16, FN = 7, TN = 52			
										ALG4 SE = 91%, SP = 86%, TP = 54, FP = 5, FN = 8, TN = 51			
										ALG1 SE = 100%, SP = 46%, TP = 59, FP = 0, FN = 32, TN = 27			
										ALG2 SE = 47%, SP = 58%, TP = 28, FP = 31, FN = 25, TN = 34			
										ALG3 SE = 72%, SP = 89%, TP = 42, FP = 17, FN = 6, TN = 53			
										ALG4 SE = 84%, SP = 85%, TP = 50, FP = 9, FN = 9, TN = 50			
										ALG3 SE = 81%, SP = 88%, TP = 48, FP = 11, FN = 7, TN = 52			
										ALG4 SE = 88%, SP = 83%, TP = 52, FP = 7, FN = 10, TN = 49			
Michou et al. [41]	Examiner 1	158	Intraoral Scanner (TRIOS 4)	Visual	Radiography	Intraoral Camera (DC)	Histological	Enamel	Proximal	SE = 87%, SP = 68%, TP = 32, FP = 8, FN = 7, TN = 35	SE = 39%, SP = 92%, TP = 31, FP = 48, FN = 6, TN = 73	NA	SE = 74%, SP = 85%, TP = 65, FP = 16, FN = 20, TN = 90
										SE = 66%, SP = 89%, TP = 52, FP = 27, FN = 9, TN = 70	SE = 28%, SP = 97%, TP = 22, FP = 57, FN = 2, TN = 77		SE = 69%, SP = 88%, TP = 55, FP = 24, FN = 9, TN = 70
								Dentin		SE = 83%, SP = 91%, TP = 73, FP = 12, FN = 15, TN = 72	SE = 30%, SP = 98%, TP = 24, FP = 55, FN = 2, TN = 77		SE = 68%, SP = 92%, TP = 58, FP = 8, FN = 27, TN = 99
										SE = 70%, SP = 98%, TP = 55, FP = 24, FN = 2, TN = 77			SE = 59%, SP = 98%, TP = 47, FP = 32, FN = 2, TN = 77
	Examiner 2							Enamel		SE = 86%, SP = 53%, TP = 68, FP = 49, FN = 11, TN = 54	NA		SE = 74%, SP = 85%, TP = 65, FP = 16, FN = 20, TN = 90
										SE = 52%, SP = 90%, TP = 41, FP = 38, FN = 9, TN = 71			SE = 50%, SP = 92%, TP = 40, FP = 39, FN = 6, TN = 73
								Dentin		SE = 66%, SP = 90%, TP = 58, FP = 15, FN = 30, TN = 99			SE = 68%, SP = 92%, TP = 58, FP = 8, FN = 27, TN = 99
										SE = 63%, SP = 98%, TP = 50, FP = 29, FN = 2, TN = 77			SE = 70%, SP = 96%, TP = 55, FP = 24, FN = 3, TN = 76
	Examiner 3							Enamel		NA	NA	SE = 49%, SP = 92%, TP = 55, FP = 13, FN = 57, TN = 104	NA
												SE = 49%, SP = 91%, TP = 39, FP = 40, FN = 7, TN = 71	
								Dentin		NA	NA	SE = 51%, SP = 98%, TP = 55, FP = 5, FN = 53, TN = 104	
												SE = 63%, SP = 100%, TP = 50, FP = 29, FN = 0, TN = 79	
Michou et al. [40]	Examiner 1	139	Intraoral Scanner (TRIOS 3, ALG1, ALG2, ALG3, ALG4)	Visual	Radiography	NA	Histological	Enamel	Occlusal	ALG1 SE = 84%, SP = 81%, TP = 26, FP = 6, FN = 5, TN = 28	SE = 93%, SP = 88%, TP = 33, FP = 4, FN = 2, TN = 31	SE = 60%, SP = 93%, TP = 28, FP = 2, FN = 21, TN = 30	NA
										ALG2 SE = 82%, SP = 83%, TP = 25, FP = 5, FN = 6, TN = 29			
										ALG3 SE = 80%, SP = 84%, TP = 40, FP = 10, FN = 115, TN = 280			
										ALG4 SE = 91%, SP = 77%, TP = 30, FP = 7, FN = 3, TN = 24			
								Dentin		ALG1 SE = 76%, SP = 88%, TP = 29, FP = 4, FN = 9, TN = 35	SE = 59%, SP = 98%, TP = 21, FP = 1, FN = 14, TN = 34	SE = 58%, SP = 100%, TP = 29, FP = 0, FN = 21, TN = 30	
										ALG2 SE = 76%, SP = 84%, TP = 28, FP = 5, FN = 10, TN = 34			
										ALG3 SE = 72%, SP = 87%, TP = 27, FP = 5, FN = 11, TN = 34			
										ALG4 SE = 82%, SP = 85%, TP = 32, FP = 8, FN = 7, TN = 35			
Iranzo Cortes et al. [38]	Examiner 1	65	Intraoral camera (VP)	Visual	Intraoral + Visual	NA	Histological	Enamel	Occlusal	SE = 70.5%, SP = 81.0%, TP = 31, FP = 4, FN = 13, TN = 17	SE = 79.6%, SP = 81.0%, TP = 35, FP = 4, FN = 9, TN = 17	SE = 84.1%, SP = 71.4%, TP = 37, FP = 6, FN = 7, TN = 15	NA
	Examiner 2									SE = 81.8%, SP = 61.9%, TP = 36, FP = 8, FN = 8, TN = 13	SE = 86.4%, SP = 95.2%, TP = 38, FP = 1, FN = 6, TN = 20	SE = 95.5%, SP = 61.9%, TP = 42, FP = 8, FN = 2, TN = 13	
Tonkaboni et al. [19]	Examiner 1	108	Intraoral camera (Vc iX Proxi)	Visual	Radiography	NA	Histological	CEJ	Proximal	SE = 100%, SP = 83%, TP = 54, FP = 13, FN = 0, TN = 66	SE = 54%, SP = 100%, TP = 29, FP = 0, FN = 25, TN = 79	SE = 54%, SP = 100%, TP = 29, FP = 0, FN = 25, TN = 79	NA
										SE = 16%, SP = 100%, TP = 9, FP = 0, FN = 45, TN = 80	SE = 52%, SP = 100%, TP = 28, FP = 0, FN = 26, TN = 80	SE = 56%, SP = 98%, TP = 53, FP = 2, FN = 0, TN = 56	
										SE = 100%, SP = 96%, TP = 51, FP = 2, FN = 0, TN = 52	SE = 49%, SP = 100%, TP = 30, FP = 0, FN = 24, TN = 80	SE = 37%, SP = 100%, TP = 20, FP = 0, FN = 33, TN = 80	
Ko et al. [5]	Examiner 1	95	Intraoral camera (QLF-D)	Visual	Radiography	NA	Histological	Enamel and Dentin	Proximal	SE = 75%, SP = 84%, TP = 47, FP = 6, FN = 16, TN = 32	SE = 80%, SP = 68%, TP = 50, FP = 12, FN = 7, TN = 26	SE = 71%, SP = 89%, TP = 45, FP = 5, FN = 18, TN = 34	NA
								Dentin		SE = 64%, SP = 88%, TP = 40, FP = 4, FN = 22, TN = 34	SE = 64%, SP = 68%, TP = 40, FP = 12, FN = 22, TN = 26	SE = 50%, SP = 94%, TP = 31, FP = 2, FN = 31, TN = 37	
Jablons kiMomeni et al. [18]	Examiner 1	306	Intraoral camera (VP)	Visual	Radiography	NA	Radiography and Histological	Enamel	Occlusal	SE = 92%, SP = 41%, TP = 300, FP = 180, FN = 25, TN = 70	NA	NA	NA
								Dentin		SE = 26%, SP = 98%, TP = 40, FP = 10, FN = 115, TN = 280			
Boye et al. [4]	Examiner 1	50	Intraoral camera (SP 717)	Visual	NA	NA	Histological	Dentin	Occlusal	SE = 81.3%, SP = 82.4%, TP = 25, FP = 4, FN = 7, TN = 19	SE = 65.5%, SP = 82.4%, TP = 20, FP = 4, FN = 12, TN = 19	NA	NA
Jablons kiMomeni et al. [49]	Examiner 1	97	Intraoral Camera (VC iX)	Visual	Intraoral camera (VP)	NA	Histological	Enamel and Dentin	Occlusal	SE = 77%, SP = 87%, TP = 36, FP = 8, FN = 15, TN = 52	SE = 97.9%, SP = 80%, TP = 43, FP = 12, FN = 1, TN = 48	SE = 82%, SP = 93%, TP = 39, FP = 4, FN = 12, TN = 56	NA
								Dentin		SE = 86%, SP = 67%, TP = 43, FP = 17, FN = 7, TN = 40	SE = 71%, SP = 96%, TP = 36, FP = 2, FN = 25, TN = 48	SE = 91%, SP = 78%, TP = 45, FP = 13, FN = 5, TN = 46	
	Examiner 2							Enamel and Dentin		SE = 81%, SP = 87%, TP = 38, FP = 8, FN = 14, TN = 52	SE = 96%, SP = 78%, TP = 42, FP = 14, FN = 2, TN = 46	SE = 87%, SP = 100%, TP = 41, FP = 0, FN = 10, TN = 60	
								Dentin		SE = 88%, SP = 67%, TP = 44, FP = 17, FN = 6, TN = 40	SE = 69%, SP = 95%, TP = 35, FP = 3, FN = 15, TN = 47	SE = 95%, SP = 71%, TP = 48, FP = 18, FN = 2, TN = 42	
Zandona et al. [13]	Examiner 1	150	Intraoral Camera (LF)	Visual	Intraoral camera (DELF)	NA	Histological	Enamel	Occlusal	SE = 49%, SP = 67%, TP = 74, FP = 25, FN = 50, TN = 101	Binary scoring SE = 0.03, SP = 1.00, TP = 74, FP = 33, FN = 76, TN = 117	SE = 72%, SP = 60%, TP = 108, FP = 60, FN = 30, TN = 90	NA
											Scoring with color ratings SE = 0.47, SP = 0.70, TP = 5, FP = 0, FN = 145, TN = 150		

Each row presents SE, SP, TP, FP, FN, and TN for intraoral diagnostic devices and their comparator(s), grouped by lesion type (enamel/dentin), location (e.g., occlusal, proximal), and Examiner Counts.

*SE* Sensitivity, *SP* Specificity, *TP* True Positive, *FP* False Positive, *FN* False Negative, *TN* True Negative, *NA* Not Available.

### Heterogeneity of the articles

The bivariate random-effects analysis provided pooled estimates of 50% for both SE and SP, indicating moderate diagnostic accuracy for IOSs and IOCs in detecting dental caries. These values suggest that intraoral diagnostic tools performed similarly in identifying true positive and negative cases, though with limited precision.

Heterogeneity analysis showed a between-study variance (Tau^2^) of 0.32 for both SE and SP, indicating moderate variability across studies. This degree of heterogeneity is expected in diagnostic accuracy reviews involving diverse populations, methods, and diagnostic devices. In this analysis, variability likely stemmed from differences in study design (in-vivo vs. in-vitro), diagnostic criteria (e.g., ICDAS, UniViSS), lesion types and locations, the type of intraoral device used (IOSs vs. IOCs), and examiner-dependent factors such as training and calibration.

While the bivariate model statistically accounts for heterogeneity, the observed variation highlights the challenges in standardizing caries detection practices and suggests that diagnostic performance is influenced by contextual and technical factors. Subgroup analyses were therefore performed to further explore the effects of these sources of variability on diagnostic accuracy.

The estimated correlation between SE and SP was *r* = 0, suggesting no observable trade-off or inverse relationship between the two measures. In bivariate models, a negative correlation typically indicates a threshold effect, where increasing SE reduces SP and vice versa [32]. The absence of such a pattern in this analysis may reflect consistent diagnostic thresholds or homogeneous reporting practices across the included studies.

### Comparison of diagnostic methods

The Welch’s ANOVA results highlighted significant differences in SE (*F* = 9.27, *p* < 0.001) and SP (*F* = 6.60, *p* < 0.001) among the diagnostic methods, emphasizing their varied capabilities in detecting carious lesions and avoiding false positives. The Games-Howell post hoc analysis revealed that IOSs exhibited the highest SE and significantly outperformed radiography (mean difference = 0.23, *p* < 0.001), demonstrating superior reliability in identifying true positive cases. Similarly, IOCs showed higher SE compared to radiography (mean difference = 0.22, *p* < 0.001), highlighting the limitations of radiographic techniques in detecting carious lesions.

Visual methods demonstrated moderate SE but did not differ significantly from IOCs (mean difference = 0.14, *p* = 0.11) or radiography (mean difference = −0.08, *p* = 0.56). The near-significant trend between visual methods and IOCs may suggest a marginal advantage in SE for IOCs, potentially reflecting improved surface-level visualization due to digital magnification or real-time imaging. However, this trend should be interpreted with caution given the variability across included studies and the influence of examiner-dependent factors.

For SP, radiography emerged as the most reliable method for avoiding false positives, achieving the highest SP. It significantly outperformed IOCs (mean difference = −0.14, *p* < 0.001) and IOSs (mean difference = −0.16, *p* < 0.00) in accurately detecting non-carious lesions. Visual methods displayed moderately high SP and outperformed IOSs (mean difference = -0.09, *p* < 0.001) but showed no significant differences compared to IOCs (mean difference = −0.07, *p* < 0.001) or radiography (mean difference = 0.07, *p* < 0.001).

### Lesion location

The Welch ANOVA results reveal highly significant differences in both SE (*F* = 5.02, *p* < 0.001) and SP (*F* = 3.37, *p* < 0.001) across IOCs, IOSs, radiography, and visual methods, indicating that the diagnostic performance of these tools varies significantly depending on the lesion type and diagnostic metrics. The Games-Howell post hoc analysis for SE further clarified these differences. For occlusal lesions, IOCs and IOSs demonstrated trends toward higher SE compared to radiography (mean difference = 0.36 and 0.32, *p* = 0.07 and *p* = 0.09, respectively). Although not statistically significant, these findings may reflect the well-recognized limitations of radiography in detecting early or surface-level occlusal lesions. The observed trends may also be influenced by heterogeneity in examiner calibration, device imaging geometry, and lesion interpretation across studies. These nuances highlight the diagnostic complexity of occlusal lesions and suggest a potential advantage of IOSs and IOCs in this context that needs further investigation.

In contrast, radiography demonstrated significantly higher SP for occlusal lesions compared to IOCs (mean difference = −0.25, *p* < 0.001) and IOSs (mean difference = −0.21, *p* = 0.19), establishing it as the most reliable tool for ruling out false positives in this context. Interestingly, no significant differences in SE were observed between IOSs and visual methods (mean difference = 0.04, *p* = 0.96) or between IOSs and IOCs (mean difference = 0.04, *p* = 0.68), suggesting comparable diagnostic performance for these tools in terms of detecting true positives.

For proximal lesions, the analysis showed no significant differences in either SE or SP across the diagnostic tools. The lack of significant differences for proximal lesions may reflect the uniformly limited visibility of these lesions across all diagnostic modalities, emphasizing the intrinsic diagnostic challenges of interproximal surfaces regardless of the tool used. IOCs and radiography performed similarly in SE (mean difference = 0.16, *p* = 0.42) and SP (mean difference = −0.09, *p* = 0.09). Radiography and visual methods also displayed nearly identical SE (mean difference = 0.004, *p* = 0.99) and SP (mean difference = 0.02, *p* = 0.93) for proximal lesions, indicating comparable performance for these tools.

These findings highlight the nuanced interplay between SE and SP across lesion types. Radiography demonstrated superior SP for occlusal lesions, while IOCs and IOSs showed trends toward higher SE. The absence of significant differences for proximal lesions underscores the need for further investigation into lesion-specific diagnostic challenges and the development of tailored strategies for optimal caries detection.

### Lesion type

For lesion types, pooled SE was consistently at 50% (95% CI: 0.45–0.55) for CEJ, enamel, and dentin lesions, indicating moderate diagnostic performance. However, enamel and dentin combined lesions showed significantly higher SE (77.3%; 95% CI: 0.68–0.84), reflecting improved accuracy in detecting multi-layered caries. In contrast, supragingival lesions had the lowest SE (23%; 95% CI: 0.21–0.25), with no observed heterogeneity (Tau² = 0). SP for supragingival lesions was exceptionally high (96%; 95% CI: 0.96–0.97), suggesting strong true-negative detection (non-caries) despite poor SE. For other lesion types, SP remained consistent at 50%, with moderate variability (Tau² = 0.1).

**Fig. 3:**
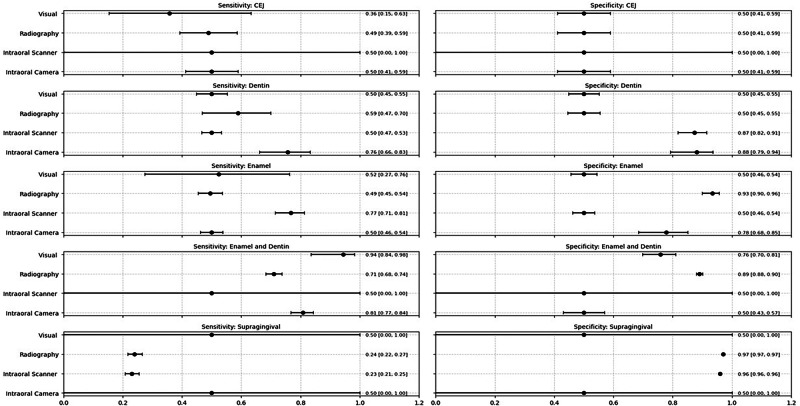
Pooled sensitivity and specificity (95% CI) of visual inspection, radiography, intraoral scanners, and cameras across lesion types and locations (CEJ, enamel, supragingival, and enamel-dentin).

The high SP observed for supragingival lesions may be due to the visual distinctiveness of surface-level features, which makes non-carious surfaces easier to identify. However, the uniformly low SE across tools highlights the difficulty in detecting early-stage lesions in these regions, potentially due to shallow lesion depth or lighting artifacts. Figure 3 illustrates pooled SE and SP estimates across diagnostic methods and lesion types, highlighting variability in performance among modalities.

- **Intraoral camera (IOC):** Among the tools evaluated, IOCs demonstrated comparatively higher SE of 75.6% (95% CI: 0.66–0.83) and SP of 88.1% (95% CI: 0.79–0.93) for dentin lesions. However, this performance should be interpreted cautiously given the substantial heterogeneity (Tau² = 1.85), which may reflect from differences in study protocols, operator expertise on evaluating images, or device calibration. For CEJ lesions, IOCs exhibited no significant differences in SE and SP compared to radiography and visual methods, with values around 50% (95% CI: 0.45–0.55).
- **Intraoral scanner (IOS):** showed a pooled SE of 50% (95% CI: 0.46–0.53) and a slightly higher SP of 87.3% (95% CI: 0.82–0.91) for dentin lesions. While SE exhibited low heterogeneity (Tau² = 0.1), SP showed considerable variability (Tau² = 1.06). This may be attributed to differences in scanner software, examiner interpretation of carious lesions, reference standards, or the misclassification of non-carious conditions like staining or abrasion. Similar to IOCs, IOSs showed no significant differences in diagnostic performance for CEJ lesions, with values around 50% (95% CI: 0.45–0.55). For supragingival lesions, IOSs showed low SE (23.0%; 95% CI: 0.21–0.25) but high SP of 96.0% (95% CI: 0.95–0.96), indicating reliable exclusion of false positives.
- **Visual:** yielded the highest SE for enamel and dentin combined lesions (94.4%; 95% CI: 0.84–0.98), though this was accompanied by lower SP (75.8%; 95% CI: 0.69–0.81) and substantial heterogeneity (Tau² = 1.11). Variability may result from inconsistencies in examiner training protocol, subjective interpretation criteria, or inconsistencies in applying visual scoring systems such as ICDAS or UniViSS. These factors can impact reproducibility, especially when detecting early-stage lesions. For CEJ lesions, visual methods performed similarly to IOCs and radiography, with SE and SP near 50% (95% CI: 0.45–0.55).
- **Radiography:** outperformed other methods in diagnosing enamel and dentin combined lesions, with a pooled SE of 71.0% (95% CI: 0.68–0.74) and SP of 89.0% (95% CI: 0.88–0.90) with minimal variability (Tau² = 0), indicating strong consistency across studies. However, they were less effective for supragingival lesions, with low SE (24.0%; 95% CI: 0.22–0.27) while maintaining the highest SP (97.0%; 95% CI: 0.96–0.97), reinforcing its ability to minimize false positives. Similar to other diagnostic methods, radiography showed no significant differences in SE and SP for CEJ lesions, with pooled values of ~50% (95% CI: 0.45–0.55).

Notably, both IOSs and radiography performed poorly in detecting supragingival lesions, with SE values of 23.0% (95% CI: 0.21–0.25) and 24.0% (95% CI: 0.22–0.27), respectively. However, their high SP of 96.0% (95% CI: 0.95–0.96) for IOSs and 97.0% (95% CI: 0.96–0.97) for radiography, highlights their effectiveness in minimizing false positives for surface-level caries.

### Examiner-dependent variabilities

**Fig. 4:**
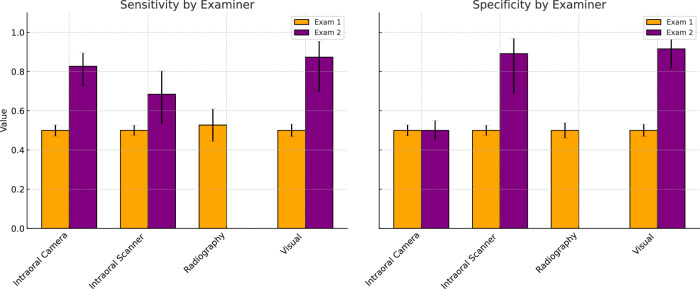
Examiner-dependent variability across all diagnostic tools.

Figure 4 illustrates examiner-dependent variability across all diagnostic tools. For IOCs, examiner 2 demonstrated a significantly higher SE of 82.7% (95% CI: 0.73–0.90) compared to examiner 1 (50.0%; 95% CI: 0.47–0.53). However, this result was accompanied by substantial heterogeneity (Tau² = 0.87), suggesting inconsistencies potentially related to differences in technique, experience, device familiarity, or calibration practices. In contrast, SE values for examiner 1 were more consistent, with minimal variability (Tau² = 0.1).

A similar trend was observed for IOSs, where examiner 2 achieved higher SE (68.4%; 95% CI: 0.54–0.80) and SP (89.1%; 95% CI: 0.69–0.97), though with moderate heterogeneity in SE (Tau² = 0.41) and high variability in SP (Tau² = 1.79). Examiner 1’s results with IOSs were more stable, with Tau² = 0.1 for both SE and SP, suggesting consistent, but reflected lower diagnostic accuracy.

For radiography, examiner 1 showed relatively low heterogeneity (Tau² = 0.46 for SE, 0.1 for SP), suggesting that radiographic techniques are less prone to examiner-related variability compared to intraoral imaging devices.

Visual methods displayed the greatest examiner-dependent variability. Examiner 2 achieved the highest SE (87.0%; 95% CI: 0.70–0.95) and SP (92.0%; 95% CI: 0.82–0.96), but SP was associated with notable heterogeneity (Tau² = 1.79). In contrast, examiner 1 demonstrated more consistent but lower performance (Tau² = 0.1 for both SE and SP).

These trends highlight that examiner-specific factors such as training, experience, technique, and protocol adherence significantly influence diagnostic outcomes, particularly for operator-dependent tools like IOCs, IOSs, and visual methods. For detailed confidence intervals and heterogeneity values associated with each examiner-tool combination, refer to Supplementary Fig. 1.

## Discussion

### Summary of key findings

This systematic review and meta-analysis evaluated the diagnostic performance of IOS and IOC compared to radiographic, visual, and histological methods. IOSs and IOCs demonstrated moderate diagnostic accuracy overall, with higher SE for occlusal and dentin lesions but variable SP. Radiography consistently showed the highest SP. All methods performed poorly in detecting supragingival lesions, primarily due to low SE. Examiner variability significantly influenced outcomes, particularly for image-based and visual methods, with better-trained examiners achieving higher accuracy but greater heterogeneity. These findings underscore the lesion-specific strengths of digital tools and the critical role of examiner training in diagnostic reproducibility.

### Performance of intraoral devices based on caries location

For occlusal lesions, IOSs and IOCs demonstrated slightly higher SE compared to radiography and visual examination, aligning with prior research [7, 35–37] that supports the role of fluorescence and near-infrared (NIR) technologies in enhancing early caries detection. These imaging technologies, such as differentiate healthy from demineralized tissues through light scattering mechanisms, which may be particularly beneficial in pediatric patients and in situations requiring minimal radiation exposure [6, 15]. However, variability in device mechanisms such as fluorescence shifts in IOCs versus NIR scattering in IOSs, may partly contribute to inconsistent SP, leading to more false-positive [38–40]. These inconsistencies may also be exacerbated by enamel defects (e.g., cracks, hypoplasia, fluorosis, molar-incisor hypomineralization) [7, 21, 41] and operator-dependent factors such as device positioning and image adjustments (e.g., brightness and contrast) [9, 36].

For proximal lesions, no statistically significant differences were observed in SE or SP across diagnostic tools. This finding is consistent with the known difficulties in detecting interproximal caries due to anatomical constraints and limited accessibility [42]. Despite the 3D imaging capability of IOSs, their clinical utility in proximal detection may be hindered by limited user experience and interpretive training with NIRI outputs [6, 10]. Previous studies have suggested [13, 15, 18] slightly better SP with radiography and marginally greater SE with visual methods [4, 13], particularly when combined with intraoral imaging. While no single method showed clear superiority, integrating complementary approaches may offer improved diagnostic accuracy. Notably, a high number of studies (12/28) [5, 6, 9–11, 15, 19–21, 23, 36, 37] in this review that targeted proximal lesions highlight the ongoing clinical interest in refining diagnostic strategies for these challenging sites.

Although the pooled SE and SP values for many devices hovered around 50%, such performance may be suboptimal in clinical settings. A 50% sensitivity means that half of true caries cases could go undetected, potentially delaying treatment [43]. Likewise, a 50% specificity raises the risk of false positives, which could lead to overtreatment, unnecessary restorations, and increased patient anxiety [20]. These limitations underscore the importance of examiner experience and suggest that digital tools should complement, rather than replace, clinical judgment.

### Performance of intraoral devices based on lesion type

#### Enamel-dentin lesions

Among lesion types, enamel-dentin lesions demonstrated the highest pooled SE (77.3%), suggesting that deeper and multi-layered caries were more easily detected across all diagnostic methods due to greater optical contrast and structural disruption. However, some studies [6, 22, 44] contradicted this meta-analysis by reporting lower SE values for intraoral imaging devices compared to radiography. These discrepancies may be attributed to differences in sample characteristics. While most studies evaluated permanent teeth [7, 12, 19, 20, 35, 38–40], a minority focused on primary teeth [6, 9, 15, 22, 23], which differ in enamel thickness and mineral content, affecting lesion visibility. Notably, only 3 of the 28 included studies involved primary teeth, compared to 25 using permanent dentition, highlighting a sample imbalance that may influence pooled estimates.

Radiography showed the highest SP, confirming its ability to rule out false positives and diagnose advanced lesions, likely due to its deeper penetration and reduced surface interference [45]. These results align with previous studies that consistently refer to radiography as the “gold standard” [8–10, 12, 22]. However, this term may be misleading, as diagnostic standards continue to evolve with advancements in digital imaging [11, 18, 46]. In this review, all radiographic assessments were performed using digital systems, such as Heliodent Plus & DS (4 studies) [8, 9, 11, 36], Kodak 2200 (3 studies) [5, 7, 19], and Planmeca (3 studies) [15, 21, 40], which offer improved image quality, lower radiation exposure, and adjustable contrast adjustments, demonstrating the shift away from traditional film-based bite wings.

Consistent with Wang et al. [21], this review observed substantial variation in the reference standards. Among the 28 included studies, histology was the most frequently used (16 studies) [4, 5, 7, 13, 19–21, 35, 37, 38, 40, 41, 44, 47–49], followed by visual examination (6 studies) [10, 12, 15, 22, 23, 39] and radiography (4 studies) [6, 9, 11, 36], with two studies combined or alternative approaches [8, 18]. Even within histology-based studies, sectioning techniques varied (e.g., mesiodistal [7, 13, 19], buccolingual [40, 41, 44], fissure [35, 49], longitudinal [38]), and several studies failed to specify their method. This inconsistency, identified in JBI appraisal as a frequent source of disagreement among reviewers, underscores the need for a standardized reference protocol in caries research.

While radiography remains important for detecting advanced caries, visual examination is particularly effective for identifying early surface changes that may not appear on radiographs. In this review, visual-tactile inspection showed high SE (94.4%) for enamel-dentin lesions, but lower SP (75.8%), likely due to its reliance on examiner training and calibration [7, 37]. Consistent with previous research [7, 9, 37, 41], the meta-analysis supports visual methods as a valuable initial screening tool, especially when standardized systems like ICDAS are used. Studies [9, 41] incorporating ICDAS-based visual assessment alongside intraoral imaging reported improved diagnostic accuracy for enamel and dentin lesions, reinforcing the clinical value of a multimodal approach. The methodological rigor observed in JBI appraised studies using standardized scoring further supports the integration of calibrated visual methods into comprehensive caries diagnostic protocols.

#### Supragingival lesions

Supragingival lesions, which represent early surface-level caries, posed the greatest diagnostic challenge across all evaluated methods. IOSs and IOCs showed the lowest pooled SE (23%), reflecting limited effectiveness in detecting non-cavitated enamel changes. This may be due to the high mineral density of outer enamel, reduced subsurface visibility, and device algorithms optimized for detecting more advanced lesions [9, 41, 50, 51]. Despite low SE, both radiographic methods and IOSs demonstrated high SP (96%), suggesting a low risk of false positives when surfaces were classified as sound- an important advantage in early caries management, where overtreatment of non-cavitated lesions should be avoided.

Radiography also demonstrated poor SE (24%) for supragingival lesions, consistent with prior evidence that 30%-40% enamel demineralization is required before a lesion becomes radiographically visible [23, 52, 53]. In such cases, intraoral imaging devices may serve as a viable alternative, as this meta-analysis found no significant difference in diagnostic accuracy between IOSs/IOCs and radiographic methods [35, 36]. However, relatively few studies in this review specifically assessed supragingival lesions, limiting generalizability. This evidence gap underscores the need for further clinical research into the diagnostic performance of intraoral technologies for early-stage caries, where timely detection is critical for preventive intervention.

#### Dentin lesions

The meta-analysis identified clear performance differences across diagnostic tools for dentin caries. IOCs showed the highest pooled SE (75.6%) and strong SP (88.1%), likely due to the enhanced optical contrast of dentin lesions that favors fluorescence-based detection. IOCs were used in 21 of the 28 included studies, suggesting greater clinical acceptance compared to IOSs, which were featured in only 7 studies, reflecting the higher technical demands of IOSs, including the need for precise angulation and moisture control [41, 44].

Despite their strong overall performce, IOCs showed a tendency to over diagnose early lesions due to reliance on fixed fluorescence thresholds, which varied across systems (e.g., VistaProof, Spectra Caries Detection Aid, VistaCam), contributing to wide ranges of SE (78.6–92.3%) and SP (62.5–85.0%). In contrast, IOSs demonstrated lower pooled SE (50%) but higher SP (87.3%), reflecting a more conservative diagnostic profile. For example, the iTero Element 5D achieved excellent SP (96.1%) but showed poor SE (23.08%) for early enamel lesions, whereas TRIOS scanners, which incorporate fluorescence-based algorithms, achieved near-perfect SE for dentin lesions in certain studies [40], though with reduced reliability for enamel demineralization. These differences likely stem from the core detection technologies used: the iTero system relies on brightness-based algorithms that detect well-defined structural changes, prioritizing SP but often missing subtle demineralization [43]. In contrast, TRIOS scanners integrate fluorescence-based imaging, which enhances SE for subsurface dentin lesions but may increase false positives in early enamel detection due to light scatter and reflectance variability [54, 55].

These findings align with previous research [9, 41], showing both IOSs and IOCs perform best for deeper caries but require refinement for early enamel detection. However, the considerable heterogeneity observed in this subgroup suggests that the pooled performance metrics should not be interpreted uniformly across settings. Instead, diagnostic outcomes may vary depending on device type, user expertise, and the clinical environment. This reinforces the need for future studies to report diagnostic accuracy separately for key subgroups, such as lesion severity, tooth type, and imaging conditions, to improve interpretability and ensure findings are more applicable to real-world clinical settings.

#### Examiner training, calibration, and methodological factors affecting diagnostic reproducibility

Examiner-dependent variability significantly influenced diagnostic performance with IOSs and IOCs, while radiographic methods appeared less sensitive to operator-related differences. Of the 28 included studies, 16 involved two examiners, 7 used three or more, and 3 relied on a single examiner. Greater examiner proficiency was consistently linked to higher SE, largely reflecting the extent of structure training and calibration received prior to image interpretation.

For IOSs, examiner experience ranged from 2 to 5 years [10, 41], with many studies incorporating calibration using standardized datasets [15, 44]. Training often included image review sessions [6, 37], test scoring [23, 38, 39, 41], and expert-guided discussions [6, 10]. IOCs-related training varied more widely, from brief workshops [8] to multi-week calibration protocols [36]. Structured training methods, including peer discussions and expert validation, were associated with higher interrater reliability (IRR ≥ 0.85) [35, 36], while minimal training led to poorer agreement (IRR = 0.19–0.29) [6]. For example, Metzger et al. [10] showed that expert feedback improved IRR from 0.24 to 0.51, reinforcing the importance of comprehensive examiner calibration.

This meta-analysis also identified examiner-specific differences in diagnostic performance. Examiner 2 achieved higher SE with both IOSs (68.4%; 95% CI: 0.54–0.80) and IOCs (82.7%; 95% CI: 0.73–0.90) compared to examiner 1 (50%; 95% CI: 0.47–0.53 for both). However, this improvement came with greater diagnostic variability: Tau² values (SE = 0.41 for IOS, SP = 1.79 for IOS, and SE = 0.87 for IOC) were higher for examiner 2, whereas examiner 1 showed more stable, though less sensitive, performance (Tau² = 0.1 for both SE and SP across devices). This may reflect a trade-off between diagnostic vigilance and consistency—experienced or well-trained examiners may be more adept at identifying subtle or borderline lesions, but their increased subjectivity can lead to greater variation in interpretation across studies [8, 10, 35] (Supplementary Fig. 1).

Blinding emerged as another key factor influencing reproducibility. Among the 28 studies, 23 employed blinding; those that did not generally reported lower IRR values (0.39–0.44) [5, 47–49]. In contrast, blinded studies, such as Edrees et al. [36] and Salama et al. [35], achieved near-perfect agreement (IRR ≥ 0.85). Interestingly, one non-blinded study [22] reported a relatively higher IRR (0.48), suggesting that rigorous training can partially mitigate the absence of blinding. Nevertheless, variability remained most pronounced among less experienced or inadequately trained examiners [6, 9, 15, 36, 44]. These findings highlight that diagnostic accuracy with intraoral imaging tools depends not only on the device but also significantly on examiner-related factors. Even with training and blinding, variability in interpreting early or ambiguous lesions persists, limiting the generalizability and reproducibility of pooled results. To reduce this variability, future studies should standardize training protocols, establish minimum calibration requirements, and report examiner metrics such as experience and IRR. Future integration of AI-assisted scoring tools may also help reduce subjectivity and improve consistency in image-based caries diagnosis.

Given the substantial heterogeneity observed, particularly in dentin and enamel lesion detection, this study considered the utility of meta-regression. However, this approach was not feasible due to a limited number of studies per subgroup and inconsistent reporting of key effect modifiers such as lesion depth, tooth type, examiner calibration, and device-specific variables. Since meta-regression requires a sufficient number of studies (minimum 10 studies) per covariate [56] and consistent reporting across studies to reliably identify sources of heterogeneity, applying it under the current conditions could result in unstable or misleading estimates [57]. Future systematic reviews should consider applying meta-regression to further explore sources of heterogeneity and enhance the evaluation of diagnostic accuracy across different diagnostic modalities.

Beyond statistical approaches, future studies should also explore next-generation diagnostic technologies. Emerging technologies such as photoacoustic imaging (PAI) and terahertz (THz) imaging have been proposed as non-ionizing, high-resolution approaches for assessing subsurface enamel and dentin structures [58, 59]. However, their clinical application is limited by technical challenges including device size, interference from saliva and soft tissues, and resolution constraints [60, 61]. Translational studies are needed to assess their feasibility and diagnostic performance in real-world dental settings. Lastly, while artificial intelligence (AI) and machine learning were beyond the scope of this analysis, their integration with intraoral imaging systems may offer automated real-time diagnostics while reducing examiner variability [62]. Future research should focus on developing and validating such systems using large, diverse datasets to enhance reproducibility and assist clinical decision-making.

This systematic review and meta-analysis have several limitations:

1. Substantial heterogeneity existed across studies in diagnostic protocols, lesion types, device brands, examiner expertise, and reference standards, limiting the feasibility of refined subgroup analyses.
2. Incomplete reporting of diagnostic data in some studies required recalculations based on SE and SP values, which may have introduced minor discrepancies.
3. Only 16 of the 28 studies were eligible for meta-analysis due to data limitations, reducing generalizability.
4. The exclusion of laser fluorescence and other non-imaging diagnostic tools may limit the broader applicability of findings.
5. The analysis was limited to diagnostic accuracy metrics and did not assess real-time clinical performance or patient-centered outcomes such as overtreatment or lesion progression.
6. Studies were restricted to English language publications, and examiner training and calibration were inconsistently reported, limiting the ability to quantify their direct impact on diagnostic accuracy.

## Conclusions

This review found that intraoral scanners and cameras demonstrated moderate diagnostic accuracy for caries detection, with higher sensitivity for occlusal and dentin lesions, while radiography consistently achieved higher specificity, particularly for advanced caries. Diagnostic accuracy varied by lesion types, with supragingival and early enamel lesions exhibiting consistently low sensitivity across all methods. Examiner-dependent factors, such as experience, training, and calibration, significantly influenced accuracy and contributed to inter-study variability. Additionally, the absence of standardized diagnostic protocols and inconsistent reference standards limited the comparability of results across studies.

## Supplementary information

PRISMA Checklist

Supplementary Information

## Data Availability

All data extracted and analyzed in this study, along with the code scripts used for the statistical analysis, are available in the supplementary file submitted with the manuscript.
