# Supplementary material for: Comparison of the accuracy of intraoral scanners, intraoral cameras, radiographs, and histological methods for the diagnosis of dental caries: a systematic review and meta-analysis
Source: BDJ Open. 2025 Oct 13;11:82. doi: 10.1038/s41405-025-00345-5 (PMC12518662; doi:10.1038/s41405-025-00345-5)
Supplement: Supplementary file 2 — Supplementary Information [file 41405_2025_345_MOESM2_ESM.pdf]

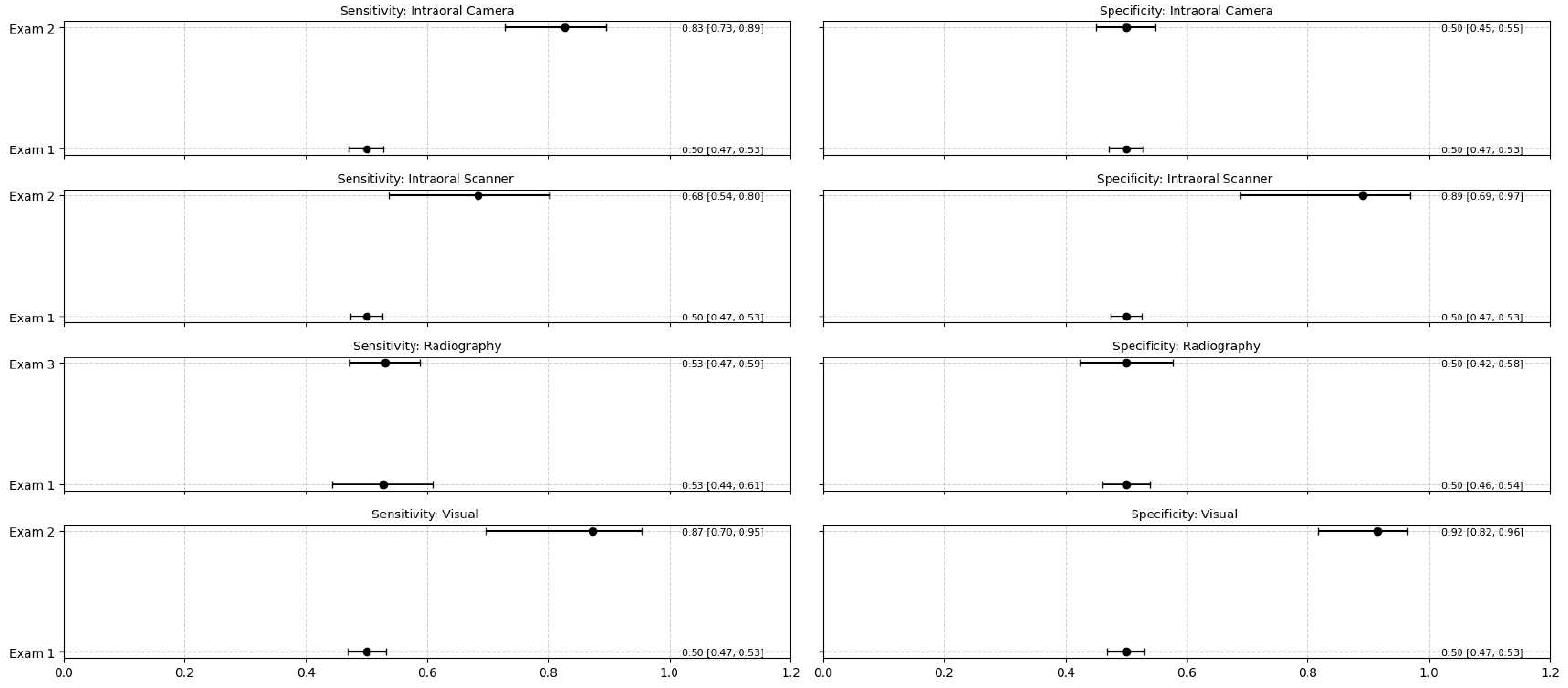
Sensitivity: Intraoral Camera
Exam 2
Exam 1
0.83 [0.73, 0.89]
0.50 [0.47, 0.53]
Specificity: Intraoral Camera
0.50 [0.45, 0.55]
0.50 [0.47, 0.53]
Sensitivity: Intraoral Scanner
0.68 [0.54, 0.80]
0.50 [0.47, 0.53]
Specificity: Intraoral Scanner
0.89 [0.69, 0.97]
0.50 [0.47, 0.53]
Sensitivity: Radiography
Exam 3
0.53 [0.47, 0.59]
0.53 [0.44, 0.61]
Specificity: Radiography
0.50 [0.42, 0.58]
0.50 [0.46, 0.54]
Sensitivity: Visual
0.87 [0.70, 0.95]
0.50 [0.47, 0.53]
Specificity: Visual
0.92 [0.82, 0.96]
0.50 [0.47, 0.53]
0.0
0.2
0.4
0.6
0.8
1.0
1.2

Supplementary Table 1: Keyword-based search strategies used across databases for each review objective

Database	Objective	Search Strings	Articles obtained per objective	Total number of articles per database
**Scopus**	Objective 1	scanner OR “dental scanner*” OR “intraoral scanner*” OR “near-infrared light transillumination” AND “dental radiograph*” OR “dental X-ray” OR “dental imaging” OR “X-ray picture” OR “oral radiograph*” OR “oral X-ray” OR “oral imaging” OR “teeth radiograph” OR “teeth X-ray” OR “teeth imaging” AND “tooth decay” OR cavit* OR “carious lesion*” OR “dental decay” OR “decayed tooth” OR “decayed teeth” OR “demineral i?e* teeth” OR “demineral i?e* tooth”	164	718
	Objective 2	scanner OR “dental scanner*” OR “intraoral scanner*” OR “near-infrared light transillumination” AND histologic* OR histopathological OR microscopic OR excisional OR “teeth cross-section” OR “tooth cross-section” AND “tooth decay” OR cavit* OR “carious lesion*” OR “dental decay” OR “decayed tooth” OR “decayed teeth” OR “demineral i?e* teeth” OR “demineral i?e* tooth”	258	
	Objective 3	“intraoral photograph*” OR “dental photograph*” OR “intraoral camera*” OR “intra-oral photograph*” OR “intra-oral camera*” OR camera* OR “intraoral photo*” OR “intra-oral photo” AND “dental radiograph” OR “dental X-ray” OR “dental imaging” OR “X-ray picture” OR “oral radiograph*” OR “oral X-ray” OR “oral imaging” OR “teeth radiograph” OR “teeth X-ray” OR “teeth imaging” AND “tooth decay” OR cavit* OR “carious lesion*” OR “dental decay” OR “decayed tooth” OR “decayed teeth” OR “demineral i?e* teeth” OR “demineral i?e* tooth”	66	
	Objective 4	“intraoral photograph*” OR “dental photograph*” OR “intraoral camera*” OR “intra-oral photograph*” OR “intra-oral camera*” OR camera* OR “intraoral photo*” OR “intra-oral photo” AND histologic* OR	230	

		histopathological OR microscopic OR excisional OR “teeth cross-section” OR “tooth cross-section” AND "tooth decay" OR cavit* OR “carious lesion*” OR “dental decay” OR “decayed tooth” OR “decayed teeth” OR “demineralі?e* teeth” OR “demineralі?e* tooth”		
**Web of Science**	Objective 1	scanner OR “dental scanner*” OR “intraoral scanner*” OR “near-infrared light transillumination” AND “dental radiograph*” OR “dental X-ray” OR “dental imaging” OR “X-ray picture” OR “oral radiograph*” OR “oral X-ray” OR “oral imaging” OR “teeth radiograph” OR “teeth X-ray” OR “teeth imaging” AND “tooth decay” OR cavit* OR “carious lesion*” OR “dental decay” OR “decayed tooth” OR “decayed teeth” OR “demineralі?e* teeth” OR “demineralі?e* tooth”	11	69
	Objective 2	scanner OR “dental scanner*” OR “intraoral scanner*” OR “near-infrared light transillumination” AND histologic* OR histopathological OR microscopic OR excisional OR “teeth cross-section” OR “tooth cross-section” AND “tooth decay” OR cavit* OR “carious lesion*” OR “dental decay” OR “decayed tooth” OR “decayed teeth” OR “demineralі?e* teeth” OR “demineralі?e* tooth”	26	
	Objective 3	“intraoral photograph*” OR “dental photograph*” OR “intraoral camera*” OR “intra-oral photograph*” OR “intra-oral camera*” OR camera* OR “intraoral photo*” OR “intra-oral photo” AND “dental radiograph” OR “dental X-ray” OR “dental imaging” OR “X-ray picture” OR “oral radiograph*” OR “oral X-ray” OR “oral imaging” OR “teeth radiograph” OR “teeth X-ray” OR “teeth imaging” AND “tooth decay” OR cavit* OR “carious lesion*” OR “dental decay” OR “decayed tooth” OR “decayed teeth” OR “demineralі?e* teeth” OR “demineralі?e* tooth”	5	
	Objective 4	“intraoral photograph*” OR “dental photograph*” OR “intraoral camera*” OR “intra-oral photograph*” OR “intra-oral camera*” OR camera* OR “intraoral photo*”	27	

		OR “intra-oral photo” AND histologic* OR histopathological OR microscopic OR excisional OR “teeth cross-section” OR “tooth cross-section” AND "tooth decay" OR cavit* OR “carious lesion*” OR “dental decay” OR “decayed tooth” OR “decayed teeth” OR “deminerali?e* teeth” OR “deminerali?e* tooth”		
**PubMed**	Objective 1	((“tomography Scanners, X-Ray Computed”[mh] OR scanner[tiab] OR dental scanner*[tiab] OR intraoral scanner*[tiab] OR near-infrared light transillumination[tiab]) AND (“radiography, dental”[mh] OR dental radiograph*[tiab] OR dental X-ray[tiab] OR dental imaging[tiab] OR X-ray picture[tiab] OR oral radiograph*[tiab] OR oral X-ray[tiab] OR oral imaging[tiab] OR teeth radiograph[tiab] OR teeth X-ray[tiab] OR teeth imaging[tiab]) AND (“dental caries”[mh] OR tooth decay[tiab] OR cavit*[tiab] OR carious lesion*[tiab] OR dental decay[tiab] OR decayed tooth[tiab] OR decayed teeth[tiab] OR demineralize* teeth[tiab] OR demineralize* tooth[tiab]))	72	322
	Objective 2	((“tomography Scanners, X-Ray Computed”[mh] OR scanner [tiab] OR dental scanner*[tiab] OR intraoral scanner*[tiab] OR near-infrared light transillumination[tiab]) AND (“histological techniques”[mh] OR histologic*[tiab] OR histopathological[tiab] OR microscopic[tiab] OR excisional [tiab] OR teeth cross-section[tiab] OR tooth cross-section[tiab]) AND ("dental caries"[mh] OR tooth decay[tiab] OR cavit*[tiab] OR carious lesion*[tiab] OR dental decay[tiab] OR decayed tooth[tiab] OR decayed teeth[tiab] OR demineralize* teeth[tiab] OR demineralize* tooth[tiab]))	43	
	Objective 3	(("photography, dental"[mh] OR intraoral photograph*[tiab] OR dental photograph*[tiab] OR intraoral camera*[tiab] OR intra-oral photograph*[tiab] OR intra-oral camera*[tiab] OR camera*[tiab] OR intraoral photo*[tiab] OR intra-oral photo[tiab]) AND	102	

		("radiography, dental"[mh] OR dental radiograph* [tiab] OR dental X-ray [tiab] OR dental imaging [tiab] OR X-ray picture [tiab] OR oral radiograph* [tiab] OR oral X-ray [tiab] OR oral imaging [tiab] OR teeth radiograph [tiab] OR teeth X-ray [tiab] OR teeth imaging [tiab]) AND ("dental caries"[mh] OR tooth decay [tiab] OR cavit* [tiab] OR carious lesion* [tiab] OR dental decay [tiab] OR decayed tooth [tiab] OR decayed teeth [tiab] OR demineralize* teeth [tiab] OR demineralize* tooth [tiab]))		
	Objective 4	(("photography, dental"[mh] OR intraoral photograph*[tiab] OR dental photograph*[tiab] OR intraoral camera*[tiab] OR intra-oral photograph*[tiab] OR intra-oral camera*[tiab] OR camera*[tiab] OR intraoral photo*[tiab] OR intra-oral photo[tiab]) AND (“histological techniques”[mh] OR histologic*[tiab] OR histopathological[tiab] OR microscopic[tiab] OR excisional [tiab]OR teeth cross-section[tiab] OR tooth cross-section[tiab]) AND ("dental caries"[mh] OR tooth decay [tiab] OR cavit* [tiab] OR carious lesion* [tiab] OR dental decay [tiab] OR decayed tooth [tiab] OR decayed teeth [tiab] OR demineralize* teeth [tiab] OR demineralize* tooth [tiab]))	105	
**Cochrane Library**	Objective 1	[mh “tomography Scanners, X-Ray Computed”] OR scanner:ti,ab OR (dental NEXT scanner*):ti,ab OR (intraoral NEXT scanner*):ti,ab OR (near NEXT infrared NEXT light NEXT transillumination):ti,ab AND [mh “radiography, dental”] OR (dental NEXT radiograph*):ti,ab OR dental X-ray:ti,ab OR dental imaging:ti,ab OR X-ray picture:ti,ab OR (oral NEXT radiograph*):ti,ab OR oral X-ray:ti,ab OR oral imaging:ti,ab OR teeth radiograph:ti,ab OR teeth X-ray:ti,ab OR teeth imaging:ti,ab AND [mh “dental caries”] OR tooth decay:ti,ab OR cavit*:ti,ab OR (carious NEXT lesion*):ti,ab OR dental decay:ti,ab OR decayed tooth:ti,ab OR decayed teeth:ti,ab OR (teeth	13	38

		NEXT demineralize*):ti,ab OR (tooth NEXT demineralize*):ti,ab		
	Objective 2	[mh “tomography Scanners, X-Ray Computed”] OR scanner:ti,ab OR (dental NEXT scanner*):ti,ab OR (intraoral NEXT scanner*):ti,ab OR (near NEXT infrared NEXT light NEXT transillumination):ti,ab AND [mh “histological techniques”] OR histologic*:ti,ab OR histopathological:ti,ab OR microscopic:ti,ab OR excisional:ti,ab OR teeth cross-section:ti,ab OR tooth cross-section:ti,ab AND [mh “dental caries”] OR tooth decay:ti,ab OR cavit*:ti,ab OR (carious NEXT lesion*):ti,ab OR dental decay:ti,ab OR decayed tooth:ti,ab OR decayed teeth:ti,ab OR (teeth NEXT demineralize*):ti,ab OR (tooth NEXT demineralize*):ti,ab	1	
	Objective 3	[mh "photography, dental"] OR (intraoral NEXT photograph*):ti,ab OR (dental NEXT photograph*):ti,ab OR (intraoral NEXT camera*):ti,ab OR (intra-oral NEXT photograph*):ti,ab OR (intra-oral NEXT camera*):ti,ab OR camera*:ti,ab OR (intraoral NEXT photo*):ti,ab OR intra-oral photo:ti,ab AND [mh “radiography, dental”] OR (dental NEXT radiograph*):ti,ab OR dental X-ray:ti,ab OR dental imaging:ti,ab OR X-ray picture:ti,ab OR (oral NEXT radiograph*):ti,ab OR oral X-ray:ti,ab OR oral imaging:ti,ab OR teeth radiograph:ti,ab OR teeth X-ray:ti,ab OR teeth imaging:ti,ab AND [mh “dental caries”] OR tooth decay:ti,ab OR cavit*:ti,ab OR (carious NEXT lesion*):ti,ab OR dental decay:ti,ab OR decayed tooth:ti,ab OR decayed teeth:ti,ab OR (teeth NEXT demineralize*):ti,ab OR (tooth NEXT demineralize*):ti,ab	15	
	Objective 4	[mh "photography, dental"] OR (intraoral NEXT photograph*):ti,ab OR (dental NEXT photograph*):ti,ab OR (intraoral NEXT camera*):ti,ab OR (intra-oral NEXT photograph*):ti,ab OR (intra-oral NEXT	9	

		camera*):ti,ab OR camera*:ti,ab OR (intraoral NEXT photo*):ti,ab OR intra-oral photo:ti,ab AND [mh "histological techniques"] OR histologic*:ti,ab OR histopathological:ti,ab OR microscopic:ti,ab OR excisional:ti,ab OR teeth cross-section:ti,ab OR tooth cross-section:ti,ab AND [mh "dental caries"] OR tooth decay:ti,ab OR cavit*:ti,ab OR (carious NEXT lesion*):ti,ab OR dental decay:ti,ab OR decayed tooth:ti,ab OR decayed teeth:ti,ab OR (teeth NEXT demineralize*):ti,ab OR (tooth NEXT demineralize*):ti,ab		
**Dentistry & Oral Sciences Source**	Objective 1	(DE "TOMOGRAPHIC scanners" OR TI "scanner" OR AB "scanner" OR TI "dental scanner*" OR AB "dental scanner*" OR TI "intraoral scanner*" OR AB "intraoral scanner*" OR TI "near-infrared light transillumination" OR AB "near-infrared light transillumination") AND ((DE "DENTAL radiography" OR DE "ORAL radiography") OR TI "dental radiograph*" OR AB "dental radiograph*" OR TI "dental X-ray" OR AB "dental X-ray" OR TI "dental imaging" OR AB "dental imaging" OR TI "X-ray picture" OR AB "X-ray picture" OR TI "oral X-ray" OR AB "oral X-ray" OR TI "oral imaging" OR AB "oral imaging" OR TI "teeth radiograph" OR AB "teeth radiograph" OR TI "teeth X-ray" OR OR AB "teeth X-ray" OR TI "teeth imaging" OR AB "teeth imaging") AND ((DE "DENTAL caries" OR DE "TOOTH demineralization" OR DE "DIAGNOSIS of dental caries") OR TI "tooth decay" OR AB "tooth decay" OR TI cavit* OR AB cavit* OR TI "carious lesion*" OR AB "carious lesion*" OR TI "dental decay" OR AB "dental decay" OR TI "decayed tooth" OR AB "decayed tooth" OR TI "decayed teeth" OR AB "decayed teeth" OR TI "demineral?e* teeth" OR AB "demineral?e* teeth" OR TI "demineral?e* tooth" OR AB "demineral?e* tooth")	14	60

	Objective 2	(DE “TOMOGRAPHIC scanners” OR TI “scanner” OR AB “scanner” OR TI “dental scanner*” OR AB “dental scanner*” OR TI “intraoral scanner*” OR AB “intraoral scanner*” OR TI “near-infrared light transillumination” OR AB “near-infrared light transillumination”) AND (DE “HISTOLOGICAL techniques” OR TI histologic* OR AB histologic* OR TI histopathological OR AB histopathological OR TI microscopic OR AB microscopic OR TI excisional OR AB excisional OR TI “teeth cross-section” OR AB “teeth cross-section” OR TI “tooth cross-section” OR AB “tooth cross-section”) AND ((DE “DENTAL caries" OR DE “TOOTH demineralization” OR DE “DIAGNOSIS of dental caries”) OR TI “tooth decay” OR AB “tooth decay” OR TI cavit* OR AB cavit* OR TI “carious lesion*” OR AB “carious lesion*” OR TI “dental decay” OR AB “dental decay” OR TI “decayed tooth” OR AB “decayed tooth” OR TI “decayed teeth” OR AB “decayed teeth” OR TI “demineráli?e* teeth” OR AB “demineráli?e* teeth” OR TI “demineráli?e* tooth” OR AB “demineráli?e* tooth”)	15	
	Objective 3	((DE "DENTAL photography") OR (DE "MEDICAL photography") OR TI “intraoral photograph*” OR AB “intraoral photograph*” OR TI “dental photograph*” OR AB “dental photograph*”OR TI “intraoral camera*” OR AB “intraoral camera*” OR TI intra-oral photograph* OR AB “intra-oral photograph*” OR TI “intra-oral camera*” OR AB “intra-oral camera*” OR TI camera* OR AB camera* OR TI intraoral photo* OR AB intraoral photo*OR TI “intra-oral photo” OR AB “intra-oral photo”) AND ((DE “DENTAL radiography" OR DE "ORAL radiography") OR TI “dental radiograph*” OR AB “dental radiograph*” OR TI “dental X-ray” OR AB “dental X-ray” OR TI “dental imaging” OR AB “dental imaging” OR TI “X-ray picture” OR AB “X-ray picture” OR TI “oral X-ray” OR	18	

		AB “oral X-ray” OR TI “oral imaging” OR AB “oral imaging” OR TI “teeth radiograph” OR AB “teeth radiograph” OR TI “teeth X-ray” OR OR AB “teeth X-ray” OR TI “teeth imaging” OR AB “teeth imaging”) AND ((DE “DENTAL caries" OR DE “TOOTH demineralization” OR DE “DIAGNOSIS of dental caries”) OR TI “tooth decay” OR AB “tooth decay” OR TI cavit* OR AB cavit* OR TI “carious lesion*” OR AB “carious lesion*” OR TI “dental decay” OR AB “dental decay” OR TI “decayed tooth” OR AB “decayed tooth” OR TI “decayed teeth” OR AB “decayed teeth” OR TI “deminerali?e* teeth” OR AB “deminerali?e* teeth” OR TI “deminerali?e* tooth” OR AB “deminerali?e* tooth”)		
	Objective 4	((DE "DENTAL photography") OR (DE "MEDICAL photography") OR TI “intraoral photograph*” OR AB “intraoral photograph*” OR TI “dental photograph*” OR AB “dental photograph*”OR TI “intraoral camera*” OR AB “intraoral camera*” OR TI intra-oral photograph* OR AB “intra-oral photograph*” OR TI “intra-oral camera*” OR AB “intra-oral camera*” OR TI camera* OR AB camera* OR TI intraoral photo* OR AB intraoral photo*OR TI “intra-oral photo” OR AB “intra-oral photo”) AND (DE “HISTOLOGICAL techniques” OR TI histologic* OR AB histologic* OR TI histopathological OR AB histopathological OR TI microscopic OR AB microscopic OR TI excisional OR AB excisional OR TI “teeth cross-section” OR AB “teeth cross-section” OR TI “tooth cross-section” OR AB “tooth cross-section”) AND ((DE “DENTAL caries" OR DE “TOOTH demineralization” OR DE “DIAGNOSIS of dental caries”) OR TI “tooth decay” OR AB “tooth decay” OR TI cavit* OR AB cavit* OR TI “carious lesion*” OR AB “carious lesion*” OR TI “dental decay” OR AB “dental decay” OR TI “decayed tooth” OR AB “decayed tooth” OR TI “decayed teeth”	13	

		OR AB “decayed teeth” OR TI “demineralі?e* teeth” OR AB “demineralі?e* teeth” OR TI “demineralі?e* tooth” OR AB “demineralі?e* tooth”)		

Supplementary Table 2: Scoring criteria used for quality assessment of studies included in the meta-analysis

No	Criterion	Decision Role
**Selection (Maximum 8 points)**		
1.	Representativeness of Exposed Cohort (Intraoral Devices)	Score 2: Comprehensive details of the intraoral devices, including device type, specifications, and calibration protocols.<br>Score 1: Partial description of the intraoral device, with basic details provided (e.g., device name and type) but lacking specifics on calibration or usage settings.<br>Score 0: No description or insufficient details of the intraoral device provided.
2.	Selection of the Non-Exposed Cohort (Traditional Methods such as visual and radiography)	Score 1: Control group was clearly defined using standard traditional methods (i.e., visual, or radiography)<br>Score 0: Poorly defined control group or use of non-standard method
3.	Ascertainment of Diagnostic Tool (Exposure)	Score 1: Comprehensive description of device usage, settings, calibration, and environmental conditions<br>Score 0: Inadequate description of device usage
4.	Baseline status of dental caries	Score 2: Comprehensive baseline assessment, including caries-free teeth, severity (e.g., enamel or dentin caries), and location documentation (e.g., occlusal or proximal caries)<br>Score 1: Partial baseline assessment (e.g., severity or location reported, but not both).<br>Score 0: Unclear or missing baseline status of dental caries.
**Comparability (Maximum 2 points)**		
1.	Comparability of variables based on confounding factors	Score 2: Comprehensive control (e.g., examiner-dependent variabilities, dental and light variabilities of the devices)<br>Score 1: Partial control (e.g., only one confounding factor described).

		Score 0: No mention of confounding factors.
**Outcome (Maximum 3 points)**		
1.	Assessment of Outcome (Diagnostic Accuracy)	Score 3: Clear and independent reference standard used (e.g., histology or validated radiographic method), with diagnostic metrics (e.g., Sensitivity, Specificity).<br>Score 2: Reference standard used, but missing one key metric.<br>Score 1: Outcome measures unclear, but reference standard mentioned.<br>Score 0: Neither reference standard nor diagnostic metrics provided.

Supplementary Table 3: Reasons for exclusion of articles from this systematic review

No	Author name & year	Title	Reasons for Exclusion
1.	Michou, Stavroula, 2024	Occlusal caries detection and monitoring using a 3D intraoral scanner system. An in vivo assessment	The study lacks essential diagnostic performance metrics such as sensitivity and specificity, which are crucial for this systematic review.
2.	Sridhar, Nekkanti, 2023	Diagnosis of occlusal enamel caries using near-infrared light transillumination.	Full paper not found.
3.	Moro, B.L.P., 2023	Secondary Caries Detection and Treatment Decision according to Two Criteria and the Impact of a Three-Dimensional Intraoral Scanner on Gap Evaluation	The study primarily focuses on gap evaluation using 3D intraoral scanners rather than caries detection by using imaging methods.
4.	Caceda, J.H., 2023	Sensitivity and specificity of the ICDAS II system and bitewing radiographs for detecting occlusal caries using the Spectra™ caries detection system as the reference test in children	The study used a laser fluorescence tool, the Spectra™ Caries Detection System (SCDS), which provides numeric values rather than 2D or 3D images, making it inconsistent with the imaging-based methods required for this review.
5.	Dhanavel, C., 2023	Evaluation of Reliability and Validity of Occlusal Caries Detection by Direct Visual, Indirect Visual, and Fluorescence Camera Using ICDAS II (Codes 0, 1, and 2): An In Vivo Study	The study did not use any reference standard, such as radiographic or histological methods, which are required for inclusion in the systematic review.
6.	Sobral, Ana Paula Taboada, 2022	Evaluation of different methods for the diagnosis of primary caries lesions: Study protocol for a randomized controlled clinical trial	The study is a protocol and lacks essential diagnostic performance metrics such as sensitivity and specificity, which are crucial for this systematic review.

7.	Mazur, M., 2020	Correlation between Vista Cam, ICDAS-II, x-ray bitewings and cavity extent after lesion excavation: an in vivo pilot study.	Full paper not found.
8.	De Zutter. M et al. 2020	In vivo correlation of near-infrared transillumination and visual inspection with bitewing radiography for the detection of interproximal caries in permanent and primary teeth	The study lacks essential diagnostic performance metrics such as sensitivity, specificity, AUC, and predictive values, which are crucial for this systematic review.
9.	Kühnisch, Jan, 2019	Evaluation of detecting proximal caries in posterior teeth via visual inspection, digital bitewing radiography and near-infrared light transillumination.	Full paper not found.
10.	Melo, Maria., 2019	Combined Near-Infrared Light Transillumination and Direct Digital Radiography Increases Diagnostic Accuracy in Approximal Caries	The study reported only sensitivity and lacked other key metrics, especially specificity, which is crucial for this systematic review.
11.	Schaefer, G., 2018	Evaluation of occlusal caries detection and assessment by visual inspection, digital bitewing radiography and near-infrared light transillumination	The study lacks essential diagnostic performance metrics, such as sensitivity, specificity, and reference standards, such as radiographic or histological methods, which are crucial for this systematic review.
12.	Lara-Capi, 2017	Digital transillumination in caries detection versus radiographic and clinical methods: An in-vivo study	The study lacks essential diagnostic performance metrics such as sensitivity, specificity, AUC, and predictive values, which are crucial for this systematic review.
13.	Abdelaziz, M., 2017	Near infrared transillumination compared with radiography to detect and monitor proximal caries: A clinical retrospective study	The study lacks essential diagnostic performance metrics such as sensitivity, specificity, AUC, and predictive values, which are crucial for this systematic review.
14.	Berg, S.C., 2017	A clinical study comparing digital radiography and near-infrared transillumination in caries detection	The study lacks essential diagnostic performance metrics, such as sensitivity, specificity, which are crucial for this systematic review.

15.	Söchtig, 2014	Caries detection and diagnostics with near-infrared light transillumination: Clinical experiences	The study lacks essential diagnostic performance metrics such as sensitivity, specificity, AUC, and predictive values, which are crucial for this systematic review.
16.	Larentis, N.L., 2014	Measures obtained from images acquired by a stereomicroscope and a desktop scanner	The study did not use any recognized caries classification systems or report any key diagnostic metrics such as sensitivity and specificity, which are crucial for this systematic review.
17.	Jablonski-Momeni, 2012	Use of ICDAS-II, fluorescence-based methods, and radiography in detection and treatment decision of occlusal caries lesions: an in vitro study	The study uses a laser fluorescence tool, the DIAGNOdent pen, which provides numeric values rather than 2D or 3D images, making it inconsistent with the imaging-based methods required for this systematic review.
18.	Mirska-Mietek, 2010	[Diagnosis of caries on approximal surfaces of permanent teeth].	Full paper not found.
19.	Terrer, E., 2009	A new concept in restorative dentistry: Light-induced fluorescence evaluator for diagnosis and treatment: Part 1 - diagnosis and treatment of initial occlusal caries	The study lacks essential diagnostic performance metrics such as sensitivity, specificity, and reference standards, such as radiographic or histological methods, which are crucial for this systematic review.
20.	Bin-Shuwaish, 2008	The correlation of DIFOTI to clinical and radiographic images in Class II carious lesions.	The study reported only sensitivity and lacked other key metrics, especially specificity. Additionally, the study did not use any recognized caries classification systems.
21.	Forgie AH. 2003	The assessment of an intra-oral video camera as an aid to occlusal caries detection	The study uses a video to detect the carious lesions. No images were generated from the video.
22.	Bjorndal, L., 1999	A computerized analysis of the relation between the occlusal enamel caries lesion and the demineralized dentin	The study lacks essential diagnostic performance metrics such as sensitivity, specificity, and reference

			standards, such as radiographic or histological methods, which are crucial for this systematic review.

Supplementary Table 4: Data related to variables affecting the diagnostic accuracy of intraoral scanners and cameras, including blinding status, inter- and intra-rater reliability, device-specific factors (e.g., lighting conditions, calibration), study-level limitations, and reproducibility considerations

Author (Y)	Blinding	Observer's variability (Inter/ Intra-rater reliability)	Device Dependent Variabilities	Confounding Factors	Study Limitations	Reproducibility/other comments
Patel J., 2024	Examiners were unaware of each other's findings and the reference standard.	• **ICDAS-II (Visual)** Inter-rater reliability = 0.88 (good agreement)<br>• **Intraoral camera (DIAGNOcam)** Inter-rater reliability = 0.64 (moderate agreement)<br>• **Digital Radiography (BWR)** Inter-rater reliability = 0.80 (good agreement)	• **ICDAS-II (Visual)** The examiners photograph the images, using a Canon EOS 70D DSLR camera, a macro lens, and a ring flash. The settings included a shutter speed of 1/200 s, an aperture of f/22, and ISO 200, ensuring consistent lighting across all images.<br>• **Intraoral Camera (DIAGNOcam)** Teeth surfaces were dried thoroughly to reduce any moisture before capturing images. The device was positioned carefully to interproximal surfaces, with small and large tips used based on patient comfort and tooth size.<br>• **Radiograph** Heliodent Plus X-ray unit was used with parameters such as 70kV, 7mA, and 0.16–0.20s.<br>• **Image Viewing** The lighting condition of the room and the screen on which they have viewed the images are unclear for every method.	• The study mentions that image quality was a significant factor affecting the accuracy of NIDIT. Poor-quality images, due to issues like overexposure or improper positioning, reduced the diagnostic reliability of the tool.<br>• There were challenges in capturing stable images with the DIAGNOcam due to a latency between pressing the capture button and acquiring the image, which required multiple attempts to obtain a clear image.<br>• The study mentions that the direction of enamel rods in primary teeth differs from those in permanent teeth, which might influence how well DIAGNOcam detects caries. This anatomical difference could affect the interpretation of DIAGNOcam results compared to permanent teeth.	• As a retrospective audit, the study relied on existing clinical images and data, which limited control over image quality and consistency, especially for DIAGNOcam.<br>• The study used radiography as the reference standard instead of histology, which limited the ability to validate findings against a gold-standard measure of caries.<br>• Observers had limited training with DIAGNOcam, as it is a relatively new tool. This may have influenced variability in scoring and interpretation.	The study reported inter-rater reliability for each diagnostic method, indicating that radiography and visual photographic examination showed better reliability than DIAGNOcam, which suggests that both techniques were more consistent compared to DIAGNOcam.
Cuenin K. et al., 2024	The graders were blinded to clinical outcomes and the diagnostic results from other imaging methods while interpreting the intraoral scanner and radiographic images.	• **Intraoral Scanner (iTero Element 5D)** **Inter-rater reliability** Grade 0 (sound surface) = 0.29 (fair reliability), Grade 1 (enamel lesion) = 0.01 (poor reliability), Grade 2 (enamel lesion reaching DEJ) = 0.11(poor	• Radiographs were viewed on MiPACs software (Medicor Imaging), and 3D scans were viewed on Myitero software.	• There were concerns about the quality of images obtained during the scanning process, which may have impacted the results. Non-ideal radiographs or scans may lead to errors in caries detection.	• The examiners had limited experience with the intraoral scanner (iTero Element 5D) technology, potentially affecting diagnostic accuracy and consistency. More experienced clinicians might improve the reliability of NIRI interpretations.<br>• The smaller sample size for primary teeth (63 surfaces) limited the generalizability of	The study emphasized that iTero Element 5D performance heavily depends on user training and familiarity, which is a critical factor for clinical reproducibility. Despite low reproducibility, the non-ionizing nature of the scanner remains a significant advantage, particularly for pediatric populations.

		reliability), Grade 3 (outer third dentin) = 0.16 (poor reliability), Grade 4 (middle third dentin) = 0.37 (fair reliability), Combined = 0.19 (poor reliability)<br>• **Digital Radiograph (BWR)**<br>Grade 0 (sound surface) = 0.33 (fair reliability),<br>Grade 1 (enamel lesion) = 0.05 (poor reliability),<br>Grade 2 (enamel lesion reaching DEJ) = 0.25(fair reliability), Grade 3 (outer third dentin) = 0.19 (poor reliability), Grade 4 (middle third dentin) = 0.19 (poor reliability), Combined = 0.18 (poor reliability)			the findings for this group compared to permanent teeth.<br>• The study lacked a definitive gold standard for caries detection, such as histological analysis, which could confirm the actual lesion depth and validate imaging findings.	
Saffarpour A., et al. 2023	A separate researcher who was responsible for histological assessment was blinded to the results of the other diagnostic methods	• Not specified	• **ICDAS-II (Visual)**<br>No specific details are provided<br>• **Intraoral Camera (VistaCam)**<br>The camera used a 406nm wavelength to record the data. If indicated, a yellow-green<br>light (510 nm) then it was considered a healthy tooth, and a red light (680 nm) a carious tooth<br>surface. Later, DBSWIN software was used to convert the data.<br>• **Digital radiograph**<br>For radiographs, 70 kVp voltage, 8 mm focal point, a filter, and an exposure time of 16 seconds were used.<br>• **Histological**	• Enamel cracks could lead to false-positive results in VistaCam imaging because these cracks appear as bright areas, potentially mimicking caries.	• The study was conducted in vitro, meaning the findings might not fully translate to clinical (in vivo) settings due to the controlled environment<br>• The study lacked real-time clinical variables, such as patient movement and natural oral conditions, which could influence the accuracy of diagnostic tools in a clinical setting	Higher repeatability between ICDAS-II and radiography suggests these methods were more consistent when used together, compared to the other pairings, such as ICDAS-II with VistaCam and radiography with VistaCam.

			A diamond saw and a cooling Mecatome T210 were used to section the teeth mesiodistally with a 1mm thickness. 10X Olympus SZ<br>A 60 stereomicroscope was used to examine the samples.			
Ntovas P., et al., 2023	The examiner conducting the histological analysis was blinded to the clinical and on-screen scores assigned by the examiner performing the visual and intraoral scanner assessments.	• **Intraoral Scanner (TRIOS 4)**<br>**On-screen tooth-colour 3D models**<br>Intra-rater reliability = 0.86<br>**On-Screen Colour + Fluorescence 3D Models**<br>Intra-rater reliability = 0.80	• **ICDAS (Visual)**<br>Examinations were conducted under a dental lamp both before and after air-drying the teeth.<br>• **Intraoral Scanner (TRIOS 4)**<br>During scanning, the dental lamp was turned off to avoid interference, and the procedure followed the manufacturer's recommendations to ensure accurate image acquisition. The scanned teeth were then analyzed using TRIOS software.<br>• **Histological**<br>Teeth were sectioned buccolingually with a precision diamond disk. The sections were examined using a stereomicroscope (Zeiss SteREO Discovery) and specialized software (DeltaPix InSight) with a measurement precision of 0.01 mm.	• The inability of the on-screen method to replicate the wet-dry transitions of clinical examinations might have impacted the sensitivity for detecting early lesions.<br>• Lesions in the outer third of dentin were not separately presented in the results due to the lack of direct correspondence with ICDAS scoring. This may have affected the reliability of discriminating between lesions in enamel and outer dentin.	• The teeth were extracted for therapeutic reasons, such as orthodontic treatment or periodontal issues, which may not fully represent the general population<br>• Although blinding was implemented for histological analysis, the study did not address other potential observer-related biases comprehensively<br>• The study assessed only primary occlusal caries lesions and did not evaluate other types of lesions, such as proximal caries, caries in aesthetic areas, or caries around restorations.	Unlike clinical visual examination, where lighting and angle inconsistencies can affect results, 3D models are unaffected by these factors. This enhances reproducibility and objectivity in diagnostic evaluations.<br><br>The high resolution of the models and their ability to document and monitor caries progression over time make them valuable for clinical research and as a tool for teaching caries detection and management.
Kanar O. et al., 2023	The examiner was blinded to the patient's identities during the scoring process	• Not specified	• **ICDAS (Visual)**<br>Examinations were performed under standardized lighting conditions, using 5500 K illumination.<br>• **Intraoral Scanner (iTero Element 5D)**<br>iTero Element 5D was operated following the manufacturer's guidelines. The 3D images were viewed on the Myitero software.<br>• **Digital radiograph**	• For validation, temporary tooth separation was used in a small number of cases to allow better visualization of interproximal lesions. However, this method may introduce some variability in results due to the limited ability to observe certain lesions.<br>• The intraoral scanner feature is unable to detect root caries, which limits the scope	• The main examiner, though calibrated, had limited prior experience with the iTero Element 5D scanner. The authors suggested that more experienced observers might achieve greater accuracy, particularly with the NIRI feature.<br>• The study relied on clinical and tactile confirmation for validation, with no use of histological examination, which might have provided a more definitive assessment.	A single, calibrated examiner conducted all scoring to ensure consistency and minimize intra-rater variability. While this approach enhances reproducibility within the study, the absence of inter-rater assessments means reproducibility across different observers was not evaluated.<br><br>The study pointed out that the iTero Element 5D might overestimate caries, especially in

			**BWR**<br>The radiographs were taken using Vistascan phosphor plates with exposure settings of 60–70 kV, 4–6 mA, and a 0.16-second exposure time.<br>**PR**<br>Images were obtained using fixed parameters of 64 kV, 7 mA, and a 16-second exposure time.	of the technology in diagnosing all types of carious lesions.<br>• Developmental or acquired defects (such as fluorosis or molar-incisor hypomineralization) could cause lesions to appear brighter in the iTero Element 5D system, which could lead to overestimation of caries in those areas.	• The study was conducted on a specific patient group (22 participants) with specific exclusion criteria, which might limit generalizability to broader populations.	premolars, due to brightness artifacts caused by structural defects or convex anatomical surfaces.
Edrees A. et al., 2023	The authors stated that blinding was not applicable in this study because the examiners were restricted from sharing their study information.	• **ICDAS-II (Visual)**<br>Inter-rater reliability = 0.94 (nearly perfect) and intra-rater reliability = 0.94 (Examiner 1) and 0.87 (Examiner 2) (nearly perfect)<br>• **Intraoral camera (VistaCam iX Proxi HD)**<br>Inter-rater reliability = 0.96(nearly perfect) and intra-rater reliability = 0.88 (Examiner 1) and 0.86 (Examiner 2)<br>• **Digital Radiograph (BWR)**<br>Inter-rater reliability = 0.88 (nearly perfect) and intra-rater reliability = 0.84 (Examiner 1) and 0.88 (Examiner 2) (nearly perfect)	• **ICDAS-II**<br>Visual examinations were conducted under standardized dental unit light conditions. A front-surface dental mirror, an oil-free air syringe for drying teeth, and cotton rolls for isolation were used to enhance visibility.<br>• **Intraoral camera (VistaCam iX Proxi HD)**<br>The device was calibrated and used following the manufacturer's instructions. The head of the intraoral camera was wrapped in a protective cover provided by the manufacturer. The images were analyzed using the DBSWIN software.<br>• **Digital Radiograph (BWR)**<br>A Heliodent DS intraoral X-ray machine with a RINN film holder was used to minimize overlap and ensure standardized imaging angles. Images were scanned using a phosphor plate scanner.	• The study found that the VistaCam iX Proxi HD performed better at detecting dentinal caries compared to enamel lesions. The device struggled with visualizing enamel lesions, potentially due to differences in enamel thickness or surface curvature, which could lead to false positives or missed diagnoses.<br>• The study notes that the quality of images from the VistaCam iX Proxi HD system could impact the accuracy of caries detection, particularly for enamel lesions. While efforts were made to ensure high-quality imaging, variations in image quality could affect diagnostic outcomes.	• Blinding of the observers was not possible due to the nature of the diagnostic methods, which might have introduced some degree of bias despite efforts to restrict information sharing.<br>• The study did not use histological validation as a reference standard, which could have provided a more definitive assessment of caries detection accuracy.<br>• Although the study included 102 teeth from 36 participants, the authors acknowledged that a larger sample size might be necessary to validate the results further.	High levels of agreement were reported, with near-perfect Kappa values for both inter- and intra-observer reliability across all diagnostic methods (ICDAS-II, BWR, and VistaCam). This strong reliability was attributed to the comprehensive calibration sessions and training conducted before the study, during which observers practiced the diagnostic methods and resolved discrepancies until a full agreement was reached.<br><br>The authors highlighted that VistaCam overcame challenges commonly associated with BWR, such as surface overlap, which can hinder the accurate detection of enamel lesions. However, they also acknowledged VistaCam's limitation in reliably distinguishing between healthy and carious dentin, which can affect its diagnostic precision for deeper lesions.
Salma M. et al., 2022	Examiners were unaware of each other's findings	• **ICDAS-II (Visual)**<br>Inter-rater reliability = 0.85 (nearly perfect) and intra-	• **ICDAS-II (Visual)**<br>Conducted under standardized operatory light from a dental unit with no additional lighting specifications given.	• The study notes that stains, especially dark stains, could lead to false positives when using the fluorescence device, as they might interfere with the	• Ethical restrictions prevented the use of fissurotomy for validating lesions classified as ICDAS-II scores 0 and 1, which are typically indicated for remineralization. As a	The study reported high intra- and inter-observer reliability for the ICDAS-II and VistaProof HD Smart methods. This might be due to thorough

		rater reliability = 0.89 to 0.94 (high)<br>• **Intraoral camera (VistaProof HD)** Inter-rater reliability = 0.87(nearly perfect) and intra-rater reliability = 0.81 to 0.84 (high)	Teeth were examined visually while wet, then after being dried for 5 seconds with a triplex syringe.<br>• **Intraoral Camera (VistaProof HD)** In order to maintain a consistent angle, the camera was positioned perpendicular to the occlusal surface of the teeth. Isolation was achieved with cotton rolls and a suction tip, and the tooth surface was dried with a triplex air syringe for 15 seconds before imaging. A spacer was used to maintain a set distance and reduce extraneous light penetration during imaging. Images were analysed in DBSWIN software.<br>• **Histological (Fissurotomy)** A Bar was inserted into the fissures and then examined using 4X magnification loupes. The lighting conditions for this process were not specified beyond the use of standard operatory lighting.	fluorescence readings. Proper fissure cleaning is crucial to avoid these issues.<br>• VistaProof showed low sensitivity in detecting early enamel carious lesions, which could be related to the device's inability to quantify scattered fluorescence light in early enamel lesions.	result, the study's accuracy assessment primarily applied to dentin lesions.	examiner calibration and standardized viewing conditions during the assessments.
Wang F., 2022	The examiner was blinded to the histological assessment results.	• **ICDAS-II (Visual)** Intra-rater reliability = 0.84<br>• **Intraoral camera (DIAGNOcam)** Intra-rater reliability = 0.90 (moderate agreement)<br>• **Digital Radiography (BWR)** Intra-rater reliability = 0.91	• **ICDAS-II (Visual)** The examiner viewed the teeth wet first and then dried them with air, and then explored with a WHO probe.<br>• **Intraoral Camera (DIAGNOcam)** The device was used following the manufacturer's calibration guidelines to optimize image quality<br>• **Radiograph** Planmeca X-ray unit and Kwik-Bite Senso aiming device were used with parameters such as 60 kV, 8 mA, 0.125 s<br>• **Histological** After this, the teeth were extracted and sectioned using a diamond bar. The	• The study notes that mild developmental enamel defects, such as enamel hypoplasia or dental fluorosis, may interfere with the caries detection methods, especially when using near-infrared light transillumination (NIRLT). These defects may cause shadows that resemble carious lesions, leading to false positives.<br>• As the study was conducted on premolars from orthodontic patients, the natural contact between teeth may have been disrupted, which could impact the detection of proximal caries, as carious	• Although the study was conducted in vivo, it did not account for potential patient-related factors such as movement, saliva, or soft tissue interference, which could affect image quality and diagnostic accuracy in a real clinical setting.<br>• Since one examiner conducted all methods, their specific training and experience could influence the consistency and accuracy of the results, particularly for methods requiring visual assessment.<br>• The study did not include a follow-up, meaning it could not assess the progression or stability of detected lesions over time,	The study evaluated intra-examiner reliability by having the single examiner reassess a set of 20 teeth after a one-week interval. Weighted kappa values were high for all methods, indicating strong reproducibility for the examiner across DIAGNOcam, ICDAS-II, and BWR.<br><br>The authors suggested that future studies involve multiple examiners to further assess inter-rater reliability and validate the findings.

			sections were viewed with 17.0X magnification.	lesions in tightly contacted teeth may be harder to detect visually.	which would be relevant for evaluating diagnostic performance in preventive care.	
Valizadeh S. et al., 2022	Examiners were blinded to the histological assessment results.	• **Intraoral camera (VistaCam IX Proxi)**<br>Inter-rater reliability = 0.82 (Observer 1) and 0.56 (Observer 2), Intra-rater reliability = 0.85 (Observer 1) and 0.68 (Observer 2)<br>• **Digital Radiography (BWR)**<br>Inter-rater reliability = 0.76 (Observer 1) and 0.72 (Observer 2), Intra-rater reliability = 0.79 (Observer 1) and 0.69 (Observer 2)	• **Intraoral Camera (VistaCam IX Proxi)**<br>Teeth were dried before imaging, and the camera was positioned according to the manufacturer's instructions using a special holder. Images were captured in a dimly lit room to simulate the oral environment. DBSWIN software was used to interpret the results.<br>• **Digital Radiograph (BWR)**<br>Radiographs were taken under standardized conditions using a Gendex intraoral radiography unit and ACTEON photostimulable phosphor plates. SCANORA software was used to interpret the results<br>• **Histological**<br>Teeth were sectioned using a Buehler Isomet saw and observed under a stereomicroscope (Olympus SZX9)	• The accuracy of the diagnostic tools (VistaCam IX Proxi and BWR) can be influenced by image quality, positioning of the camera, and subjective interpretation by examiners. These factors may introduce variability in the results.	• The study was conducted under laboratory conditions, which may not fully replicate the clinical environment<br>• Small samples included, which may limit the generalizability of the findings.<br>• The exclusion of cavitated lesions limits the applicability of the results to early-stage caries.	The inter- and intra-observer reliability values indicate that both diagnostic methods are reproducible, but VistaCam IX Proxi showed greater variability in inter-observer agreement compared to BWR. This suggests that VistaCam IX Proxi's reproducibility could be optimized further with enhanced training and standardized protocols, especially since its sensitivity makes it valuable for early caries detection.
Mokhtar IW, et al., 2021	Examiners were blinded to each other's radiographic and intraoral camera diagnoses	• **Intraoral camera (DIAGNOcam)**<br>Inter-rater reliability = 0.51, Intra-rater reliability = 0.68 (Examiner 1) and 0.62 (Examiner 2)<br>• **Digital Radiography (BWR)**<br>Inter-rater reliability = 0.63, Intra-rater reliability = 0.87 (Examiner 1) and 0.76 (Examiner 2)	• **ICDAS (Visual)**<br>Teeth were cleaned and air-dried, and the evaluation was conducted under standardized dental chair lighting<br>• **Intraoral Camera (DIAGNOcam)**<br>The device was calibrated according to the manufacturer's instructions. However, specific lighting conditions during image taking were not mentioned.<br>• **Digital Radiograph**<br>Radiographs were taken using tube voltage 60-65 kVp, tube current 7mA, and exposure time 0.08 seconds. Images were evaluated in EasyDent V4 software.	• The study notes that the occlusal surface fissure system in children can make teeth more susceptible to caries. Deep fissures might complicate the detection of caries, leading to misdiagnoses.<br>• The study mentions that factors like plaque and calculus were considered and eliminated through a strict cleaning procedure to ensure the accuracy of the diagnostic methods.	• The study included only 52 occlusal surfaces, which might limit the generalizability of the results.<br>• The study population was restricted to children aged 7–15 years, and findings may not be applicable to other age groups.<br>• The absence of histological analysis as the reference standard could affect the accuracy of the diagnostic performance metrics.<br>• Authors mentioned the low prevalence of dentine caries as a factor that might influence diagnostic performance metrics.	The reproducibility of the DIAGNOcam device was particularly promising, despite differences in the examiners' experience levels.<br><br>The authors also emphasized that practitioners develop individual diagnostic approaches based on experience, influencing caries detection and treatment choices. Therefore, a learning curve in using the DIAGNOcam should be considered when implementing it in clinical practice.

Michou S. et al., 2021	Examiners were blinded to the results of other diagnostic methods.	• **ICDAS-II (Visual)** Intra-rater reliability = 0.88<br>• **Intraoral scanner (TRIOS 4)** Intra-rater reliability = 0.96<br>• **Intraoral Camera (DIAGNOcam)** Intra-rater reliability = 0.87<br>• **Digital Radiography** Intra-rater reliability = 0.91	• **ICDAS-II (Visual)** Controlled lighting conditions were maintained, but specific details about the type or intensity of light were not provided.<br>• **Intraoral Scanner (TRIOS 4) and Intraoral Camera (DIAGNOcam)** Both methods were operated following the manufacturers' guidelines. The setup ensured no external light interference, as the devices were equipped with built-in illumination systems.<br>• **Digital Radiograph** Radiographs were taken in a buccolingual direction using standardized exposure parameters such as tube voltage of 60 kVp, tube current of 7mA, and exposure time of 0.25 seconds.<br>• **Histological** Teeth were sectioned buccolingually using a cutting machine (Accutom, Struers A/S) equipped with a diamond blade (~0.4 mm thickness). The sections were examined under a stereomicroscope (Zeiss SteREO Discovery) at magnifications up to ×0.79.	• The quality of NIR images depends on the angle of the light source, which can affect the detection of caries. Incorrect angles could lead to false positives or false negatives in detecting lesions, especially for initial enamel lesions.<br>• The presence of anatomical features such as developmental defects or other mineralization issues can interfere with the clarity of the NIR images, potentially leading to diagnostic errors. For instance, areas of demineralization or developmental defects might be misinterpreted as caries.<br>• The study noted that adjustments to the image settings, such as brightness and contrast, are subjective and depend on the examiner's judgment, which could impact the consistency and reliability of the results.	• The study focused exclusively on proximal surfaces, which limits the generalizability of the findings to other tooth surfaces or types of lesions.<br>• The teeth and surfaces analyzed were preselected based on the presence of carious lesions, which may not accurately represent the natural distribution of lesions typically encountered in clinical practice.<br>• There was an overrepresentation of proximal lesions spanning varying severities (E1–D3), potentially affecting diagnostic accuracy compared to a more uniformly distributed sample.	The study highlighted that the TRIOS 4 prototype demonstrated the highest reproducibility among the diagnostic methods, suggesting its potential for consistent performance in clinical settings.<br><br>The high intra-rater reliability observed across all methods was attributed to the extensive training and expertise of the examiners. However, the authors acknowledged that this level of consistency might not be achievable in general clinical practice, particularly by less experienced clinicians.<br><br>Additionally, the authors emphasized that the ICDAS system, employed for visual and radiographic assessments, requires thorough training to ensure accurate and reproducible scoring. This underscores the critical role of examiner experience in achieving reliable diagnostic outcomes.
Alrayyes S. et al., 2021	The raters were blinded to the pairing of images from DEXIS CariVu and BWR corresponding to the same interproximal surfaces.<br><br>Additionally, the examiners were blinded to the diagnostic results	• **Intraoral Camera (CariVu)** Inter-rater reliability = 0.48, Intra-rater reliability = not specified<br>• **Digital Radiography (BWR)** Inter-rater reliability = 0.78, Intra-rater reliability = not specified	• **Clinical validation for reference standard** This involved cleaning the interproximal surfaces, visually inspecting them under clinical lighting, and probing when necessary.	• The study acknowledges that raters may have been less experienced with the CariVu device, which could have affected their ability to accurately score caries, leading to lower interrater reliability.	• The study included a relatively small number of participants and evaluated only 90 interproximal surfaces, which might limit the generalizability of the findings.<br>• The study relied on clinical visual-tactile examination as the reference standard, without using histological analysis to confirm findings.<br>• The study was conducted exclusively on pediatric patients in the mixed dentition	The authors emphasized that the lower reliability for CariVu was likely due to the learning curve associated with interpreting its images, suggesting that improved training could enhance reproducibility.<br><br>Raters highlighted the ease of using CariVu for pediatric patients but noted the need for better training to address its interpretative challenges.

	from the CariVu and radiographic assessments.	• The study included a questionnaire to gather feedback from the raters regarding their experiences with the diagnostic methods			phase, which may not represent other age groups or dentition stages.<br>• Only images that met specific criteria for quality were included in the study, which may exclude cases with suboptimal images, potentially affecting the generalizability of the results.	The study stressed the complementary roles of CariVu and radiography, with CariVu excelling in patient compliance and radiation safety, while radiography remains the gold standard for caries detection.
Michou S. et al., 2021	Examiners were blinded to the results of other diagnostic methods.	• **ICDAS (Visual)** Intra-rater reliability = 0.85<br>• **Intraoral scanner (TRIOS 4)** Intra-rater reliability = > 0.80 across all algorithms<br>• **Histology** Intra-rater reliability = 0.90	• **ICDAS (Visual)** Standardized lighting conditions were maintained<br>• **Intraoral Scanner (TRIOS 4)** The method was operated according to the manufacturer's guidelines, with the setup ensuring the dental lamp was switched off and the external light was minimized.<br>• **Histological** Extracted teeth were sectioned buccolingually using a precision cutting machine equipped with a diamond blade (~0.4 mm thickness). The sections were examined under a stereomicroscope (Zeiss SteREO Discovery)	• In some cases, insufficient cleaning of the occlusal surfaces, particularly in third molars, could have influenced the fluorescence signal. The presence of dental biofilm, which emits strong red-orange fluorescence, could lead to false indications by the system.<br>• Limited access to the third molars during in vivo scanning and insufficient scanning data in some areas of the occlusal surface led to algorithm failures, indicating that the scanner's performance may be influenced by physical accessibility and scan quality.<br>• Signal variations between in vivo and in vitro scans were observed, likely due to differences in environmental factors such as light exposure and storage conditions post-tooth extraction. This led to discrepancies in sensitivity for caries detection.	• The teeth included were preselected based on the presence of carious lesions, potentially leading to an overrepresentation of specific lesion severities and underrepresentation of others.<br>• The study focused solely on occlusal surfaces, limiting the generalizability of the results to other tooth surfaces or lesion types .<br>• The findings were based on an in vitro validation and may not fully translate to in vivo clinical settings. The authors emphasized the need for further in vivo studies to confirm the clinical applicability of the automated algorithms.	TRIOS 4, equipped with automated algorithms, provides a non-invasive and objective diagnostic tool that minimizes operator variability and delivers consistent results.<br><br>The authors emphasized the necessity of conducting in vivo studies to validate the reproducibility and establish diagnostic thresholds for the automated caries detection algorithms in real clinical conditions.<br><br>They also suggested that combining visual examination with automated algorithms could significantly enhance diagnostic accuracy, especially in busy clinical settings where consistency and efficiency are crucial.
Metzger Z. et al., 2021	The expert team interpreting the 3D images and radiographs was blinded to the clinical outcomes and other diagnostic results	• **Intraoral scanner iTero Element 5D) and digital radiography (BWR)** **Early enamel lesions** Interrater reliability between intraoral scanner (iTero Element 5D) and radiograph among dentists was 0.24	• **Intraoral Scanner (iTero Element 5D)** The iTero Element 5D scanner was operated following the manufacturer's guidelines<br>• **Digital Radiographs (BWR)** Radiographs were taken based on the settings that are routinely used in their respective clinics.	• The study involved multiple dental clinics, each using their standard radiographic equipment, which could introduce variability in the BWR used for comparison. This lack of standardization in radiographic methods may have impacted the results and the comparison between the two diagnostic methods.	• The study did not use histological sections or micro-CT as a definitive reference standard due to the clinical nature of the study, relying instead on radiographs as the comparator despite its known limitations.<br>• Although 59 lesions were observed during caries excavation, the sample size for this validation was relatively small, and not all	iTero Element 5D showed higher reproducibility among expert examiners compared to dentists, highlighting the importance of training for consistent performance.<br><br>The iTero Element 5D system was noted for its ability to provide reliable caries detection without the use of ionizing radiation, making it a

		(fair agreement), and among the expert team was 0.51 (moderate agreement)<br>**Dentino-enamel junction (DEJ)**<br>Interrater reliability between intraoral scanner (iTero Element 5D) and radiograph among dentists was 0.50 (moderate agreement), and among the expert team was 0.86(high agreement)	• **Clinical validation for reference standard**<br>Dentists directly observed the extent of the carious lesions and documented whether they were confined to enamel or had extended into dentin with an excavator. The findings during the excavation were compared to the other diagnostic outcomes.		lesions could be evaluated during excavation, especially those diagnosed as intact.<br>• The expert team, which evaluated the 3D images, was trained by the study sponsor, which may have introduced potential bias in interpreting the results. However, to mitigate this, the data was presented as isolated individual images to reduce bias.	safer alternative, particularly for children and other vulnerable populations.
Stratigaki E. et al., 2020	Examiners were unaware of each other's findings.	• **Intraoral camera (DIAGNOcam)**<br>**Initial Round**<br>Inter-rater reliability = 0.42 and intra-rater reliability = 0.6<br>**Recall Round**<br>Inter-rater reliability = 0.51 and intra-rater reliability = 0.62<br>• **Digital Radiograph (BWR)**<br>**Initial Round**<br>Inter-rater reliability = 0.60 and intra-rater reliability = 0.87<br>**Recall Round**<br>Inter-rater reliability = 0.63 and intra-rater reliability = 0.76	• **Intraoral Camera (DIAGNOcam)**<br>Images were analysed in KID software. Other device-related calibrations are not provided.<br>• **Digital Radiograph (BWR)**<br>For the Heliodent device, the exposure time was 0.06 s at a cathode voltage of 60 kV and 7 mA of amperage, and for HDX (Dental EZ), the exposure time was 0.09 s at 65 kV and 7.5 mA cathode voltage and amperage. X-rays were assessed using a negatoscope and film magnifier.<br>• **Composite Reference standard**<br>An operating microscope at 6.4x Magnification was used to locate the caries lesions on two groups of patients (A = non-operative, B operative).<br>• **Image Viewing**<br>All x-rays were analysed in a darkened room with the option of adjusting the brightness and contrast in the software.	• The study acknowledges that there was variability in the diagnostic performance of both BWR and DIAGNOcam, especially with the DIAGNOcam showing lower specificity in some cases. This might be influenced by factors such as the positioning of the device and the presence of anatomical variations in teeth that could affect the diagnostic readings.<br>• The study was conducted across two different centers, and while digital radiographs were used, there were variations in the radiographic methods employed in each center. While this may not be a major confounder, it could have introduced some variability in the results.	• Since histological analysis could not be performed on healthy teeth or non-cavitated lesions for ethical reasons, a composite reference standard was used (involving orthodontic separation rubber and bi-optical validation) to assess early lesions. While this approach was necessary, it may introduce some bias or limitations in the accuracy of caries detection.	Intra-examiner reliability improved from the initial assessments to the recall while using DIAGNOcam, suggesting a potential need for training to achieve comparable reproducibility in clinical settings.
Michou S. et al., 2020	The main investigator conducted the intraoral	• **ICDAS (Visual)**	• **ICDAS (Visual)**	• The study includes a higher proportion of initial caries lesions, with fewer	• Difficulty in establishing distinct cutoffs for more advanced lesions (D2 and D3) due to	The authors reported strong reliability for all diagnostic methods, indicating that the methods

	scanner and histological assessments without knowledge of the results from the visual and radiographic assessments.<br><br>The visual and radiographic assessments were performed by two independent examiners, who were blinded to the results of the other index tests and the reference test.	Intra-rater reliability = 0.87 (strong reliability)<br>• **Intraoral scanner (TRIOS 3)**<br>Intra-rater reliability = 0.96 (almost perfect reliability)<br>• **Digital Radiography**<br>Intra-rater reliability = 0.91 (strong reliability)	During the visual examination, controlled lighting was used<br>• **Intraoral Scanner (TRIOS 3)**<br>The intraoral scanner was operated according to the manufacturer's guidelines, and teeth were kept humid during the scanning to prevent dehydration. Teeth were scanned inside a dark box to eliminate external light influence and simulate intraoral conditions in the posterior region.<br>• **Digital Radiograph**<br>Radiographs were taken in a buccolingual direction using standardized exposure parameters such as tube voltage of 70 kVp and exposure time of 0.25 seconds.<br>• **Histological**<br>Teeth were sectioned buccolingually along the long axis using a cutting machine (Accutom, Struers A/S) equipped with a diamond disk (~0.4 mm thickness). The sections were photographed under optical magnification (×0.79) using a stereomicroscope (Zeiss SteREO Discovery)	moderate and extensive lesions. This bias could affect the accuracy of performance measures for more severe caries stages, as initial lesions were more prevalent in the sample<br>• Differences in light conditions, bacterial biofilm presence, and humidity between the in vitro setup and the clinical environment could impact the intraoral scanner's signal and interpretation.<br>• TRIOS 3 used different mathematical functions to calculate caries scores, and the performance varied depending on the function used. For example, function f1 (based on the ratio of red to green fluorescence) showed poorer performance compared to other functions (f2, f3, and f4), potentially due to technological differences in the scanner prototype.	overlapping fluorescence signal intensities. However, the authors noted that such cutoffs might not add significant clinical value, as these stages are often easily identified visually.<br>• Only one examiner per index test was used, which could introduce bias. The examiners performing visual-tactile and radiographic assessments were highly experienced, potentially leading to results that are not generalizable to less experienced clinicians.<br>• The findings are based on an in vitro setup, and the authors emphasized the need for in vivo validation to establish clinical relevance and define definitive cutoffs.	(i.e., TRIOS 3, BWR, and visual) are consistently reproducible when performed by the same observer.<br>The fluorescence-based caries scoring system for TRIOS 3 showed potential for more objective caries detection compared to visual and radiographic methods. However, validating clinical cutoff points in vivo is essential to enhance their practical application.<br><br>The authors highlighted the potential for using scanners for remote diagnostics, enabling long-term monitoring and caries detection in underserved areas.
Alamoudi et al., 2019	Examiners were blinded during evaluations. Those assessing DIAGNOcam images were unaware of radiographic scores, and those conducting direct visual examinations were blinded to both	• **ICDAS-II (Visual)**<br>Inter-rater reliability = 0. 86, Intra-rater reliability = 0.86 to 0.87<br>• **Intraoral camera (DIAGNOcam)**<br>Inter-rater reliability = 0.73 to 0.86, Intra-rater reliability = 0.74 to 0.86	• **ICDAS-II (Visual)**<br>The examination was performed under a dental chair light to ensure adequate illumination.<br>• **Intraoral Camera (DIAGNOcam)**<br>The device was calibrated according to the manufacturer's instructions, and images were taken with the dental unit's light turned off to avoid interference.	• This method of separating teeth to assess cavitation status may cause some discomfort for the child and may also occasionally lead to gingival inflammation. This could potentially affect the accuracy or reproducibility of clinical assessments.	• The study acknowledges that direct visual examination after tooth separation was used as a validation method, but this is not considered the "gold standard" like histological validation, which could potentially limit the accuracy and reliability of the findings	The study demonstrated a high level of agreement among the examiners. This reflects the reliability of the examiners and the diagnostic process.<br><br>The DIAGNOcam showed slightly lower inter-rater reliability compared to radiographs and visual examination. This might indicate differences in interpretation or the need for more

	radiographic and DIAGNOcam scores.	• **Digital Radiography (BWR)**<br>Inter-rater reliability = 0.84 to 0.85, Intra-rater reliability = 0.85	• **Digital Radiograph**<br>The Orix 70 X-ray unit was used with parameters such as 70 kV, 7 mA, exposure time of 0.05 seconds. The films were processed with a VistaScan Mini image plate scanner to ensure consistent quality, and the images were subsequently evaluated in a dark room on a 19-inch screen.		• The need for two appointments for temporary tooth separation may introduce practical challenges.<br>• While sufficient for statistical analysis, a larger sample size could improve the generalizability of the findings	standardized training for novel technologies such as the DIAGNOcam.<br><br>Visual examination maintained the highest reliability values, indicating its consistent performance when applied with standardized protocols.
Tonkaboni A. et al., 2018	Examiners assessed the teeth samples without knowledge of the histological results.<br><br>However, no information is provided on whether they were blinded to each other's assessments.	• **ICDAS-II (Visual)**<br>Intra-rater reliability = 0.55 (moderate agreement)<br>• **Intraoral camera (VistaCam iX)**<br>Did not report specific inter- or intra-rater reliability values for VistaCam iX<br>• **Digital Radiography (BWR)**<br>Intra-rater reliability = 0.72 (substantial agreement)	• **ICDAS-II (Visual)**<br>The examiners used a dental mirror, explorer, and air spray to detect the caries under standardized operatory lighting.<br>• **Intraoral Camera (VistaCam iX)**<br>Calibrated based on the manufacturer's instructions. A light system was used, however, the details are unclear.<br>• **Digital Radiograph (BWR)**<br>A standardized setting (60–70 kV and 7 mA) and distance (0.5cm from film to tooth and 32 cm from film to tube) were used. Afterward, the images were processed in a film processor (Velopex, Extra-x).<br>• **Histological**<br>Tooth was sectioned mesiodistally (1-mm thick slices). These sections were then examined under a stereomicroscope at 10x magnification.	• The presence of enamel cracks in some teeth led to false-positive results with VistaCam iX. These cracks could result in bright areas appearing during infrared imaging, even in the absence of caries.<br>• The quality of the images captured by VistaCam iX can be affected by the positioning of the device and the light reflecting off the enamel, which might influence the accuracy of caries detection.	• The controlled laboratory environment may not fully replicate the complexities of an in vivo clinical setting<br>• Lack of factors like saliva, plaque, and other oral biofilms was noted as a limitation<br>• Only two examiners participated in the study, which may not reflect the broader variability that could occur with a larger sample of clinicians in actual practice	The authors noted that the infrared imaging's sensitivity of VistaCam iX could support preventive approaches by identifying lesions earlier, potentially reducing the need for more invasive treatments.
Iranzo-Cort es, 2018	Examiners were unaware of each other's findings, as well as to the histological findings	• **ICDAS-II (Visual)**<br>Inter-rater reliability = 0.76 (good agreement) and intra-rater reliability = 0.82 and 0.91 for the two examiners (high)<br>• **Intraoral camera**	• **ICDAS-II (Visual)**<br>The examiners viewed the teeth wet first and then dried with air for 5 seconds to observe any color change<br>• **Intraoral Camera (VistaProof)**<br>Before capturing images with the VistaProof device, the teeth were dried to	• Authors noted that external illumination might influence the capture and processing of fluorescence images.<br>• Additionally, they acknowledged that enamel cracks and other structural variations might result in false-positive readings with VistaProof, as these	• The study was conducted in vitro, meaning the findings might not fully translate to clinical (in vivo) settings due to the controlled environment<br>• The examiners had limited training with the VistaProof device, which could affect diagnostic accuracy.	The authors highlighted that ICDAS-II consistently showed strong diagnostic reliability and higher reproducibility than other methods, which makes it practical for routine clinical use.<br><br>The authors noted that more extensive training with VistaProof might improve its diagnostic

		**(VistaProof)**<br>Inter-rater reliability = 0.65 (moderate agreement) and intra-rater reliability = 0.76 and 0.78 for both examiners (high)	avoid misleading images due to moisture. The authors followed the manufacturer's instructions, but they did not specify additional calibration steps. Images were later processed in the DBSWIN program software.<br>• **Histological**<br>The teeth were longitudinally sectioned and polished with Sof-Lex discs. The sections were then examined under a microscope at 2.5x and 30x magnification.	imperfections could appear similar to caries under fluorescence.	• The study focused on extracted teeth, primarily with incipient lesions, which may not represent the full range of caries presentations seen in a clinical setting.	accuracy and reproducibility. They suggested that VistaProof, when used in combination with ICDAS-II and with adequate training, could support clinicians in detecting early carious lesions more effectively.
Elhennawy K. et al., 2018	Examiners were blinded to the results of other diagnostic methods	• **Visual**<br>Inter-rater reliability = 0.78 good agreement) and intra-rater reliability = 0.72 (good agreement)<br>• **Intraoral camera (DIAGNOcam)**<br>Inter-rater reliability = 0.74(good agreement) and intra-rater reliability = 0.64 (good agreement)<br>• **Digital Radiograph (BWR)**<br>Inter-rater reliability = 0.76 (good agreement) and intra-rater reliability = 0.81 to 0.92 (excellent agreement)	• **Visual**<br>A standardized dental light (Heliodent Plus by Sirona Dental Systems) was used to illuminate the teeth during assessments<br>• **Intraoral camera (DIAGNOcam)**<br>The device was calibrated based on the manufacturer's instructions. During the procedure, the dental unit light was switched off during examinations to avoid light interference<br>• **Digital Radiograph (BWR)**<br>The Heliodent Plus intraoral X-ray unit, equipped with a digital sensor, was operated at 65 kV, 7 mA, with an exposure time of 0.06 seconds. Radiographs were then evaluated on a 27-inch diagnostic-grade screen under standardized lighting conditions.<br>• **Reference Standard (Transverse Microradiography)**<br>Teeth were hemisectioned in thin sections (100 µm) using a band saw and polished with abrasive paper. Microradiographs were captured with a	• The study mentions the challenge of accurately identifying lesions beneath composite restorations. Some subsurface lesions might be missed or misinterpreted due to the restorative material's properties, such as its optical characteristics (e.g., light reflection or scattering), which can affect diagnostic outcomes.<br>• The authors mention that the DIAGNOcam was used in a non-standardized way (live imaging in motion), which might impact its performance compared to when the device is used in a standardized manner.	• The study was conducted under controlled in vitro conditions, which might not fully replicate the complexities of a clinical setting, such as patient-related factors (e.g., saliva, movement, or soft tissue interference)<br>• Teeth with existing occluso-proximal composite restorations were specifically included, which may limit the generalizability of findings to other types of restorations or natural tooth structures.<br>• Although the setup simulated clinical conditions (e.g., soft tissue scattering), it might still lack the dynamic challenges encountered during clinical diagnostics, such as visual obstructions and lighting variability.	The authors emphasized that substantial to almost perfect Kappa values for both inter- and intra-rater reliability across all diagnostic methods confirmed the reproducibility of the assessments by trained examiners.<br><br>However, the authors acknowledged that while the controlled in vitro setup enhanced reproducibility, it does not fully replicate real-world conditions where factors such as patient anatomy, saliva, and movement could impact consistency. This underscores the need for clinical studies to validate these findings in practical settings.

			nickel-filtered copper X-ray source and analyzed with digital image analysis software. Mineral loss greater than 400 vol.% was used as the threshold for defining carious lesions.			
Baltacioglu I.H. et al., 2017	Examiners were blinded to the findings of other diagnostic methods and the reference standard.	• **Intraoral camera (DIAGNOcam)**<br>Inter-rater reliability = 0.78 (first reading), 0.75 (second reading),<br>Intra-rater reliability = 0.79 (Examiner 1) and 0.88 (Examiner 2)<br>• **Digital Radiography (BWR)**<br>Inter-rater reliability = 0.61 (first reading), 0.74 (second reading),<br>Intra-rater reliability = 0.62 (Examiner 1) and 0.67 (Examiner 2)	• **Intraoral Camera (DIAGNOcam)**<br>The device was calibrated according to the manufacturer's instructions. However, specific lighting conditions during image taking were not mentioned.<br>• **Digital Radiograph**<br>Radiographs were taken using standardized exposure parameters (specific settings such as kVp and exposure time were not explicitly mentioned). PSP plates were scanned and processed using the Digora Optime system<br>• **Clinical validation for reference standard**<br>Lesions were visually inspected and opened using a round bar to confirm their presence and extent.	• The study did not specify any confounding factors	• The study included a limited number of teeth, which might reduce the generalizability of the results<br>• The study mentions that it was not possible to conduct a completely unbiased investigation of intraobserver and interobserver reliability due to the use of different types of radiographic and intraoral images.<br>• Histological examination was not used as the reference standard, potentially affecting the accuracy of the diagnostic evaluations.<br>• The study was conducted on a relatively homogenous population, which may not fully represent broader demographic variability.	The authors highlighted that DIAGNOcam demonstrated promising reproducibility, even as a newer diagnostic method, and suggested that experience and targeted training could improve consistency.<br><br>The authors also acknowledged a learning curve in interpreting images while using DIAGNOcam and stressed the importance of proper training to enhance diagnostic accuracy and reproducibility.
Markowitz K. et al., 2015	Not specified	• Not specified	• The study only mentioned that photographs of the sectioned surfaces were taken at eight-fold magnification for analysis. However, does not elaborate on the lighting setup, screen calibration, or environment in which the images were reviewed by examiners.	• Pigmentation or non-carious stains could potentially lead to false positive readings with the Spectra™ Caries Detection Aid, which might impact its diagnostic accuracy.<br>• The study mentions that caries detection might fail when early lesions are deep within the pits and fissures of the tooth, which can obstruct the ability of the caries detector to correctly identify caries, especially in enamel demineralization or subsurface lesions.	• As an in vitro study, these results may not fully reflect real clinical conditions.	Authors confirmed the reproducibility of the device through a calibration exercise, where examiners consistently obtained readings within ±0.2 of each other across multiple assessments. This suggests that the Spectra Caries Detection Aid provides reliable measurements when used by calibrated examiners.

Ko HY. et al., 2015	Not specified	• **ICDAS-II (Visual)**<br>Intra-rater reliability = 0.96<br>• **Intraoral camera (QLF-D)**<br>Inter-rater reliability = 0.78<br>• **Digital Radiography (BWR)**<br>Intra-rater reliability = 0.88<br>• Since only one examiner was involved, inter-rater reliability was not assessed	• **ICDAS-II (Visual)**<br>The observer used an air syringe and a WHO probe to examine approximal surfaces, under standard lighting conditions suitable for visual diagnosis<br>• **Intraoral Camera (QLF-D)**<br>Images were captured in a darkroom with a shutter speed of 1/20s, an aperture value of 13.0, and an ISO speed of 1600. Distance and angle between the QLF-D device and the tooth surfaces were standardized at 10 cm and a 90° angle. Images were analyzed in QLF-D software.<br>• **Digital Radiograph (BWR)**<br>A standardized setting (60 kV and 7 mA with an exposure time of 0.096 seconds) and distance (3cm from the samples and 5 cm from the sensor) were used. Afterward, the images were viewed on a computer screen using the PiViewSTAR software.<br>• **Histological**<br>The teeth were sectioned buccolingual into 2 mm thick slices using a microtome, then ground down to 200 µm for detailed examination under a polarized light microscope (magnifications of 40x and 100x)	• The location of approximal caries, which can be difficult to visualize and access, poses a challenge to detection methods. This anatomical limitation may lead to underestimating the number of carious lesions, especially when using visual methods alone.	• The controlled, laboratory setting may not fully reflect in vivo conditions<br>• Only one observer conducted the assessment, so variability among different examiners could impact results.<br>• While histology provides a gold standard for in vitro validation, its applicability to in vivo diagnostics is inherently limited.	The study demonstrated high reproducibility across methods, which indicates consistent diagnostic performance by the single examiner.<br><br>The authors also noted that the QLF-D's ability to provide quantitative data on lesion extent could reduce subjective variability compared to visual methods and offer a practical, quick tool for tracking caries progression or regression over time.
Jablonski-Momeni A. el al., 2013	Not specified	• **Intraoral Camera (VistaProof)**<br>The intra-class correlation coefficient was 0.89	• **ICDAS (Visual)**<br>Teeth were cleaned, air-dried with a triplex syringe, and examined under standard dental chair light.<br>• **Intraoral Camera (VistaProof)**<br>Manufacturer-provided instructions were followed to take the images	• The study observed that the VistaProof camera was less sensitive in detecting dentine lesions, especially in cases of hidden caries under seemingly intact enamel. This led to false-negative results, which were identified only after fissure opening or radiographic evaluation.	• The study did not use histological validation for all lesions, which could limit the accuracy of the reference standard.<br>• The low prevalence of dentine caries in the study may limit the generalizability of findings to populations with higher caries prevalence.	The authors reported a high level of consistency in repeated assessments using the VistaProof camera, as indicated by its strong intra-rater reliability.<br><br>The study was conducted in a low-caries population, which could influence the diagnostic

			• **Digital Radiograph**<br>No specific details are provided<br>• **Histological (Fissure Opening)**<br>Lesions were clinically opened using rotating instruments.			performance, particularly the high negative predictive value (NPV).
Boye et al., 2012	Examiners assessed both visual and photographic images without knowledge of the histological assessment results	• **BASCD (Visual)**<br>Inter-rater reliability = 0.66 (high) and intra-rater reliability = 0.67 to 0.92 (Median of 0.85)<br>• **Intraoral camera (Sopro 717)**<br>Inter-rater reliability = 0.60 and intra-rater reliability = 0.59 to 0.92 (Median of 0.74)	• **BASCD (Visual)**<br>A Daray X100 lamp was used for visual examinations.<br>• **Intraoral Camera (Sopro 717)**<br>An inbuilt LED light source was used for intra-oral camera photography. The photographs were presented in Microsoft PowerPoint for assessment.<br>• **Histological**<br>A model grinder was used to expose thin layers of the tooth. Each exposed surface was polished, dried, and photographed at approximately 0.16 mm intervals between sections with 10X magnification.<br>• **Image Viewing**<br>Images were assessed in two different ways:<br>Standardized viewing = room with ambient daylight for the first session, and non-standardized viewing = under varying conditions (at any chosen time of the day and room conditions by the examiners) in subsequent sessions	• The study did not specify any confounding factors	• The limited washout period between the first and second caries assessments (14 days apart) could have influenced examiner recall and consistency.<br>• As the study is in vitro, it may not fully represent the in vivo conditions.<br>• The study restricted the caries detection to only the occlusal surfaces of the teeth, which may limit the generalizability of the results, as most carious lesions occur on other surfaces.	Good agreement between standardized and non-standardized viewing suggested minimal impact of physical image presentation conditions on diagnostic outcomes while using Sopro 717 as a diagnostic tool.
Jablonski-Momeni et al., 2012	Not specified	• **Intraoral Camera (VistaCam iX and VistaProof)**<br>Intra-rater reliability = 0.88 to 0.97 for the VistaCam iX	• **Intraoral Camera (VistaCam iX and VistaProof)**<br>Images were captured in a dark box to reduce lighting variations. Images were analyzed by a software named the	• Some investigation sites could not be assessed by the VistaCam iX (FC1) due to technical issues, which could have affected the overall assessment for certain teeth.	• The study generalized the findings due to the controlled experimental setup<br>• Unbalanced sample distribution of different caries stages<br>• As the study is in vitro, it may not fully represent the in vivo conditions.	The authors emphasized that reproducibility and consistency between experienced and less-experienced examiners demonstrate VistaProof's usability, even for those with limited training.

		and from 0.82 to 0.98 for the VistaProof.	DBSWN program. No device calibration is recorded.<br>• **Histological**<br>Crowns were hemi-sectioned using 200 µm-thick grade D64 diamonds and photographed digitally with Leica Zoomsystem Z6 APO M 420/QWin Standard. Images were viewed on a color monitor (FlexScan L 768, EIZO) at a constant distance of 60cm.	• Teeth were stored in a thymol solution after extraction, which may have caused wash-out of some bacterial fluorophores, potentially influencing fluorescence readings.		
Jablonski-Momeni A. el al., 2011	Each observer assessed multiple investigation sites on each tooth and was blinded to the results of the other sites on the same tooth	• **Intraoral camera (VistaProof)**<br>Inter-rater reliability = 0.76 to 0.81 (high) and intra-rater reliability = 0.95 (high)	• **Intraoral Camera (VistaProof)**<br>The device was equipped with 6 blue GaN light-emitting diodes (LEDs) at 405 nm wavelength. Caries lesions were identified in the DBSWIN software.<br>• **Histological**<br>Tooth crowns were sectioned and photographed digitally (Leica Zoomsystem Z6 APO M 420). Images were viewed on a color monitor (FlexScan L 768, EIZO) at a consistent distance of 60cm.	• Stains on occlusal surfaces, fluorosis, and developmental defects are pointed out as possible sources of false-positive readings. These conditions could mimic caries, causing the VistaProof to register sound sites as carious.	• The study was conducted in vitro, meaning the results may not fully translate to clinical settings<br>• Authors mentioned that the clinical database for suggested cutoff values for caries detection with VistaProof is still limited, which calls for caution in interpreting the results. More extensive clinical validation is needed to confirm the efficacy of these cutoff values.<br>• The findings cannot be applied unrestrictedly to clinical practice without further studies.	They highlighted that the VistaProof demonstrated high reproducibility for detecting occlusal caries, suggesting it could be user-friendly and suitable for varying experience levels.
Zandoná, A.G. et al., 1998	Examiners were unaware of each other's findings	• **Visual Examination with Color Rating**<br>Inter-rater reliability = 0.62 (moderate agreement) and intra-rater reliability = 0.59 (moderate agreement)<br>• **Intraoral camera (LF and DELF)**<br>**Laser Fluorescence (LF)**<br>Inter-rater reliability = 0.68 (Substantial agreement) and intra-rater reliability = 0.63	• **Binary scoring and scoring with color rating**<br>The examiners used standardized operatory lighting<br>• **Intraoral Camera (LF and DELF)**<br>Calibrated based on the manufacturer's instructions<br>• **Histological**<br>The teeth were sectioned mesiodistally into thin slices (approximately 250 µm) and examined under a stereomicroscope at 10x magnification with incident and transmitted lighting.	• The presence of staining on fissures was noted to affect the detection accuracy of certain methods. For example, stained fissures were more easily detected with methods like Visual Color and DELF, while unstained fissures posed challenges for LF.	• Primarily, it is an in vitro design, which may not fully reflect in vivo conditions and could limit clinical applicability.<br>• They also highlighted the absence of natural oral factors like saliva and plaque, which may affect the performance of diagnostic methods in a real-world setting.<br>• The study's reliance on only two examiners may not capture the variability expected in broader clinical practice	DELF showed the highest reproducibility, with kappa values indicating substantial to almost perfect agreement between and within examiners, highlighting its consistency.

		to 0.92 (Substantial agreement)<br>**Dye-Enhanced Laser Fluorescence (DELF)**<br>Inter-rater reliability = 0.82 (Substantial to almost perfect agreement) and intra-rater reliability = 0.79 (Substantial to almost perfect agreement)				

Supplementary Table 5: JBI Appraisal Checklist

Joanna Briggs Institute (JBI) Critical Appraisal Diagnostic Accuracy Test (DTA) Checklist

(N= 28)

Article Title: Accuracy of the DIAGNOcam and bitewing radiographs in the diagnosis of cavitated proximal carious lesions in primary molars

JBI CRITICAL APPRAISAL CHECKLIST FOR DIAGNOSTIC TEST ACCURACY STUDIES

Reviewer_____FR_________________________Date______03.12.2024_________________________

Author__________ Alamoudi NM et al.________Year_2019_____ Record Number_________

	Yes	No	Unclear	Not applicable
1. Was a consecutive or random sample of patients enrolled?	☐	☑	☐	☐
2. Was a case control design avoided?	☐	☑	☐	☐
3. Did the study avoid inappropriate exclusions?	☑	☐	☐	☐
4. Were the index test results interpreted without knowledge of the results of the reference standard?	☑	☐	☐	☐
5. If a threshold was used, was it pre-specified?	☑	☐	☐	☐
6. Is the reference standard likely to correctly classify the target condition?	☐	☑	☐	☐
7. Were the reference standard results interpreted without knowledge of the results of the index test?	☑	☐	☐	☐
8. Was there an appropriate interval between index test and reference standard?	☑	☐	☐	☐
9. Did all patients receive the same reference standard?	☑	☐	☐	☐
10. Were all patients included in the analysis?	☑	☐	☐	☐

Overall appraisal: Include ☐ Exclude ☐ Seek further info ☐

Comments (Including reason for exclusion)

© JBI, 2020. All rights reserved. JBI grants use of these tools for research purposes only. All other enquiries should be sent to jbisynthesis@adelaide.edu.au.

JBI CRITICAL APPRAISAL CHECKLIST FOR DIAGNOSTIC TEST ACCURACY STUDIES

Reviewer______FR___________________________Date_______03.12.2024___________________________

Author___________ Alrayyes S. et al._________Year_2021______ Record Number__________

	Yes	No	Unclear	Not applicable
1. Was a consecutive or random sample of patients enrolled?	☐	☑	☐	☐
2. Was a case control design avoided?	☐	☑	☐	☐
3. Did the study avoid inappropriate exclusions?	☑	☐	☐	☐
4. Were the index test results interpreted without knowledge of the results of the reference standard?	☑	☐	☐	☐
5. If a threshold was used, was it pre-specified?	☑	☐	☐	☐
6. Is the reference standard likely to correctly classify the target condition?	☐	☑	☐	☐
7. Were the reference standard results interpreted without knowledge of the results of the index test?	☑	☐	☐	☐
8. Was there an appropriate interval between index test and reference standard?	☑	☐	☐	☐
9. Did all patients receive the same reference standard?	☑	☐	☐	☐
10. Were all patients included in the analysis?	☑	☐	☐	☐

Overall appraisal: Include ☐ Exclude ☐ Seek further info ☐

Comments (Including reason for exclusion)

© JBI, 2020. All rights reserved. JBI grants use of these tools for research purposes only. All other enquiries should be sent to jbisynthesis@adelaide.edu.au.

JBI CRITICAL APPRAISAL CHECKLIST FOR DIAGNOSTIC TEST ACCURACY STUDIES

Reviewer_____FR__________________________Date______03.12.2024__________________________

Author__________ Baltacioglu I.H. et al._________Year_2017_____ Record Number_________

	Yes	No	Unclear	Not applicable
1. Was a consecutive or random sample of patients enrolled?	☐	☑	☐	☐
2. Was a case control design avoided?	☐	☑	☐	☐
3. Did the study avoid inappropriate exclusions?	☑	☐	☐	☐
4. Were the index test results interpreted without knowledge of the results of the reference standard?	☑	☐	☐	☐
5. If a threshold was used, was it pre-specified?	☑	☐	☐	☐
6. Is the reference standard likely to correctly classify the target condition?	☐	☑	☐	☐
7. Were the reference standard results interpreted without knowledge of the results of the index test?	☑	☐	☐	☐
8. Was there an appropriate interval between index test and reference standard?	☑	☐	☐	☐
9. Did all patients receive the same reference standard?	☑	☐	☐	☐
10. Were all patients included in the analysis?	☑	☐	☐	☐

Overall appraisal: Include ☐ Exclude ☐ Seek further info ☐

Comments (Including reason for exclusion)

© JBI, 2020. All rights reserved. JBI grants use of these tools for research purposes only. All other enquiries should be sent to jbisynthesis@adelaide.edu.au.

JBI CRITICAL APPRAISAL CHECKLIST FOR DIAGNOSTIC TEST ACCURACY STUDIES

Reviewer_____FR________________________Date______02.12.2024________________________

Author__________Boye U. et al.__________________Year_2012_____ Record Number_________

	Yes	No	Unclear	Not applicable
1. Was a consecutive or random sample of patients enrolled?	☑	☐	☐	☐
2. Was a case control design avoided?	☑	☐	☐	☐
3. Did the study avoid inappropriate exclusions?	☑	☐	☐	☐
4. Were the index test results interpreted without knowledge of the results of the reference standard?	☑	☐	☐	☐
5. If a threshold was used, was it pre-specified?	☑	☐	☐	☐
6. Is the reference standard likely to correctly classify the target condition?	☑	☐	☐	☐
7. Were the reference standard results interpreted without knowledge of the results of the index test?	☑	☐	☐	☐
8. Was there an appropriate interval between index test and reference standard?	☑	☐	☐	☐
9. Did all patients receive the same reference standard?	☑	☐	☐	☐
10. Were all patients included in the analysis?	☑	☐	☐	☐

Overall appraisal: Include ☐ Exclude ☐ Seek further info ☐

Comments (Including reason for exclusion)

© JBI, 2020. All rights reserved. JBI grants use of these tools for research purposes only. All other enquiries should be sent to jbisynthesis@adelaide.edu.au.

JBI CRITICAL APPRAISAL CHECKLIST FOR DIAGNOSTIC TEST ACCURACY STUDIES

Reviewer_____FR_________________________Date______04.12.2024________________________

Author__________ Cuenin K. et al.,________Year_2024_____ Record Number_________

	Yes	No	Unclear	Not applicable
1. Was a consecutive or random sample of patients enrolled?	☐	✓	☐	☐
2. Was a case control design avoided?	☐	✓	☐	☐
3. Did the study avoid inappropriate exclusions?	✓	☐	☐	☐
4. Were the index test results interpreted without knowledge of the results of the reference standard?	✓	☐	☐	☐
5. If a threshold was used, was it pre-specified?	✓	☐	☐	☐
6. Is the reference standard likely to correctly classify the target condition?	☐	✓	☐	☐
7. Were the reference standard results interpreted without knowledge of the results of the index test?	✓	☐	☐	☐
8. Was there an appropriate interval between index test and reference standard?	✓	☐	☐	☐
9. Did all patients receive the same reference standard?	✓	☐	☐	☐
10. Were all patients included in the analysis?	✓	☐	☐	☐

Overall appraisal: Include ☐ Exclude ☐ Seek further info ☐

Comments (Including reason for exclusion)

© JBI, 2020. All rights reserved. JBI grants use of these tools for research purposes only. All other enquiries should be sent to jbisynthesis@adelaide.edu.au.

JBI CRITICAL APPRAISAL CHECKLIST FOR DIAGNOSTIC TEST ACCURACY STUDIES

Reviewer_____FR_________________________Date______03.12.2024________________________

Author__________ Edrees A. et al.________Year_2021_____ Record Number_________

	Yes	No	Unclear	Not applicable
1. Was a consecutive or random sample of patients enrolled?	☐	☑	☐	☐
2. Was a case control design avoided?	☐	☑	☐	☐
3. Did the study avoid inappropriate exclusions?	☑	☐	☐	☐
4. Were the index test results interpreted without knowledge of the results of the reference standard?	☑	☐	☐	☐
5. If a threshold was used, was it pre-specified?	☑	☐	☐	☐
6. Is the reference standard likely to correctly classify the target condition?	☐	☑	☐	☐
7. Were the reference standard results interpreted without knowledge of the results of the index test?	☑	☐	☐	☐
8. Was there an appropriate interval between index test and reference standard?	☑	☐	☐	☐
9. Did all patients receive the same reference standard?	☑	☐	☐	☐
10. Were all patients included in the analysis?	☑	☐	☐	☐

Overall appraisal: Include ☐ Exclude ☐ Seek further info ☐

Comments (Including reason for exclusion)

© JBI, 2020. All rights reserved. JBI grants use of these tools for research purposes only. All other enquiries should be sent to jbisynthesis@adelaide.edu.au.

JBI CRITICAL APPRAISAL CHECKLIST FOR DIAGNOSTIC TEST ACCURACY STUDIES

Reviewer_____FR___________________________Date______03.12.2024__________________________

Author___________ Elhennawy K.et al._________Year_2018_____ Record Number__________

	Yes	No	Unclear	Not applicable
1. Was a consecutive or random sample of patients enrolled?	☐	☑	☐	☐
2. Was a case control design avoided?	☐	☑	☐	☐
3. Did the study avoid inappropriate exclusions?	☑	☐	☐	☐
4. Were the index test results interpreted without knowledge of the results of the reference standard?	☑	☐	☐	☐
5. If a threshold was used, was it pre-specified?	☑	☐	☐	☐
6. Is the reference standard likely to correctly classify the target condition?	☑	☐	☐	☐
7. Were the reference standard results interpreted without knowledge of the results of the index test?	☑	☐	☐	☐
8. Was there an appropriate interval between index test and reference standard?	☑	☐	☐	☐
9. Did all patients receive the same reference standard?	☑	☐	☐	☐
10. Were all patients included in the analysis?	☑	☐	☐	☐

Overall appraisal: Include ☐ Exclude ☐ Seek further info ☐

Comments (Including reason for exclusion)

© JBI, 2020. All rights reserved. JBI grants use of these tools for research purposes only. All other enquiries should be sent to jbisynthesis@adelaide.edu.au.

Article Title: An in vitro Comparison between Laser Fluorescence and Visual Examination for Detection of Demineralization in Occlusal Pits and Fissures

JBI CRITICAL APPRAISAL CHECKLIST FOR DIAGNOSTIC TEST ACCURACY STUDIES

Reviewer_____FR________________________Date______02.12.2024________________________

Author__________ Ferreira Zandoná, A.G. et al.________Year_1998_____ Record Number_________

	Yes	No	Unclear	Not applicable
1. Was a consecutive or random sample of patients enrolled?	☐	☑	☐	☐
2. Was a case control design avoided?	☐	☑	☐	☐
3. Did the study avoid inappropriate exclusions?	☑	☐	☐	☐
4. Were the index test results interpreted without knowledge of the results of the reference standard?	☑	☐	☐	☐
5. If a threshold was used, was it pre-specified?	☑	☐	☐	☐
6. Is the reference standard likely to correctly classify the target condition?	☑	☐	☐	☐
7. Were the reference standard results interpreted without knowledge of the results of the index test?	☑	☐	☐	☐
8. Was there an appropriate interval between index test and reference standard?	☑	☐	☐	☐
9. Did all patients receive the same reference standard?	☑	☐	☐	☐
10. Were all patients included in the analysis?	☑	☐	☐	☐

Overall appraisal: Include ☐ Exclude ☐ Seek further info ☐

Comments (Including reason for exclusion)

© JBI, 2020. All rights reserved. JBI grants use of these tools for research purposes only. All other enquiries should be sent to jbisynthesis@adelaide.edu.au.

JBI CRITICAL APPRAISAL CHECKLIST FOR DIAGNOSTIC TEST ACCURACY STUDIES

Reviewer_____FR_________________________Date______03.12.2024________________________

Author__________ Iranzo-Cortes et al.________Year_2018_____ Record Number_________

	Yes	No	Unclear	Not applicable
1. Was a consecutive or random sample of patients enrolled?	☑	☐	☐	☐
2. Was a case control design avoided?	☑	☐	☐	☐
3. Did the study avoid inappropriate exclusions?	☑	☐	☐	☐
4. Were the index test results interpreted without knowledge of the results of the reference standard?	☑	☐	☐	☐
5. If a threshold was used, was it pre-specified?	☑	☐	☐	☐
6. Is the reference standard likely to correctly classify the target condition?	☑	☐	☐	☐
7. Were the reference standard results interpreted without knowledge of the results of the index test?	☑	☐	☐	☐
8. Was there an appropriate interval between index test and reference standard?	☑	☐	☐	☐
9. Did all patients receive the same reference standard?	☑	☐	☐	☐
10. Were all patients included in the analysis?	☑	☐	☐	☐

Overall appraisal: Include ☐ Exclude ☐ Seek further info ☐

Comments (Including reason for exclusion)

© JBI, 2020. All rights reserved. JBI grants use of these tools for research purposes only. All other enquiries should be sent to jbisynthesis@adelaide.edu.au.

JBI CRITICAL APPRAISAL CHECKLIST FOR DIAGNOSTIC TEST ACCURACY STUDIES

Reviewer_____FR_________________________Date______03.12.2024_________________________

Author__________ Jablonski-Momeni A. et al.________Year_2013_____ Record Number__________

	Yes	No	Unclear	Not applicable
1. Was a consecutive or random sample of patients enrolled?	☐	☑	☐	☐
2. Was a case control design avoided?	☐	☑	☐	☐
3. Did the study avoid inappropriate exclusions?	☑	☐	☐	☐
4. Were the index test results interpreted without knowledge of the results of the reference standard?	☑	☐	☐	☐
5. If a threshold was used, was it pre-specified?	☑	☐	☐	☐
6. Is the reference standard likely to correctly classify the target condition?	☐	☑	☐	☐
7. Were the reference standard results interpreted without knowledge of the results of the index test?	☑	☐	☐	☐
8. Was there an appropriate interval between index test and reference standard?	☑	☐	☐	☐
9. Did all patients receive the same reference standard?	☑	☐	☐	☐
10. Were all patients included in the analysis?	☑	☐	☐	☐

Overall appraisal: Include ☐ Exclude ☐ Seek further info ☐

Comments (Including reason for exclusion)

© JBI, 2020. All rights reserved. JBI grants use of these tools for research purposes only. All other enquiries should be sent to jbisynthesis@adelaide.edu.au.

JBI CRITICAL APPRAISAL CHECKLIST FOR DIAGNOSTIC TEST ACCURACY STUDIES

Reviewer_____FR________________________Date______02.12.2024________________________

Author__________ Jablonski-Momeni A. et al.________Year_2011_____ Record Number_________

	Yes	No	Unclear	Not applicable
1. Was a consecutive or random sample of patients enrolled?	☐	☑	☐	☐
2. Was a case control design avoided?	☐	☑	☐	☐
3. Did the study avoid inappropriate exclusions?	☑	☐	☐	☐
4. Were the index test results interpreted without knowledge of the results of the reference standard?	☑	☐	☐	☐
5. If a threshold was used, was it pre-specified?	☑	☐	☐	☐
6. Is the reference standard likely to correctly classify the target condition?	☑	☐	☐	☐
7. Were the reference standard results interpreted without knowledge of the results of the index test?	☑	☐	☐	☐
8. Was there an appropriate interval between index test and reference standard?	☑	☐	☐	☐
9. Did all patients receive the same reference standard?	☑	☐	☐	☐
10. Were all patients included in the analysis?	☑	☐	☐	☐

Overall appraisal: Include ☐ Exclude ☐ Seek further info ☐

Comments (Including reason for exclusion)

____

____

© JBI, 2020. All rights reserved. JBI grants use of these tools for research purposes only. All other enquiries should be sent to jbisynthesis@adelaide.edu.au.

Article Title: **Accuracy of an intraoral scanner with near-infrared imaging feature in detection of interproximal caries of permanent teeth: an in vivo validation**

JBI CRITICAL APPRAISAL CHECKLIST FOR DIAGNOSTIC TEST ACCURACY STUDIES

Reviewer_____FR_________________________Date______04.12.2024_________________________

Author__________ Kanar O. et al.,________Year_2023_____ Record Number_________

	Yes	No	Unclear	Not applicable
1. Was a consecutive or random sample of patients enrolled?	☐	☑	☐	☐
2. Was a case control design avoided?	☐	☑	☐	☐
3. Did the study avoid inappropriate exclusions?	☑	☐	☐	☐
4. Were the index test results interpreted without knowledge of the results of the reference standard?	☑	☐	☐	☐
5. If a threshold was used, was it pre-specified?	☑	☐	☐	☐
6. Is the reference standard likely to correctly classify the target condition?	☐	☑	☐	☐
7. Were the reference standard results interpreted without knowledge of the results of the index test?	☑	☐	☐	☐
8. Was there an appropriate interval between index test and reference standard?	☑	☐	☐	☐
9. Did all patients receive the same reference standard?	☑	☐	☐	☐
10. Were all patients included in the analysis?	☑	☐	☐	☐

Overall appraisal: Include ☐ Exclude ☐ Seek further info ☐

Comments (Including reason for exclusion)

© JBI, 2020. All rights reserved. JBI grants use of these tools for research purposes only. All other enquiries should be sent to jbisynthesis@adelaide.edu.au.

JBI CRITICAL APPRAISAL CHECKLIST FOR DIAGNOSTIC TEST ACCURACY STUDIES

Reviewer_____FR________________________Date______02.12.2024________________________

Author__________ Ko HY.et al.________Year_2015_____ Record Number_________

	Yes	No	Unclear	Not applicable
1. Was a consecutive or random sample of patients enrolled?	☐	☑	☐	☐
2. Was a case control design avoided?	☐	☑	☐	☐
3. Did the study avoid inappropriate exclusions?	☑	☐	☐	☐
4. Were the index test results interpreted without knowledge of the results of the reference standard?	☑	☐	☐	☐
5. If a threshold was used, was it pre-specified?	☑	☐	☐	☐
6. Is the reference standard likely to correctly classify the target condition?	☑	☐	☐	☐
7. Were the reference standard results interpreted without knowledge of the results of the index test?	☑	☐	☐	☐
8. Was there an appropriate interval between index test and reference standard?	☑	☐	☐	☐
9. Did all patients receive the same reference standard?	☑	☐	☐	☐
10. Were all patients included in the analysis?	☑	☐	☐	☐

Overall appraisal: Include ☐ Exclude ☐ Seek further info ☐

Comments (Including reason for exclusion)

© JBI, 2020. All rights reserved. JBI grants use of these tools for research purposes only. All other enquiries should be sent to jbisynthesis@adelaide.edu.au.

JBI CRITICAL APPRAISAL CHECKLIST FOR DIAGNOSTIC TEST ACCURACY STUDIES

Reviewer_____FR_________________________Date______02.12.2024_________________________

Author__________ Markowitz K. et al._________Year_2015_____ Record Number_________

	Yes	No	Unclear	Not applicable
1. Was a consecutive or random sample of patients enrolled?	☐	☑	☐	☐
2. Was a case control design avoided?	☐	☑	☐	☐
3. Did the study avoid inappropriate exclusions?	☑	☐	☐	☐
4. Were the index test results interpreted without knowledge of the results of the reference standard?	☑	☐	☐	☐
5. If a threshold was used, was it pre-specified?	☑	☐	☐	☐
6. Is the reference standard likely to correctly classify the target condition?	☑	☐	☐	☐
7. Were the reference standard results interpreted without knowledge of the results of the index test?	☑	☐	☐	☐
8. Was there an appropriate interval between index test and reference standard?	☑	☐	☐	☐
9. Did all patients receive the same reference standard?	☑	☐	☐	☐
10. Were all patients included in the analysis?	☑	☐	☐	☐

Overall appraisal: Include ☐ Exclude ☐ Seek further info ☐

Comments (Including reason for exclusion)

____

____

© JBI, 2020. All rights reserved. JBI grants use of these tools for research purposes only. All other enquiries should be sent to jbisynthesis@adelaide.edu.au.

JBI CRITICAL APPRAISAL CHECKLIST FOR DIAGNOSTIC TEST ACCURACY STUDIES

Reviewer_____FR_________________________Date______04.12.2024________________________

Author__________ Metzger Z. et al.,________Year_2021_____ Record Number_________

	Yes	No	Unclear	Not applicable
1. Was a consecutive or random sample of patients enrolled?	☑	☐	☐	☐
2. Was a case control design avoided?	☑	☐	☐	☐
3. Did the study avoid inappropriate exclusions?	☑	☐	☐	☐
4. Were the index test results interpreted without knowledge of the results of the reference standard?	☑	☐	☐	☐
5. If a threshold was used, was it pre-specified?	☑	☐	☐	☐
6. Is the reference standard likely to correctly classify the target condition?	☐	☑	☐	☐
7. Were the reference standard results interpreted without knowledge of the results of the index test?	☑	☐	☐	☐
8. Was there an appropriate interval between index test and reference standard?	☑	☐	☐	☐
9. Did all patients receive the same reference standard?	☑	☐	☐	☐
10. Were all patients included in the analysis?	☑	☐	☐	☐

Overall appraisal: Include ☐ Exclude ☐ Seek further info ☐

Comments (Including reason for exclusion)

© JBI, 2020. All rights reserved. JBI grants use of these tools for research purposes only. All other enquiries should be sent to jbisynthesis@adelaide.edu.au.

JBI CRITICAL APPRAISAL CHECKLIST FOR DIAGNOSTIC TEST ACCURACY STUDIES

Reviewer_____FR_________________________Date______04.12.2024_________________________

Author__________ Michou S.et al.,________Year_2021_____ Record Number_________

	Yes	No	Unclear	Not applicable
1. Was a consecutive or random sample of patients enrolled?	☑	☐	☐	☐
2. Was a case control design avoided?	☐	☑	☐	☐
3. Did the study avoid inappropriate exclusions?	☑	☐	☐	☐
4. Were the index test results interpreted without knowledge of the results of the reference standard?	☑	☐	☐	☐
5. If a threshold was used, was it pre-specified?	☑	☐	☐	☐
6. Is the reference standard likely to correctly classify the target condition?	☑	☐	☐	☐
7. Were the reference standard results interpreted without knowledge of the results of the index test?	☑	☐	☐	☐
8. Was there an appropriate interval between index test and reference standard?	☑	☐	☐	☐
9. Did all patients receive the same reference standard?	☑	☐	☐	☐
10. Were all patients included in the analysis?	☑	☐	☐	☐

Overall appraisal: Include ☐ Exclude ☐ Seek further info ☐

Comments (Including reason for exclusion)

____

____

© JBI, 2020. All rights reserved. JBI grants use of these tools for research purposes only. All other enquiries should be sent to jbisynthesis@adelaide.edu.au.

JBI CRITICAL APPRAISAL CHECKLIST FOR DIAGNOSTIC TEST ACCURACY STUDIES

Reviewer_____FR_________________________Date______04.12.2024________________________

Author__________ Michou S.et al.,________Year_2020_____ Record Number_________

	Yes	No	Unclear	Not applicable
1. Was a consecutive or random sample of patients enrolled?	☑	☐	☐	☐
2. Was a case control design avoided?	☐	☑	☐	☐
3. Did the study avoid inappropriate exclusions?	☑	☐	☐	☐
4. Were the index test results interpreted without knowledge of the results of the reference standard?	☑	☐	☐	☐
5. If a threshold was used, was it pre-specified?	☑	☐	☐	☐
6. Is the reference standard likely to correctly classify the target condition?	☑	☐	☐	☐
7. Were the reference standard results interpreted without knowledge of the results of the index test?	☑	☐	☐	☐
8. Was there an appropriate interval between index test and reference standard?	☑	☐	☐	☐
9. Did all patients receive the same reference standard?	☑	☐	☐	☐
10. Were all patients included in the analysis?	☑	☐	☐	☐

Overall appraisal: Include ☐ Exclude ☐ Seek further info ☐

Comments (Including reason for exclusion)

© JBI, 2020. All rights reserved. JBI grants use of these tools for research purposes only. All other enquiries should be sent to jbisynthesis@adelaide.edu.au.

JBI CRITICAL APPRAISAL CHECKLIST FOR DIAGNOSTIC TEST ACCURACY STUDIES

Reviewer_____FR_________________________Date______04.12.2024________________________

Author__________ Michou S.et al.,________Year_2021_____ Record Number_________

	Yes	No	Unclear	Not applicable
1. Was a consecutive or random sample of patients enrolled?	☑	☐	☐	☐
2. Was a case control design avoided?	☐	☑	☐	☐
3. Did the study avoid inappropriate exclusions?	☑	☐	☐	☐
4. Were the index test results interpreted without knowledge of the results of the reference standard?	☑	☐	☐	☐
5. If a threshold was used, was it pre-specified?	☑	☐	☐	☐
6. Is the reference standard likely to correctly classify the target condition?	☑	☐	☐	☐
7. Were the reference standard results interpreted without knowledge of the results of the index test?	☑	☐	☐	☐
8. Was there an appropriate interval between index test and reference standard?	☑	☐	☐	☐
9. Did all patients receive the same reference standard?	☑	☐	☐	☐
10. Were all patients included in the analysis?	☑	☐	☐	☐

Overall appraisal: Include ☐ Exclude ☐ Seek further info ☐

Comments (Including reason for exclusion)

© JBI, 2020. All rights reserved. JBI grants use of these tools for research purposes only. All other enquiries should be sent to jbisynthesis@adelaide.edu.au.

Article Title: Sensitivity and specificity of different diagnostic methods in occlusal caries detection of permanent teeth among paediatric patients

JBI CRITICAL APPRAISAL CHECKLIST FOR DIAGNOSTIC TEST ACCURACY STUDIES

Reviewer_____FR_________________________Date______03.12.2024_________________________

Author__________ Mokhtar IW et al.________Year_2021_____ Record Number_________

	Yes	No	Unclear	Not applicable
1. Was a consecutive or random sample of patients enrolled?	☐	☑	☐	☐
2. Was a case control design avoided?	☐	☑	☐	☐
3. Did the study avoid inappropriate exclusions?	☑	☐	☐	☐
4. Were the index test results interpreted without knowledge of the results of the reference standard?	☑	☐	☐	☐
5. If a threshold was used, was it pre-specified?	☑	☐	☐	☐
6. Is the reference standard likely to correctly classify the target condition?	☐	☑	☐	☐
7. Were the reference standard results interpreted without knowledge of the results of the index test?	☑	☐	☐	☐
8. Was there an appropriate interval between index test and reference standard?	☑	☐	☐	☐
9. Did all patients receive the same reference standard?	☑	☐	☐	☐
10. Were all patients included in the analysis?	☑	☐	☐	☐

Overall appraisal: Include ☐ Exclude ☐ Seek further info ☐

Comments (Including reason for exclusion)

© JBI, 2020. All rights reserved. JBI grants use of these tools for research purposes only. All other enquiries should be sent to jbisynthesis@adelaide.edu.au.

JBI CRITICAL APPRAISAL CHECKLIST FOR DIAGNOSTIC TEST ACCURACY STUDIES

Reviewer_____FR________________________Date______02.12.2024________________________

Author__________ Jablonski-Momeni A. et al.________Year_2012_____ Record Number_________

	Yes	No	Unclear	Not applicable
1. Was a consecutive or random sample of patients enrolled?	☐	☑	☐	☐
2. Was a case control design avoided?	☐	☑	☐	☐
3. Did the study avoid inappropriate exclusions?	☑	☐	☐	☐
4. Were the index test results interpreted without knowledge of the results of the reference standard?	☑	☐	☐	☐
5. If a threshold was used, was it pre-specified?	☑	☐	☐	☐
6. Is the reference standard likely to correctly classify the target condition?	☑	☐	☐	☐
7. Were the reference standard results interpreted without knowledge of the results of the index test?	☑	☐	☐	☐
8. Was there an appropriate interval between index test and reference standard?	☑	☐	☐	☐
9. Did all patients receive the same reference standard?	☑	☐	☐	☐
10. Were all patients included in the analysis?	☐	☑	☐	☐

Overall appraisal: Include ☐ Exclude ☐ Seek further info ☐

Comments (Including reason for exclusion)

____

____

© JBI, 2020. All rights reserved. JBI grants use of these tools for research purposes only. All other enquiries should be sent to jbisynthesis@adelaide.edu.au.

JBI CRITICAL APPRAISAL CHECKLIST FOR DIAGNOSTIC TEST ACCURACY STUDIES

Reviewer_____FR__________________________Date______04.12.2024_________________________

Author__________ Ntovas P.et al.________Year_2023_____ Record Number_________

	Yes	No	Unclear	Not applicable
1. Was a consecutive or random sample of patients enrolled?	✓	☐	☐	☐
2. Was a case control design avoided?	✓	☐	☐	☐
3. Did the study avoid inappropriate exclusions?	✓	☐	☐	☐
4. Were the index test results interpreted without knowledge of the results of the reference standard?	✓	☐	☐	☐
5. If a threshold was used, was it pre-specified?	✓	☐	☐	☐
6. Is the reference standard likely to correctly classify the target condition?	✓	☐	☐	☐
7. Were the reference standard results interpreted without knowledge of the results of the index test?	✓	☐	☐	☐
8. Was there an appropriate interval between index test and reference standard?	✓	☐	☐	☐
9. Did all patients receive the same reference standard?	✓	☐	☐	☐
10. Were all patients included in the analysis?	✓	☐	☐	☐

Overall appraisal: Include ☐ Exclude ☐ Seek further info ☐

Comments (Including reason for exclusion)

____

____

© JBI, 2020. All rights reserved. JBI grants use of these tools for research purposes only. All other enquiries should be sent to jbisynthesis@adelaide.edu.au.

Article Title: Evaluation of near-infrared digital imaging transillumination compared with bitewing radiography for proximal caries detection in children

JBI CRITICAL APPRAISAL CHECKLIST FOR DIAGNOSTIC TEST ACCURACY STUDIES

Reviewer_____FR_________________________Date______03.12.2024_________________________

Author__________ Patel J. et al.________Year_2024_____ Record Number_________

	Yes	No	Unclear	Not applicable
1. Was a consecutive or random sample of patients enrolled?	☐	☑	☐	☐
2. Was a case control design avoided?	☐	☑	☐	☐
3. Did the study avoid inappropriate exclusions?	☑	☐	☐	☐
4. Were the index test results interpreted without knowledge of the results of the reference standard?	☑	☐	☐	☐
5. If a threshold was used, was it pre-specified?	☑	☐	☐	☐
6. Is the reference standard likely to correctly classify the target condition?	☐	☑	☐	☐
7. Were the reference standard results interpreted without knowledge of the results of the index test?	☑	☐	☐	☐
8. Was there an appropriate interval between index test and reference standard?	☑	☐	☐	☐
9. Did all patients receive the same reference standard?	☑	☐	☐	☐
10. Were all patients included in the analysis?	☑	☐	☐	☐

Overall appraisal: Include ☐ Exclude ☐ Seek further info ☐

Comments (Including reason for exclusion)

© JBI, 2020. All rights reserved. JBI grants use of these tools for research purposes only. All other enquiries should be sent to jbisynthesis@adelaide.edu.au.

JBI CRITICAL APPRAISAL CHECKLIST FOR DIAGNOSTIC TEST ACCURACY STUDIES

Reviewer_____FR_________________________ Date______03.12.2024________________________

Author__________ Wang F. et al.________ Year_2022_____ Record Number_________

	Yes	No	Unclear	Not applicable
1. Was a consecutive or random sample of patients enrolled?	☐	✓	☐	☐
2. Was a case control design avoided?	☐	✓	☐	☐
3. Did the study avoid inappropriate exclusions?	✓	☐	☐	☐
4. Were the index test results interpreted without knowledge of the results of the reference standard?	✓	☐	☐	☐
5. If a threshold was used, was it pre-specified?	✓	☐	☐	☐
6. Is the reference standard likely to correctly classify the target condition?	✓	☐	☐	☐
7. Were the reference standard results interpreted without knowledge of the results of the index test?	✓	☐	☐	☐
8. Was there an appropriate interval between index test and reference standard?	✓	☐	☐	☐
9. Did all patients receive the same reference standard?	✓	☐	☐	☐
10. Were all patients included in the analysis?	✓	☐	☐	☐

Overall appraisal: Include ☐ Exclude ☐ Seek further info ☐

Comments (Including reason for exclusion)

© JBI, 2020. All rights reserved. JBI grants use of these tools for research purposes only. All other enquiries should be sent to jbisynthesis@adelaide.edu.au.

JBI CRITICAL APPRAISAL CHECKLIST FOR DIAGNOSTIC TEST ACCURACY STUDIES

Reviewer_____FR________________________Date______02.12.2024________________________

Author__________ Saffarpour A., et al.________Year_2023_____ Record Number_________

	Yes	No	Unclear	Not applicable
1. Was a consecutive or random sample of patients enrolled?	☐	☑	☐	☐
2. Was a case control design avoided?	☐	☑	☐	☐
3. Did the study avoid inappropriate exclusions?	☑	☐	☐	☐
4. Were the index test results interpreted without knowledge of the results of the reference standard?	☑	☐	☐	☐
5. If a threshold was used, was it pre-specified?	☑	☐	☐	☐
6. Is the reference standard likely to correctly classify the target condition?	☑	☐	☐	☐
7. Were the reference standard results interpreted without knowledge of the results of the index test?	☑	☐	☐	☐
8. Was there an appropriate interval between index test and reference standard?	☑	☐	☐	☐
9. Did all patients receive the same reference standard?	☑	☐	☐	☐
10. Were all patients included in the analysis?	☑	☐	☐	☐

Overall appraisal: Include ☐ Exclude ☐ Seek further info ☐

Comments (Including reason for exclusion)

© JBI, 2020. All rights reserved. JBI grants use of these tools for research purposes only. All other enquiries should be sent to jbisynthesis@adelaide.edu.au.

Article Title: Clinical effectiveness of high definition fluorescence camera in detection of initial occlusal caries

JBI CRITICAL APPRAISAL CHECKLIST FOR DIAGNOSTIC TEST ACCURACY STUDIES

Reviewer_____FR________________________Date_____02.12.2024_______________________

Author__________ Salma M. et al.________Year_2022_____ Record Number_________

	Yes	No	Unclear	Not applicable
1. Was a consecutive or random sample of patients enrolled?	☐	✓	☐	☐
2. Was a case control design avoided?	☐	✓	☐	☐
3. Did the study avoid inappropriate exclusions?	✓	☐	☐	☐
4. Were the index test results interpreted without knowledge of the results of the reference standard?	✓	☐	☐	☐
5. If a threshold was used, was it pre-specified?	✓	☐	☐	☐
6. Is the reference standard likely to correctly classify the target condition?	✓	☐	☐	☐
7. Were the reference standard results interpreted without knowledge of the results of the index test?	✓	☐	☐	☐
8. Was there an appropriate interval between index test and reference standard?	✓	☐	☐	☐
9. Did all patients receive the same reference standard?	✓	☐	☐	☐
10. Were all patients included in the analysis?	✓	☐	☐	☐

Overall appraisal: Include ☐ Exclude ☐ Seek further info ☐

Comments (Including reason for exclusion)

© JBI, 2020. All rights reserved. JBI grants use of these tools for research purposes only. All other enquiries should be sent to jbisynthesis@adelaide.edu.au.

JBI CRITICAL APPRAISAL CHECKLIST FOR DIAGNOSTIC TEST ACCURACY STUDIES

Reviewer_____FR__________________________Date______02.12.2024________________________

Author__________ Stratigaki E. et al._________Year_2020_____ Record Number_________

	Yes	No	Unclear	Not applicable
1. Was a consecutive or random sample of patients enrolled?	☐	☑	☐	☐
2. Was a case control design avoided?	☐	☑	☐	☐
3. Did the study avoid inappropriate exclusions?	☑	☐	☐	☐
4. Were the index test results interpreted without knowledge of the results of the reference standard?	☑	☐	☐	☐
5. If a threshold was used, was it pre-specified?	☑	☐	☐	☐
6. Is the reference standard likely to correctly classify the target condition?	☑	☐	☐	☐
7. Were the reference standard results interpreted without knowledge of the results of the index test?	☑	☐	☐	☐
8. Was there an appropriate interval between index test and reference standard?	☑	☐	☐	☐
9. Did all patients receive the same reference standard?	☑	☐	☐	☐
10. Were all patients included in the analysis?	☑	☐	☐	☐

Overall appraisal: Include ☐ Exclude ☐ Seek further info ☐

Comments (Including reason for exclusion)

© JBI, 2020. All rights reserved. JBI grants use of these tools for research purposes only. All other enquiries should be sent to jbisynthesis@adelaide.edu.au.

Article Title: Comparison of diagnostic effects of infrared imaging and bitewing radiography in proximal caries of permanent teeth

JBI CRITICAL APPRAISAL CHECKLIST FOR DIAGNOSTIC TEST ACCURACY STUDIES

Reviewer_____FR________________________Date______02.12.2024________________________

Author__________ Tonkaboni A. et al.________Year_2018_____ Record Number_________

	Yes	No	Unclear	Not applicable
1. Was a consecutive or random sample of patients enrolled?	☐	☑	☐	☐
2. Was a case control design avoided?	☐	☑	☐	☐
3. Did the study avoid inappropriate exclusions?	☑	☐	☐	☐
4. Were the index test results interpreted without knowledge of the results of the reference standard?	☑	☐	☐	☐
5. If a threshold was used, was it pre-specified?	☑	☐	☐	☐
6. Is the reference standard likely to correctly classify the target condition?	☑	☐	☐	☐
7. Were the reference standard results interpreted without knowledge of the results of the index test?	☑	☐	☐	☐
8. Was there an appropriate interval between index test and reference standard?	☑	☐	☐	☐
9. Did all patients receive the same reference standard?	☑	☐	☐	☐
10. Were all patients included in the analysis?	☑	☐	☐	☐

Overall appraisal: Include ☐ Exclude ☐ Seek further info ☐

Comments (Including reason for exclusion)

© JBI, 2020. All rights reserved. JBI grants use of these tools for research purposes only. All other enquiries should be sent to jbisynthesis@adelaide.edu.au.

JBI CRITICAL APPRAISAL CHECKLIST FOR DIAGNOSTIC TEST ACCURACY STUDIES

Reviewer_____FR________________________Date_____03.12.2024________________________

Author_________ Valizadeh S., et al.________Year_2022_____ Record Number_________

	Yes	No	Unclear	Not applicable
1. Was a consecutive or random sample of patients enrolled?	☐	✓	☐	☐
2. Was a case control design avoided?	☐	✓	☐	☐
3. Did the study avoid inappropriate exclusions?	✓	☐	☐	☐
4. Were the index test results interpreted without knowledge of the results of the reference standard?	✓	☐	☐	☐
5. If a threshold was used, was it pre-specified?	✓	☐	☐	☐
6. Is the reference standard likely to correctly classify the target condition?	✓	☐	☐	☐
7. Were the reference standard results interpreted without knowledge of the results of the index test?	✓	☐	☐	☐
8. Was there an appropriate interval between index test and reference standard?	✓	☐	☐	☐
9. Did all patients receive the same reference standard?	✓	☐	☐	☐
10. Were all patients included in the analysis?	✓	☐	☐	☐

Overall appraisal: Include ☐ Exclude ☐ Seek further info ☐

Comments (Including reason for exclusion)

© JBI, 2020. All rights reserved. JBI grants use of these tools for research purposes only. All other enquiries should be sent to jbisynthesis@adelaide.edu.au.

Reviewers Response on 3 Randomly Selected Articles

(N = 9)

Article Title: Accuracy of an intraoral scanner with near-infrared imaging feature in detection of interproximal caries of permanent teeth: an in vivo validation

JBI CRITICAL APPRAISAL CHECKLIST FOR DIAGNOSTIC TEST ACCURACY STUDIES

Reviewer_____FR___________________________Date______04.12.2024__________________________

Author___________ Kanar O. et al.,_________Year_2023_____ Record Number__________

	Yes	No	Unclear	Not applicable
1. Was a consecutive or random sample of patients enrolled?	☐	✓	☐	☐
2. Was a case control design avoided?	☐	✓	☐	☐
3. Did the study avoid inappropriate exclusions?	✓	☐	☐	☐
4. Were the index test results interpreted without knowledge of the results of the reference standard?	✓	☐	☐	☐
5. If a threshold was used, was it pre-specified?	✓	☐	☐	☐
6. Is the reference standard likely to correctly classify the target condition?	☐	✓	☐	☐
7. Were the reference standard results interpreted without knowledge of the results of the index test?	✓	☐	☐	☐
8. Was there an appropriate interval between index test and reference standard?	✓	☐	☐	☐
9. Did all patients receive the same reference standard?	✓	☐	☐	☐
10. Were all patients included in the analysis?	✓	☐	☐	☐

Overall appraisal: Include ☐ Exclude ☐ Seek further info ☐

Comments (Including reason for exclusion)

© JBI, 2020. All rights reserved. JBI grants use of these tools for research purposes only. All other enquiries should be sent to jbisynthesis@adelaide.edu.au.

JBI CRITICAL APPRAISAL CHECKLIST FOR DIAGNOSTIC TEST ACCURACY STUDIES

Reviewer_____FR________________________Date______02.12.2024________________________

Author__________ Ko HY.et al.________Year_2015_____ Record Number_________

	Yes	No	Unclear	Not applicable
1. Was a consecutive or random sample of patients enrolled?	☐	☑	☐	☐
2. Was a case control design avoided?	☐	☑	☐	☐
3. Did the study avoid inappropriate exclusions?	☑	☐	☐	☐
4. Were the index test results interpreted without knowledge of the results of the reference standard?	☑	☐	☐	☐
5. If a threshold was used, was it pre-specified?	☑	☐	☐	☐
6. Is the reference standard likely to correctly classify the target condition?	☑	☐	☐	☐
7. Were the reference standard results interpreted without knowledge of the results of the index test?	☑	☐	☐	☐
8. Was there an appropriate interval between index test and reference standard?	☑	☐	☐	☐
9. Did all patients receive the same reference standard?	☑	☐	☐	☐
10. Were all patients included in the analysis?	☑	☐	☐	☐

Overall appraisal: Include ☐ Exclude ☐ Seek further info ☐

Comments (Including reason for exclusion)

© JBI, 2020. All rights reserved. JBI grants use of these tools for research purposes only. All other enquiries should be sent to jbisynthesis@adelaide.edu.au.

JBI CRITICAL APPRAISAL CHECKLIST FOR DIAGNOSTIC TEST ACCURACY STUDIES

Reviewer_____FR_________________________Date______03.12.2024_________________________

Author__________ Patel J. et al.________Year_2024_____ Record Number_________

	Yes	No	Unclear	Not applicable
1. Was a consecutive or random sample of patients enrolled?	☐	☑	☐	☐
2. Was a case control design avoided?	☐	☑	☐	☐
3. Did the study avoid inappropriate exclusions?	☑	☐	☐	☐
4. Were the index test results interpreted without knowledge of the results of the reference standard?	☑	☐	☐	☐
5. If a threshold was used, was it pre-specified?	☑	☐	☐	☐
6. Is the reference standard likely to correctly classify the target condition?	☐	☑	☐	☐
7. Were the reference standard results interpreted without knowledge of the results of the index test?	☑	☐	☐	☐
8. Was there an appropriate interval between index test and reference standard?	☑	☐	☐	☐
9. Did all patients receive the same reference standard?	☑	☐	☐	☐
10. Were all patients included in the analysis?	☑	☐	☐	☐

Overall appraisal: Include ☐ Exclude ☐ Seek further info ☐

Comments (Including reason for exclusion)

© JBI, 2020. All rights reserved. JBI grants use of these tools for research purposes only. All other enquiries should be sent to jbisynthesis@adelaide.edu.au.

JBI CRITICAL APPRAISAL CHECKLIST FOR DIAGNOSTIC TEST ACCURACY STUDIES

Reviewer_____JD_________________________Date______04.12.2024__________________________

Author__________ Kanar O. et al.,_________Year_2024_____ Record Number__________

	Yes	No	Unclear	Not applicable
1. Was a consecutive or random sample of patients enrolled?	☒	☐	☐	☐
2. Was a case control design avoided?	☐	☒	☐	☐
3. Did the study avoid inappropriate exclusions?	☐	☒	☐	☐
4. Were the index test results interpreted without knowledge of the results of the reference standard?	☒	☐	☐	☐
5. If a threshold was used, was it pre-specified?	☒	☐	☐	☐
6. Is the reference standard likely to correctly classify the target condition?	☒	☐	☐	☐
7. Were the reference standard results interpreted without knowledge of the results of the index test?	☒	☐	☐	☐
8. Was there an appropriate interval between index test and reference standard?	☒	☐	☐	☐
9. Did all patients receive the same reference standard?	☒	☐	☐	☐
10. Were all patients included in the analysis?	☒	☐	☐	☐

Overall appraisal: Include ☐ Exclude ☐ Seek further info ☐

Comments (Including reason for exclusion)

____

____

© JBI, 2020. All rights reserved. JBI grants use of these tools for research purposes only. All other enquiries should be sent to jbisynthesis@adelaide.edu.au.

JBI CRITICAL APPRAISAL CHECKLIST FOR DIAGNOSTIC TEST ACCURACY STUDIES

Reviewer_____JD________________________Date______04.12.2024_________________________

Author__________ Ko YH et al.,________Year_2015_____ Record Number_________

		Yes	No	Unclear	Not applicable
1.	Was a consecutive or random sample of patients enrolled?	X	☐	☐	☐
2.	Was a case control design avoided?	☐	X	☐	☐
3.	Did the study avoid inappropriate exclusions?	☐	X	☐	☐
4.	Were the index test results interpreted without knowledge of the results of the reference standard?	X	☐	☐	☐
5.	If a threshold was used, was it pre-specified?	X	☐	☐	☐
6.	Is the reference standard likely to correctly classify the target condition?	X	☐	☐	☐
7.	Were the reference standard results interpreted without knowledge of the results of the index test?	X	☐	☐	☐
8.	Was there an appropriate interval between index test and reference standard?	X	☐	☐	☐
9.	Did all patients receive the same reference standard?	X	☐	☐	☐
10.	Were all patients included in the analysis?	X	☐	☐	☐

Overall appraisal: Include ☐ Exclude ☐ Seek further info ☐

Comments (Including reason for exclusion)

____

____

© JBI, 2020. All rights reserved. JBI grants use of these tools for research purposes only. All other enquiries should be sent to jbisynthesis@adelaide.edu.au.

JBI CRITICAL APPRAISAL CHECKLIST FOR DIAGNOSTIC TEST ACCURACY STUDIES

Reviewer_____JD________________________Date______04.12.2024________________________

Author__________ Patel J. et al.,________Year_2024_____ Record Number_________

	Yes	No	Unclear	Not applicable
1. Was a consecutive or random sample of patients enrolled?	☒	☐	☐	☐
2. Was a case control design avoided?	☐	☒	☐	☐
3. Did the study avoid inappropriate exclusions?	☐	☒	☐	☐
4. Were the index test results interpreted without knowledge of the results of the reference standard?	☒	☐	☐	☐
5. If a threshold was used, was it pre-specified?	☒	☐	☐	☐
6. Is the reference standard likely to correctly classify the target condition?	☒	☐	☐	☐
7. Were the reference standard results interpreted without knowledge of the results of the index test?	☒	☐	☐	☐
8. Was there an appropriate interval between index test and reference standard?	☒	☐	☐	☐
9. Did all patients receive the same reference standard?	☒	☐	☐	☐
10. Were all patients included in the analysis?	☒	☐	☐	☐

Overall appraisal: Include ☐ Exclude ☐ Seek further info ☐

Comments (Including reason for exclusion)

____

____

© JBI, 2020. All rights reserved. JBI grants use of these tools for research purposes only. All other enquiries should be sent to jbisynthesis@adelaide.edu.au.

JBI CRITICAL APPRAISAL CHECKLIST FOR DIAGNOSTIC TEST ACCURACY STUDIES

Reviewer_______________TH_____________________Date_________03.12.24________________________

Kanar Ö, Tağtekin D, Korkut B. Accuracy of an intraoral scanner with near-infrared imaging feature in detection of interproximal caries of permanent teeth: An in vivo validation. Journal of Esthetic and Restorative Dentistry. 2024 Jun;36(6):845-57.

	Yes	No	Unclear	Not applicable
1. Was a consecutive or random sample of patients enrolled?	X	☐	☐	☐
2. Was a case control design avoided?	X	☐	☐	☐
3. Did the study avoid inappropriate exclusions?	☐	X	☐	☐
4. Were the index test results interpreted without knowledge of the results of the reference standard?	X	☐	☐	☐
5. If a threshold was used, was it pre-specified?	X	☐	☐	☐
6. Is the reference standard likely to correctly classify the target condition?	☐	X	☐	☐
7. Were the reference standard results interpreted without knowledge of the results of the index test?	X	☐	☐	☐
8. Was there an appropriate interval between index test and reference standard?	X	☐	☐	☐
9. Did all patients receive the same reference standard?	X	☐	☐	☐
10. Were all patients included in the analysis?	X	☐	☐	☐

Overall appraisal: Include ☐ Exclude ☐ Seek further info ☐

Comments (Including reason for exclusion)

© JBI, 2020. All rights reserved. JBI grants use of these tools for research purposes only. All other enquiries should be sent to jbisynthesis@adelaide.edu.au.

JBI CRITICAL APPRAISAL CHECKLIST FOR DIAGNOSTIC TEST ACCURACY STUDIES

Reviewer___________TH______________________Date_____03.12.24___________________________

Ko HY, Kang SM, Kim HE, Kwon HK, Kim BI. Validation of quantitative light-induced fluorescence-digital (QLF-D) for the detection of approximal caries in vitro. Journal of dentistry. 2015 May 1;43(5):568-75.

	Yes	No	Unclear	Not applicable
1. Was a consecutive or random sample of patients enrolled?	X	☐	☐	☐
2. Was a case control design avoided?	☐	X	☐	☐
3. Did the study avoid inappropriate exclusions?	☐	X	☐	☐
4. Were the index test results interpreted without knowledge of the results of the reference standard?	X	☐	☐	☐
5. If a threshold was used, was it pre-specified?	X	☐	☐	☐
6. Is the reference standard likely to correctly classify the target condition?	X	☐	☐	☐
7. Were the reference standard results interpreted without knowledge of the results of the index test?	X	☐	☐	☐
8. Was there an appropriate interval between index test and reference standard?	X	☐	☐	☐
9. Did all patients receive the same reference standard?	X	☐	☐	☐
10. Were all patients included in the analysis?	X	☐	☐	☐

Overall appraisal: Include ☐ Exclude ☐ Seek further info ☐

Comments (Including reason for exclusion)

___

___

© JBI, 2020. All rights reserved. JBI grants use of these tools for research purposes only. All other enquiries should be sent to jbisynthesis@adelaide.edu.au.

JBI CRITICAL APPRAISAL CHECKLIST FOR DIAGNOSTIC TEST ACCURACY STUDIES

Reviewer___________TH______________________Date_____03.12.24___________________________

Patel J, Vannemreddy A, Goh YJ, Francis Y, Anthonappa R. Evaluation of near-infrared digital imaging transillumination compared with bitewing radiography for proximal caries detection in children. International Journal of Paediatric Dentistry. 2024 May 20.

	Yes	No	Unclear	Not applicable
1. Was a consecutive or random sample of patients enrolled?	X	☐	☐	☐
2. Was a case control design avoided?	X	☐	☐	☐
3. Did the study avoid inappropriate exclusions?	☐	X	☐	☐
4. Were the index test results interpreted without knowledge of the results of the reference standard?	X	☐	☐	☐
5. If a threshold was used, was it pre-specified?	X	☐	☐	☐
6. Is the reference standard likely to correctly classify the target condition?	☐	☐	X	☐
7. Were the reference standard results interpreted without knowledge of the results of the index test?	X	☐	☐	☐
8. Was there an appropriate interval between index test and reference standard?	X	☐	☐	☐
9. Did all patients receive the same reference standard?	X	☐	☐	☐
10. Were all patients included in the analysis?	X	☐	☐	☐

Overall appraisal: Include ☐ Exclude ☐ Seek further info ☐

Comments (Including reason for exclusion)

© JBI, 2020. All rights reserved. JBI grants use of these tools for research purposes only. All other enquiries should be sent to jbisynthesis@adelaide.edu.au.

Supplementary table 6: Code Script for meta-analysis

```
!pip install numpy pandas matplotlib scipy
```

```
from google.colab import files
uploaded = files.upload()

import pandas as pd
# Load dataset
data = pd.read_csv("Data(empro).csv")

# Display the first few rows
print(data.head())
```

```
import numpy as np

# Logit transformation function
def logit(p):
    return np.log(p / (1 - p))

# Extract sensitivity and specificity
sensitivity = data["Sensitivity"]
specificity = data["Specificity"]

# Logit transform sensitivity and specificity
logit_sens = logit(sensitivity)
logit_spec = logit(specificity)

# Extract sample sizes for weights
n_positive = data["TP"] + data["FN"]
n_negative = data["TN"] + data["FP"]

# Display transformed data
data["Logit Sensitivity"] = logit_sens
data["Logit Specificity"] = logit_spec
print(data[["Sensitivity", "Specificity", "Logit Sensitivity", "Logit Specificity"]].head())
```

```
from scipy.optimize import minimize

# Define likelihood function
def bivariate_likelihood(params):
    mu_sens, mu_spec, tau2_sens, tau2_spec, rho = params
    tau_sens = np.sqrt(tau2_sens)
    tau_spec = np.sqrt(tau2_spec)
    log_likelihood = 0

    for i in range(len(data)):
        var_within_sens = 1 / n_positive[i]
        var_within_spec = 1 / n_negative[i]

        cov_within = rho * np.sqrt(var_within_sens * var_within_spec)
        cov_matrix = np.array([[var_within_sens + tau2_sens, cov_within],
                               [cov_within, var_within_spec + tau2_spec]])

        inv_cov_matrix = np.linalg.inv(cov_matrix)
        det_cov_matrix = np.linalg.det(cov_matrix)
```

```
        diff = np.array([logit_sens[i] - mu_sens, logit_spec[i] - mu_spec])

        log_likelihood += -0.5 * (np.log(det_cov_matrix) +
                          diff.T @ inv_cov_matrix @ diff)
    return -log_likelihood  # Minimize negative log-likelihood
```

```
# Initial parameter guesses
init_params = [0, 0, 0.1, 0.1, 0]  # [mu_sens, mu_spec, tau2_sens, tau2_spec, rho]

# Perform optimization
result = minimize(bivariate_likelihood, init_params, method="L-BFGS-B",
               bounds=[(-5, 5), (-5, 5), (0, 5), (0, 5), (-1, 1)])

# Extract results
mu_sens, mu_spec, tau2_sens, tau2_spec, rho = result.x
pooled_sens = 1 / (1 + np.exp(-mu_sens))  # Inverse logit
pooled_spec = 1 / (1 + np.exp(-mu_spec))  # Inverse logit
tau_sens = np.sqrt(tau2_sens)
tau_spec = np.sqrt(tau2_spec)

# Print results
print(f"Pooled Sensitivity: {pooled_sens:.3f}")
print(f"Pooled Specificity: {pooled_spec:.3f}")
print(f"Between-Study Variance (Sensitivity): {tau_sens:.3f}")
print(f"Between-Study Variance (Specificity): {tau_spec:.3f}")
print(f"Correlation (Sensitivity-Specificity): {rho:.3f}")
```

```
from scipy.stats import f_oneway
# Filter the data to include only relevant columns
data_filtered = data[['Diagnostic Category', 'Sensitivity', 'Specificity']]

# Group data by 'Diagnostic Category' for Welch's ANOVA
categories = data_filtered['Diagnostic Category'].unique()

# Prepare the data for sensitivity
sensitivity_groups = [data_filtered.loc[data_filtered['Diagnostic Category'] == cat, 'Sensitivity'] for cat in
categories]

# Perform Welch's ANOVA for sensitivity
sensitivity_anova = f_oneway(*sensitivity_groups)

# Prepare the data for specificity
specificity_groups = [data_filtered.loc[data_filtered['Diagnostic Category'] == cat, 'Specificity'] for cat in
categories]

# Perform Welch's ANOVA for specificity
specificity_anova = f_oneway(*specificity_groups)

# Print results
print("Welch's ANOVA for Sensitivity:")
print(f"F-statistic: {sensitivity_anova.statistic}, p-value: {sensitivity_anova.pvalue}")

print("\nWelch's ANOVA for Specificity:")
print(f"F-statistic: {specificity_anova.statistic}, p-value: {specificity_anova.pvalue}")
```

```
!pip install pingouin
!pip install scikit-posthocs
```

```
import pingouin as pg
import pandas as pd
import scikit_posthocs as sp
# Games-Howell test for Sensitivity
print("Games-Howell Post-Hoc Test for Sensitivity:")
sensitivity_posthoc = pg.pairwise_gameshowell(
    dv='Sensitivity', between='Diagnostic Category', data=data
)
print(sensitivity_posthoc)
# Save results to a CSV file
sensitivity_posthoc.to_csv("Diagnostic_Methods_lesion.csv", index=False)

# Games-Howell test for Specificity
print("\nGames-Howell Post-Hoc Test for Specificity:")
specificity_posthoc = pg.pairwise_gameshowell(
    dv='Specificity', between='Diagnostic Category', data=data
)
print(specificity_posthoc)
specificity_posthoc.to_csv("Diagnostic_sp_lesion.csv", index=False)
```

```
import scipy.stats as stats

# Group data by Diagnostic Category and Lesion Location for Sensitivity and Specificity
sensitivity_groups = data.groupby(['Diagnostic Category', 'Lesion location'])['Sensitivity'].apply(list)
specificity_groups = data.groupby(['Diagnostic Category', 'Lesion location'])['Specificity'].apply(list)

# Perform Welch ANOVA for Sensitivity
sensitivity_groups_list = [group for group in sensitivity_groups]
sensitivity_anova_result = stats.f_oneway(*sensitivity_groups_list)

# Perform Welch ANOVA for Specificity
specificity_groups_list = [group for group in specificity_groups]
specificity_anova_result = stats.f_oneway(*specificity_groups_list)

# Display results
print("Welch ANOVA for Sensitivity:")
print("Statistic:", sensitivity_anova_result.statistic)
print("P-value:", sensitivity_anova_result.pvalue)

print("\nWelch ANOVA for Specificity:")
print("Statistic:", specificity_anova_result.statistic)
print("P-value:", specificity_anova_result.pvalue)
```

```
import pandas as pd
import numpy as np
import scipy.stats as stats
import pingouin as pg
# Rename columns to remove spaces (to avoid syntax issues)
data.columns = data.columns.str.replace(' ', '_')
# Convert 'Diagnostic_Category' and 'Lesion_location' to categorical types
data['Diagnostic_Category'] = data['Diagnostic_Category'].astype('category')
data['Lesion_location'] = data['Lesion_location'].astype('category')

# Split data by Lesion Location (occlusal and proximal)
occlusal_data = data[data['Lesion_location'] == 'Occlusal']
proximal_data = data[data['Lesion_location'] == 'Proximal']
```

```
# Perform Welch ANOVA for Sensitivity by Lesion Location
sensitivity_occlusal_groups = occlusal_data.groupby('Diagnostic_Category')['Sensitivity'].apply(list)
sensitivity_proximal_groups = proximal_data.groupby('Diagnostic_Category')['Sensitivity'].apply(list)
# Perform Welch ANOVA for Sensitivity (Occlusal and Proximal)
sensitivity_occlusal_anova_result = stats.f_oneway(*sensitivity_occlusal_groups)
sensitivity_proximal_anova_result = stats.f_oneway(*sensitivity_proximal_groups)

# Display ANOVA results for Sensitivity
print("Welch ANOVA for Sensitivity (Occlusal Lesions):")
print("Statistic:", sensitivity_occlusal_anova_result.statistic)
print("P-value:", sensitivity_occlusal_anova_result.pvalue)

print("\nWelch ANOVA for Sensitivity (Proximal Lesions):")
print("Statistic:", sensitivity_proximal_anova_result.statistic)
print("P-value:", sensitivity_proximal_anova_result.pvalue)

# Now perform the Games-Howell test for Sensitivity for both lesion locations
print("\nGames-Howell Test for Sensitivity (Occlusal Lesions):")
gameshowell_occlusal_sensitivity = pg.pairwise_gameshowell(dv='Sensitivity',
between='Diagnostic_Category', data=occlusal_data)
print(gameshowell_occlusal_sensitivity)

print("\nGames-Howell Test for Sensitivity (Proximal Lesions):")
gameshowell_proximal_sensitivity = pg.pairwise_gameshowell(dv='Sensitivity',
between='Diagnostic_Category', data=proximal_data)
print(gameshowell_proximal_sensitivity)

# Perform Welch ANOVA for Specificity by Lesion Location
specificity_occlusal_groups = occlusal_data.groupby('Diagnostic_Category')['Specificity'].apply(list)
specificity_proximal_groups = proximal_data.groupby('Diagnostic_Category')['Specificity'].apply(list)

# Perform Welch ANOVA for Specificity (Occlusal and Proximal)
specificity_occlusal_anova_result = stats.f_oneway(*specificity_occlusal_groups)
specificity_proximal_anova_result = stats.f_oneway(*specificity_proximal_groups)

# Display ANOVA results for Specificity
print("\nWelch ANOVA for Specificity (Occlusal Lesions):")
print("Statistic:", specificity_occlusal_anova_result.statistic)
print("P-value:", specificity_occlusal_anova_result.pvalue)

print("\nWelch ANOVA for Specificity (Proximal Lesions):")
print("Statistic:", specificity_proximal_anova_result.statistic)
print("P-value:", specificity_proximal_anova_result.pvalue)

# Now perform the Games-Howell test for Specificity for both lesion locations
print("\nGames-Howell Test for Specificity (Occlusal Lesions):")
gameshowell_occlusal_specificity = pg.pairwise_gameshowell(dv='Specificity',
between='Diagnostic_Category', data=occlusal_data)
print(gameshowell_occlusal_specificity)

print("\nGames-Howell Test for Specificity (Proximal Lesions):")
gameshowell_proximal_specificity = pg.pairwise_gameshowell(dv='Specificity',
between='Diagnostic_Category', data=proximal_data)
print(gameshowell_proximal_specificity)
```

```
# Save results to CSV
gameshowell_occlusal_sensitivity.to_csv('gameshowell_occlusal_sensitivity_results.csv', index=False)
gameshowell_proximal_sensitivity.to_csv('gameshowell_proximal_sensitivity_results.csv', index=False)
gameshowell_occlusal_specificity.to_csv('gameshowell_occlusal_specificity_results.csv', index=False)
gameshowell_proximal_specificity.to_csv('gameshowell_proximal_specificity_results.csv', index=False)

print("\nGames-Howell results have been saved to CSV files.")
```

```
import numpy as np
from scipy.optimize import minimize
import pandas as pd

# Clean and standardize the "Lesion type" column
data["Lesion type"] = data["Lesion_type"].str.strip()

# Logit transformation function
def logit(p):
    return np.log(p / (1 - p))

# Inverse logit transformation function
def inverse_logit(logit_p):
    return 1 / (1 + np.exp(-logit_p))

# Add logit-transformed sensitivity and specificity
data["Logit Sensitivity"] = logit(data["Sensitivity"])
data["Logit Specificity"] = logit(data["Specificity"])

# Group data by lesion type
lesion_groups = data.groupby("Lesion type")
# Random-effects model likelihood function
def random_effects_likelihood(params, effects, variances):
    mu, tau2 = params  # mu: pooled effect, tau2: between-study variance
    tau = np.sqrt(tau2)
    likelihood = 0

    for effect, var in zip(effects, variances):
        total_var = var + tau2
        likelihood += 0.5 * (np.log(total_var) + ((effect - mu) ** 2) / total_var)

    return likelihood
# Initialize results list
device_results = []

# Analyze each lesion type subgroup and diagnostic device
for lesion, group in lesion_groups:
    devices = group.groupby("Diagnostic_Category")
    for device, subgroup in devices:
        # Perform random-effects analysis for sensitivity
        effects_sens = subgroup["Logit Sensitivity"]
        variances_sens = subgroup["SE(Sensitivity)"] ** 2

        init_params = [0, 0.1]  # Initial guesses for mu and tau2
        result_sens = minimize(random_effects_likelihood, init_params, args=(effects_sens,
variances_sens),
```

```
                        method="L-BFGS-B", bounds=[(-5, 5), (0, 5)])

        pooled_logit_sens = result_sens.x[0]
        tau2_sens = result_sens.x[1]
        pooled_sens = inverse_logit(pooled_logit_sens)  # Back-transform

        # Standard error and 95% CI for sensitivity
        se_pooled_logit_sens = np.sqrt(1 / np.sum(1 / (variances_sens + tau2_sens)))
        lower_logit_sens_ci = pooled_logit_sens - 1.96 * se_pooled_logit_sens
        upper_logit_sens_ci = pooled_logit_sens + 1.96 * se_pooled_logit_sens
        lower_sens_ci = inverse_logit(lower_logit_sens_ci)
        upper_sens_ci = inverse_logit(upper_logit_sens_ci)

        # Perform random-effects analysis for specificity
        effects_spec = subgroup["Logit Specificity"]
        variances_spec = subgroup["SE(Specificity)"] ** 2

        result_spec = minimize(random_effects_likelihood, init_params, args=(effects_spec,
variances_spec),
                        method="L-BFGS-B", bounds=[(-5, 5), (0, 5)])

        pooled_logit_spec = result_spec.x[0]
        tau2_spec = result_spec.x[1]
        pooled_spec = inverse_logit(pooled_logit_spec)  # Back-transform

        # Standard error and 95% CI for specificity
        se_pooled_logit_spec = np.sqrt(1 / np.sum(1 / (variances_spec + tau2_spec)))
        lower_logit_spec_ci = pooled_logit_spec - 1.96 * se_pooled_logit_spec
        upper_logit_spec_ci = pooled_logit_spec + 1.96 * se_pooled_logit_spec
        lower_spec_ci = inverse_logit(lower_logit_spec_ci)
        upper_spec_ci = inverse_logit(upper_logit_spec_ci)

        # Store device-specific results
        device_results.append({
            "Lesion Type": lesion,
            "Diagnostic Device": device,
            "Pooled Sensitivity": pooled_sens,
            "Lower 95% CI Sensitivity": lower_sens_ci,
            "Upper 95% CI Sensitivity": upper_sens_ci,
            "Tau^2 (Sensitivity)": tau2_sens,
            "Pooled Specificity": pooled_spec,
            "Lower 95% CI Specificity": lower_spec_ci,
            "Upper 95% CI Specificity": upper_spec_ci,
            "Tau^2 (Specificity)": tau2_spec
        })

        print(f"Lesion: {lesion}, Device: {device}")
        print(f"Pooled Sensitivity: {pooled_sens:.3f} (95% CI: {lower_sens_ci:.3f}, {upper_sens_ci:.3f})")
        print(f"Pooled Specificity: {pooled_spec:.3f} (95% CI: {lower_spec_ci:.3f}, {upper_spec_ci:.3f})")

# Convert to a DataFrame
device_results_df = pd.DataFrame(device_results)

# Save to CSV
device_results_df.to_csv("Device_Performance_By_Lesion_Type.csv", index=False)
```

```
# Print summary
print(device_results_df)
```

```
import pandas as pd
import matplotlib.pyplot as plt

# Load the dataset
data = pd.read_csv('Device_Performance_By_Lesion_Type.csv')  # Update with the correct file path if necessary

# Prepare the data for plotting
lesion_types = data['Lesion Type'].unique()

# Create subplots for each lesion type
fig, axes = plt.subplots(len(lesion_types), 2, figsize=(18, len(lesion_types) * 2), sharex='col', sharey='row')

for i, lesion in enumerate(lesion_types):
    subset = data[data['Lesion Type'] == lesion]
    devices = subset['Diagnostic Device']
    sensitivity_values = subset['Pooled Sensitivity']
    sensitivity_cis = list(zip(subset['Lower 95% CI Sensitivity'], subset['Upper 95% CI Sensitivity']))
    specificity_values = subset['Pooled Specificity']
    specificity_cis = list(zip(subset['Lower 95% CI Specificity'], subset['Upper 95% CI Specificity']))

    # Sensitivity plot
    for j in range(len(devices)):
        axes[i, 0].errorbar(
            sensitivity_values.iloc[j], j,
            xerr=[[sensitivity_values.iloc[j] - sensitivity_cis[j][0]], [sensitivity_cis[j][1] - sensitivity_values.iloc[j]]],
            fmt='o', capsize=3, color='black'
        )
        axes[i, 0].text(
            1.02, j,
            f"{sensitivity_values.iloc[j]:.2f} [{sensitivity_cis[j][0]:.2f}, {sensitivity_cis[j][1]:.2f}]",
            verticalalignment='center', fontsize=8
        )

    # Specificity plot
    for j in range(len(devices)):
        axes[i, 1].errorbar(
            specificity_values.iloc[j], j,
            xerr=[[specificity_values.iloc[j] - specificity_cis[j][0]], [specificity_cis[j][1] - specificity_values.iloc[j]]],
            fmt='o', capsize=3, color='black'
        )
        axes[i, 1].text(
            1.02, j,
            f"{specificity_values.iloc[j]:.2f} [{specificity_cis[j][0]:.2f}, {specificity_cis[j][1]:.2f}]",
            verticalalignment='center', fontsize=8
        )

    axes[i, 0].set_yticks(range(len(devices)))
    axes[i, 0].set_yticklabels(devices)
```

```
    axes[i, 0].set_xlim(0, 1.2)
    axes[i, 0].set_title(f"Sensitivity: {lesion}", fontsize=10)
    axes[i, 0].grid(True, linestyle='--', alpha=0.6)

    axes[i, 1].set_xlim(0, 1.2)
    axes[i, 1].set_title(f"Specificity: {lesion}", fontsize=10)
    axes[i, 1].grid(True, linestyle='--', alpha=0.6)

# Adjust layout and display the plot
plt.tight_layout()
plt.show()
```

```
import numpy as np
from scipy.optimize import minimize
import pandas as pd

# Load dataset
data = pd.read_csv("Data(empro).csv")
# Logit transformation function
def logit(p):
    return np.log(p / (1 - p))

# Inverse logit transformation function
def inverse_logit(logit_p):
    return 1 / (1 + np.exp(-logit_p))

# Add logit-transformed sensitivity and specificity
data["Logit Sensitivity"] = logit(data["Sensitivity"])
data["Logit Specificity"] = logit(data["Specificity"])

# Random-effects model likelihood function
def random_effects_likelihood(params, effects, variances):
    mu, tau2 = params  # mu: pooled effect, tau2: between-study variance
    likelihood = 0
    for effect, var in zip(effects, variances):
        total_var = var + tau2
        likelihood += 0.5 * (np.log(total_var) + ((effect - mu) ** 2) / total_var)
    return likelihood

# Initialize results list
results = []

# Group by diagnostic device
for device, group in data.groupby("Diagnostic Category"):
    # Group by examiners within each device
    for examiner, subgroup in group.groupby("Examiners"):
        # Perform random-effects analysis for sensitivity
        effects_sens = subgroup["Logit Sensitivity"]
        variances_sens = subgroup["SE(Sensitivity)"] ** 2

        # Initial guesses for mu and tau2
        init_params = [0, 0.1]
        result_sens = minimize(random_effects_likelihood, init_params, args=(effects_sens,
variances_sens),
                               method="L-BFGS-B", bounds=[(-5, 5), (0, 5)])
```

```
        pooled_logit_sens = result_sens.x[0]
        tau2_sens = result_sens.x[1]
        pooled_sens = inverse_logit(pooled_logit_sens)  # Back-transform

        # Calculate standard error and confidence intervals for sensitivity
        se_pooled_logit_sens = np.sqrt(1 / np.sum(1 / (variances_sens + tau2_sens)))
        lower_logit_sens_ci = pooled_logit_sens - 1.96 * se_pooled_logit_sens
        upper_logit_sens_ci = pooled_logit_sens + 1.96 * se_pooled_logit_sens
        lower_sens_ci = inverse_logit(lower_logit_sens_ci)
        upper_sens_ci = inverse_logit(upper_logit_sens_ci)

        # Perform random-effects analysis for specificity
        effects_spec = subgroup["Logit Specificity"]
        variances_spec = subgroup["SE(Specificity)"] ** 2

        result_spec = minimize(random_effects_likelihood, init_params, args=(effects_spec,
variances_spec),
                               method="L-BFGS-B", bounds=[(-5, 5), (0, 5)])

        pooled_logit_spec = result_spec.x[0]
        tau2_spec = result_spec.x[1]
        pooled_spec = inverse_logit(pooled_logit_spec)  # Back-transform

        # Calculate standard error and confidence intervals for specificity
        se_pooled_logit_spec = np.sqrt(1 / np.sum(1 / (variances_spec + tau2_spec)))
        lower_logit_spec_ci = pooled_logit_spec - 1.96 * se_pooled_logit_spec
        upper_logit_spec_ci = pooled_logit_spec + 1.96 * se_pooled_logit_spec
        lower_spec_ci = inverse_logit(lower_logit_spec_ci)
        upper_spec_ci = inverse_logit(upper_logit_spec_ci)

        # Store results
        results.append({
            "Diagnostic Device": device,
            "Examiner": examiner,
            "Pooled Sensitivity": pooled_sens,
            "Lower 95% CI Sensitivity": lower_sens_ci,
            "Upper 95% CI Sensitivity": upper_sens_ci,
            "Tau^2 (Sensitivity)": tau2_sens,
            "Pooled Specificity": pooled_spec,
            "Lower 95% CI Specificity": lower_spec_ci,
            "Upper 95% CI Specificity": upper_spec_ci,
            "Tau^2 (Specificity)": tau2_spec
        })

# Convert results to DataFrame
results_df = pd.DataFrame(results)

# Save results to CSV for review
results_df.to_csv("Subgroup_Analysis_Examiner.csv", index=False)

# Display the results
print(results_df)
```

```
import matplotlib.pyplot as plt
data2 = pd.read_csv("/content/Subgroup_Analysis_Examiner.csv")
# Prepare the data for plotting
```

```
devices = data2['Diagnostic Device'].unique()

# Create subplots for each diagnostic device
fig, axes = plt.subplots(len(devices), 2, figsize=(18, len(devices) * 2), sharex='col', sharey='row')

for i, device in enumerate(devices):
    subset = data2[data2['Diagnostic Device'] == device]
    examiners = subset['Examiner']
    sensitivity_values = subset['Pooled Sensitivity']
    sensitivity_cis = list(zip(subset['Lower 95% CI Sensitivity'], subset['Upper 95% CI Sensitivity']))
    specificity_values = subset['Pooled Specificity']
    specificity_cis = list(zip(subset['Lower 95% CI Specificity'], subset['Upper 95% CI Specificity']))

    # Sensitivity Forest Plot
    for j in range(len(examiners)):
        axes[i, 0].errorbar(
            sensitivity_values.iloc[j], j,
            xerr=[[sensitivity_values.iloc[j] - sensitivity_cis[j][0]], [sensitivity_cis[j][1] -
sensitivity_values.iloc[j]]],
            fmt='o', capsize=3, color='black'
        )
        axes[i, 0].text(
            1.02, j,
            f"{sensitivity_values.iloc[j]:.2f} [{sensitivity_cis[j][0]:.2f}, {sensitivity_cis[j][1]:.2f}]",
            verticalalignment='center', fontsize=8
        )

    # Specificity Forest Plot
    for j in range(len(examiners)):
        axes[i, 1].errorbar(
            specificity_values.iloc[j], j,
            xerr=[[specificity_values.iloc[j] - specificity_cis[j][0]], [specificity_cis[j][1] -
specificity_values.iloc[j]]],
            fmt='o', capsize=3, color='black'
        )
        axes[i, 1].text(
            1.02, j,
            f"{specificity_values.iloc[j]:.2f} [{specificity_cis[j][0]:.2f}, {specificity_cis[j][1]:.2f}]",
            verticalalignment='center', fontsize=8
        )

    axes[i, 0].set_yticks(range(len(examiners)))
    axes[i, 0].set_yticklabels(examiners)
    axes[i, 0].set_xlim(0, 1.2)
    axes[i, 0].set_title(f"Sensitivity: {device}", fontsize=10)
    axes[i, 0].grid(True, linestyle='--', alpha=0.6)

    axes[i, 1].set_xlim(0, 1.2)
    axes[i, 1].set_title(f"Specificity: {device}", fontsize=10)
    axes[i, 1].grid(True, linestyle='--', alpha=0.6)

# Adjust layout and display the plot
plt.tight_layout()
plt.show()
```
